# Supplementary material for: Imputation of KIR Types from SNP Variation Data
Source: Am J Hum Genet. 2015 Oct 1;97(4):593–607. doi: 10.1016/j.ajhg.2015.09.005 (PMC4596914; doi:10.1016/j.ajhg.2015.09.005)
Supplement: Document S1. Figures S1–S12 and Tables S1–S5 [file mmc1.pdf]

The American Journal of Human Genetics

Supplemental Data

Imputation of KIR Types from SNP Variation Data

Damjan Vukcevic, James A. Traherne, Sigrid Næss, Eva Ellinghaus, Yoichiro Kamatani, Alexander Dilthey, Mark Lathrop, Tom H. Karlsen, Andre Franke, Miriam Moffatt, William Cookson, John Trowsdale, Gil McVean, Stephen Sawcer, and Stephen Leslie

Supplemental Figures

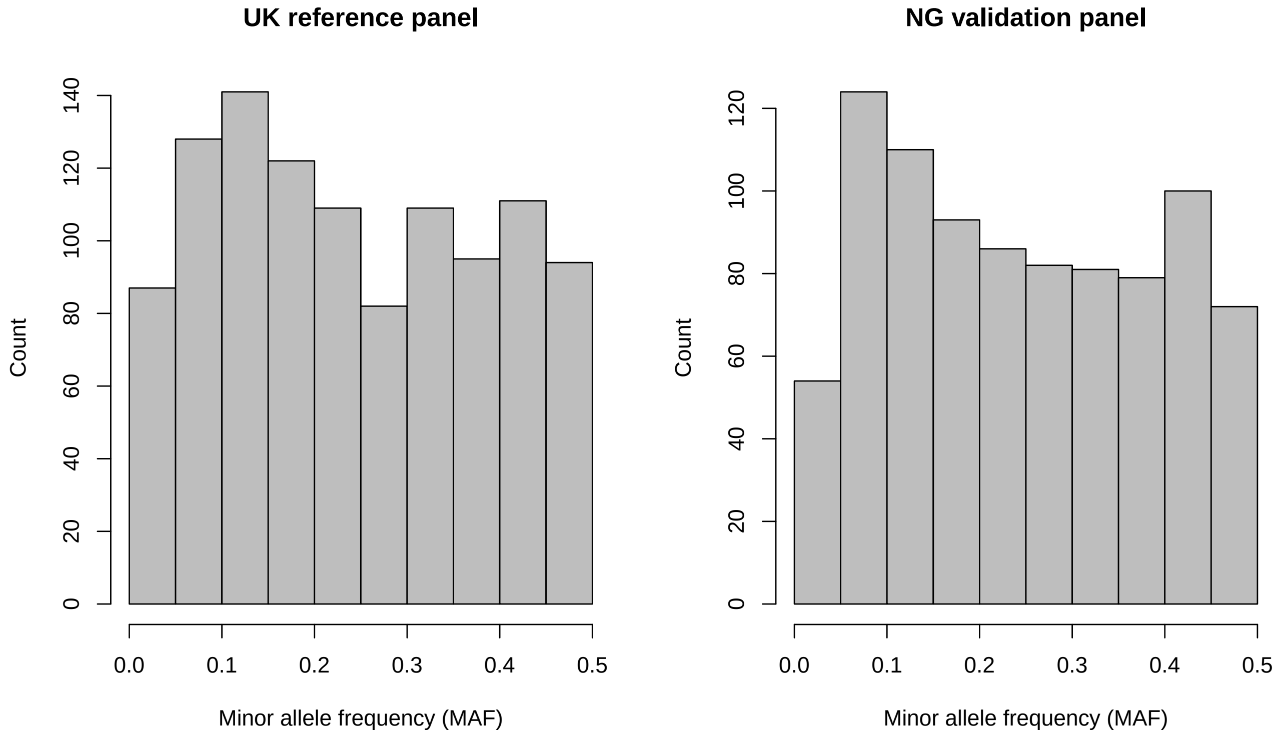

Figure S1. MAF histograms.
The distribution of SNP allele frequencies in each of the reference panels.

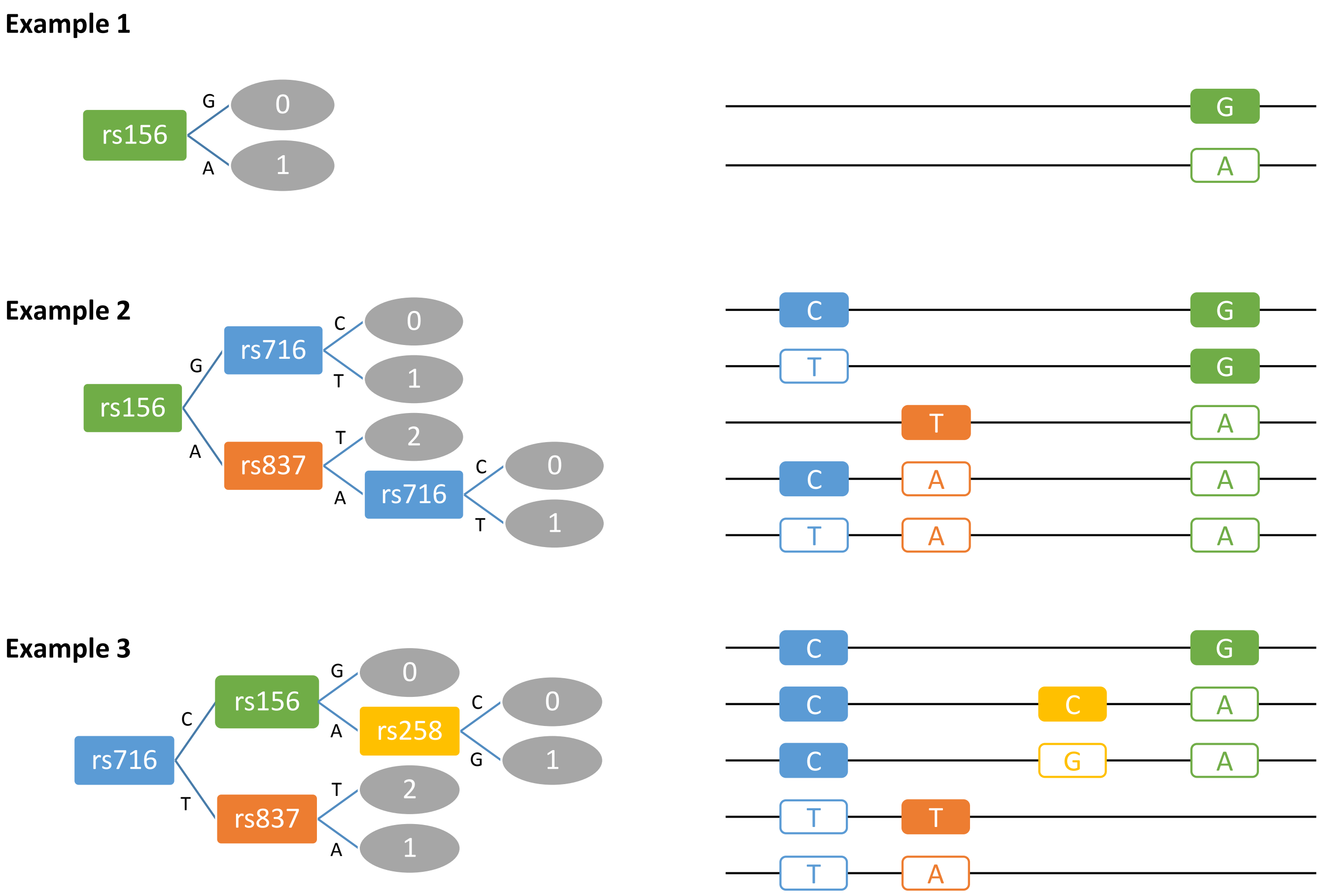

Figure S2. Genetic interpretation of classification trees.
A diagram showing how classification trees describe haplotype partitions. Three example trees are shown on the left and their corresponding haplotype partitions on the right. The coloured rectangular nodes in the trees represent SNPs and the branches leading from each one correspond to alleles at the SNP (as marked); the grey end nodes represent copy number types for the target locus. A path through a tree (from left to right) defines a haplotype, as a set of alleles at a set of SNPs (the ones that appear on that particular path). The haplotype diagrams show all of these haplotypes (one for each end node of each tree, i.e. for each possible path). Each SNP is depicted as a rectangle labelled with its allele, or is absent if that SNP is not on the path defining that haplotype. Note that a given SNP can appear more than once in the same tree (e.g. rs716 in Example 2). By construction, the set of haplotypes from a given tree forms a complete partition of the space of haplotypes.

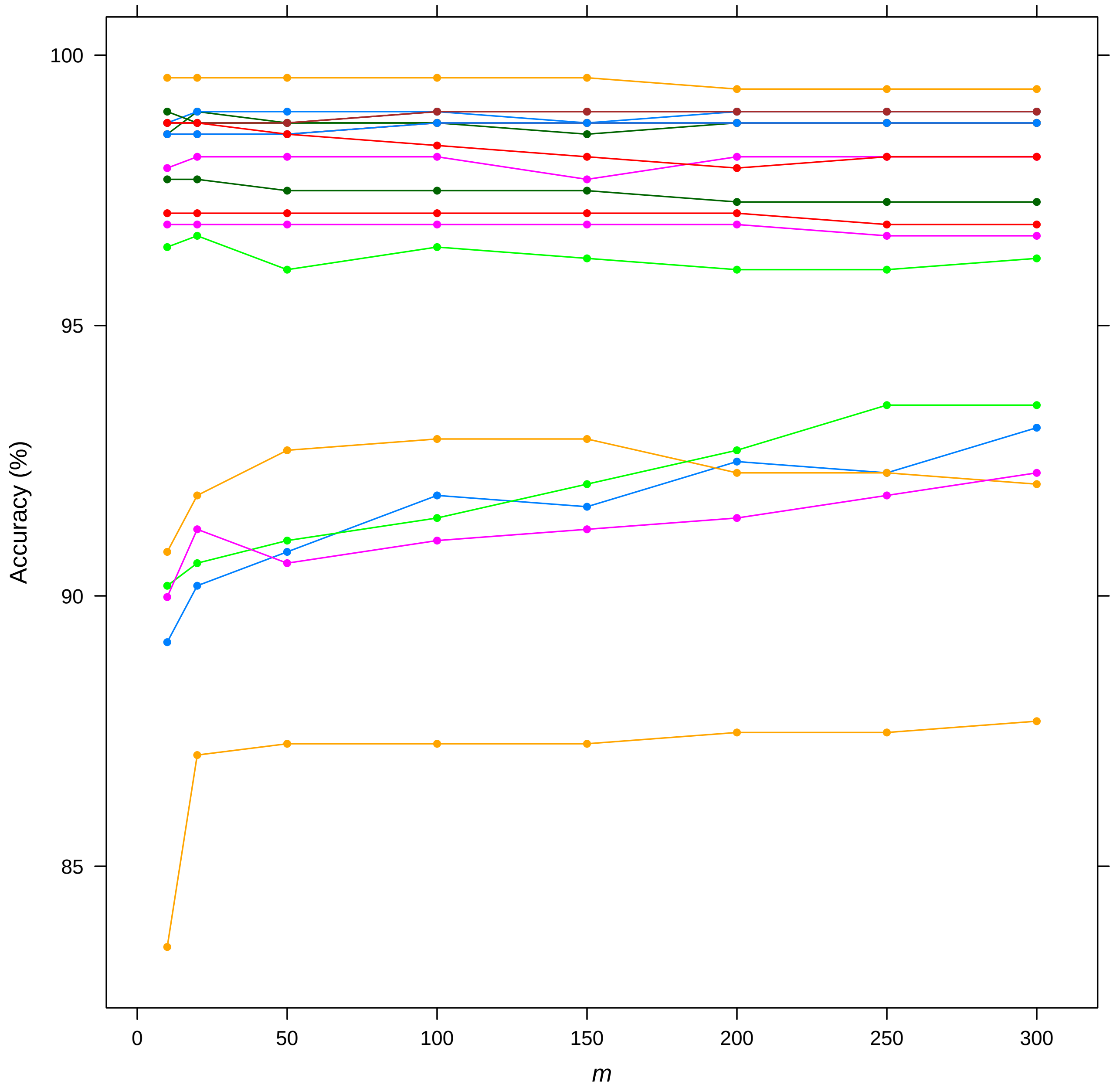

Figure S3. Parameter tuning.
Imputation accuracy estimates from the parameter tuning experiments. Each line shows the OOB accuracy for KIR*IMP for a particular KIR locus, trained with the UK reference panel with the *UKsnps* set, for various values of the key tuning parameter (m). The lines are coloured to distinguish them for visual convenience.

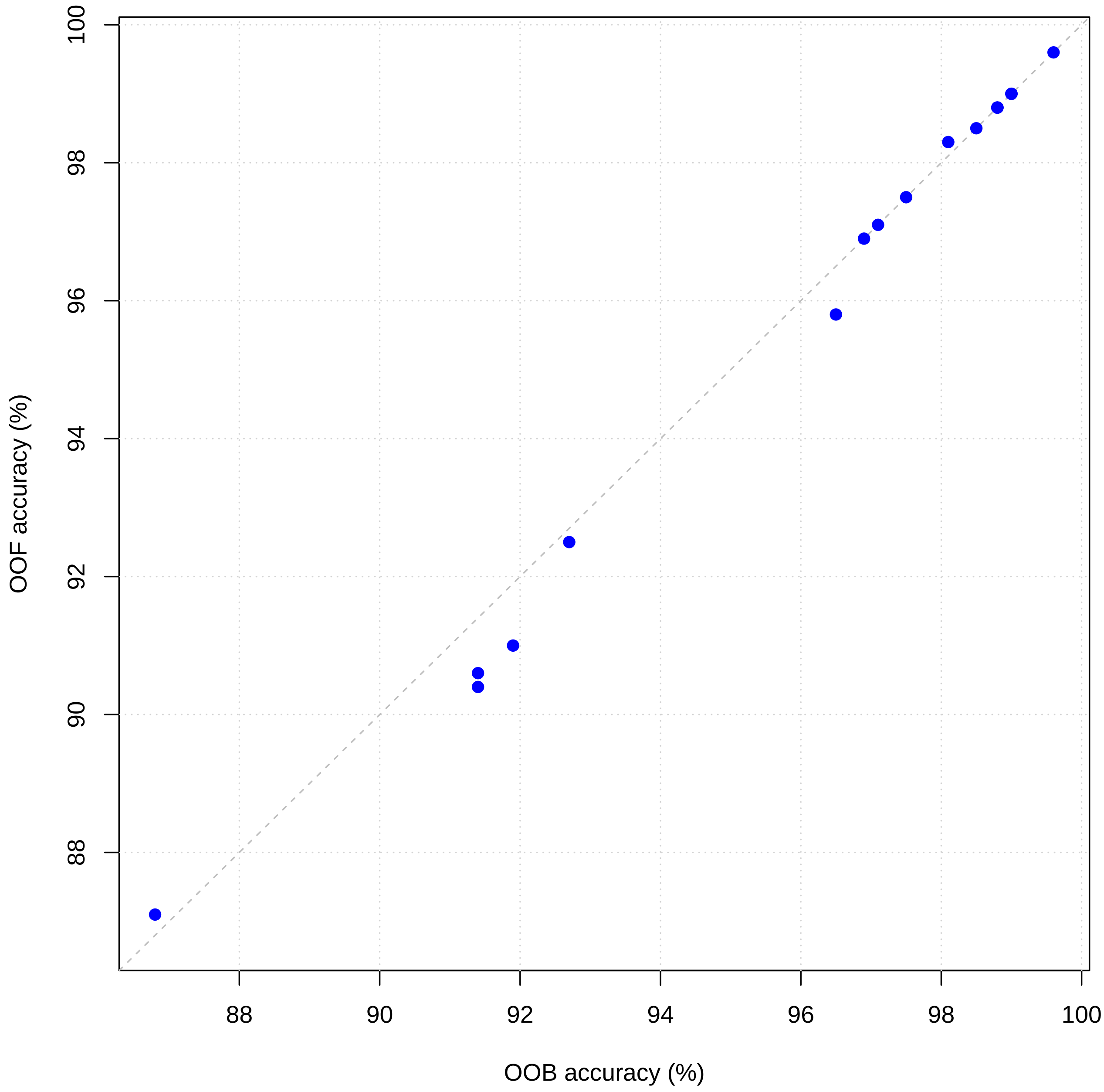

Figure S4. OOB vs cross-validation imputation accuracy.
A comparison of the out-of-bag (OOB) and the out-of-fold (OOF) estimates of imputation accuracy. The former were calculated during training with the full data, the latter as part of the UK cross-validation analysis. Each point corresponds to a different KIR locus. All results are for KIR*IMP trained with the UK reference panel with the *UKsnps* set.

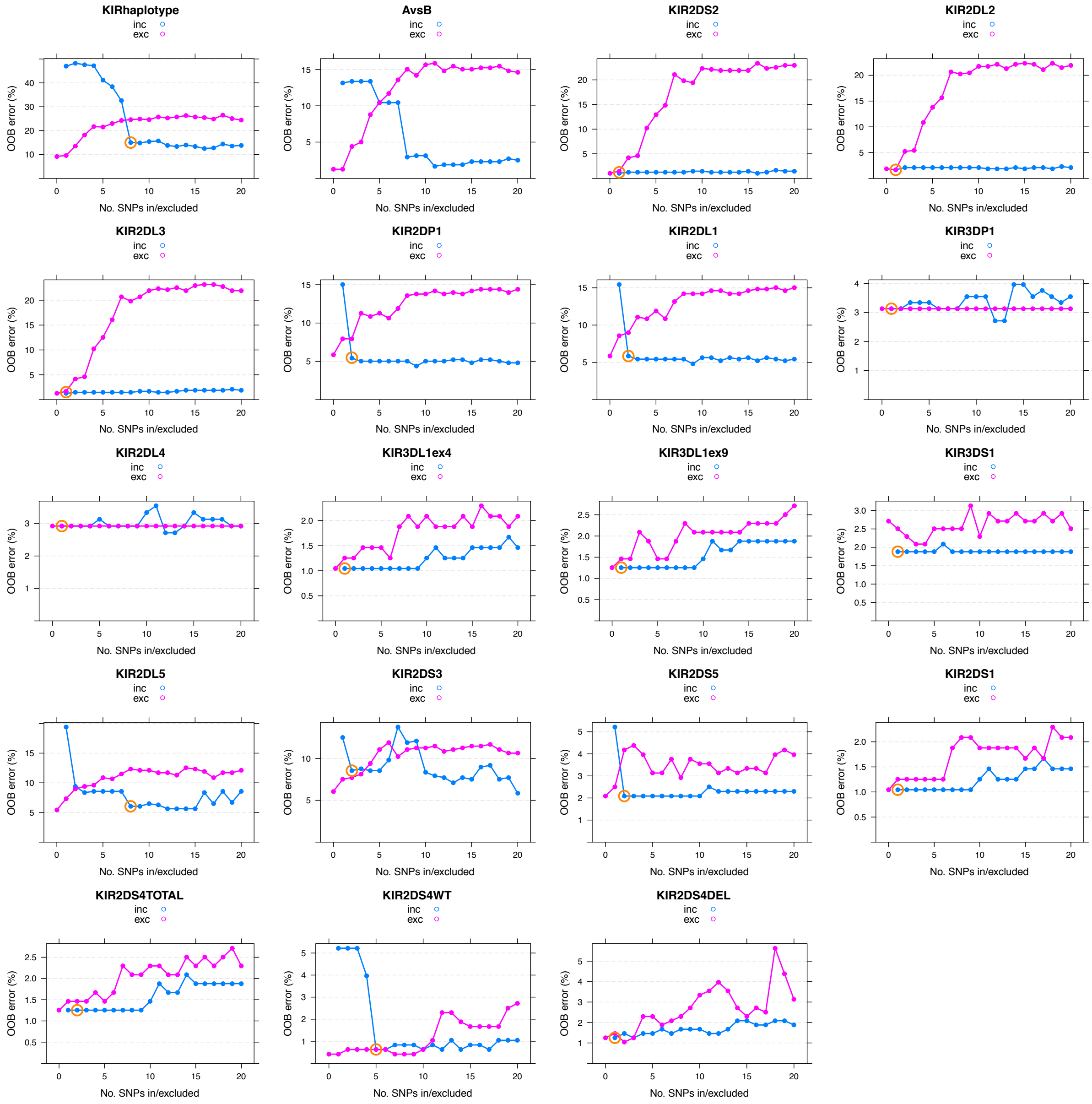

Figure S5. SNP selection experiments.
OOB imputation accuracy estimates from the SNP selection experiments. Each panel shows the results from a different locus, for models fitted with either just the selected SNPs (inc) or all of the remaining SNPs (exc). Points circled in orange indicate which SNPs were manually selected (from that locus) into the set of most informative SNPs (see Material and Methods and Table 2). All results are for KIR*IMP trained with the UK reference panel, starting with the *UKplus4snps* set of SNPs. Note that the y-axis scale varies for each panel.

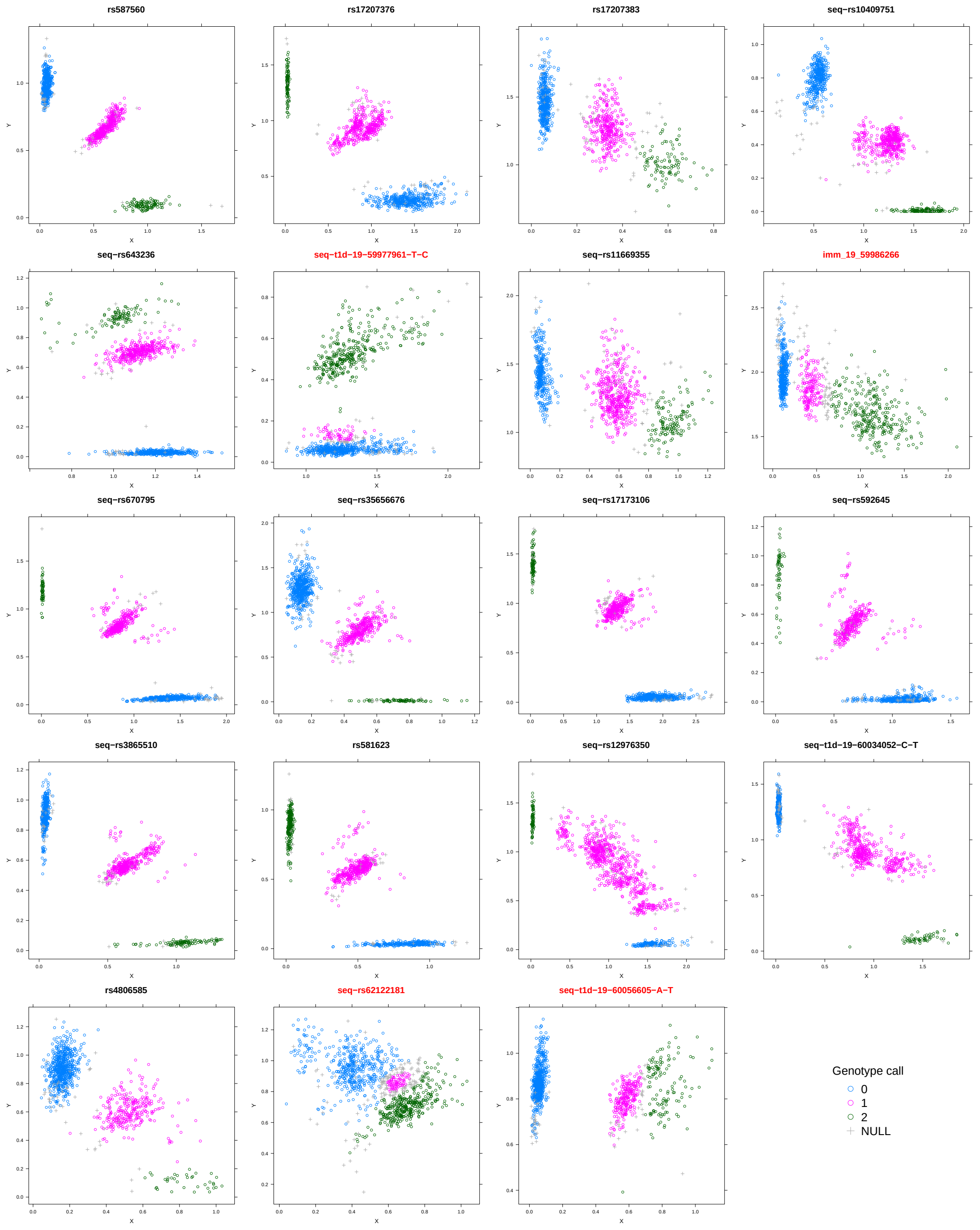

Figure S6. SNP genotyping diagnostic plots.
Scatter plots of the normalised SNP intensity measurements. Each point represents the measurements for a single individual for a given SNP. The shape and colour of each point correspond to the genotype call, as indicated by the legend in the bottom-right. Genotype calls are shown as an integer, with 1 being the heterozygote, and with NULL representing a missing call (i.e. did not meet the relevant quality criteria to be given a call). Each panel shows one of the 19 SNPs identified from the SNP selection experiments (identified by the title of each panel). Four of these were excluded due to poor clustering, these are indicated in Table 2 and shown here by a red title for the panel.

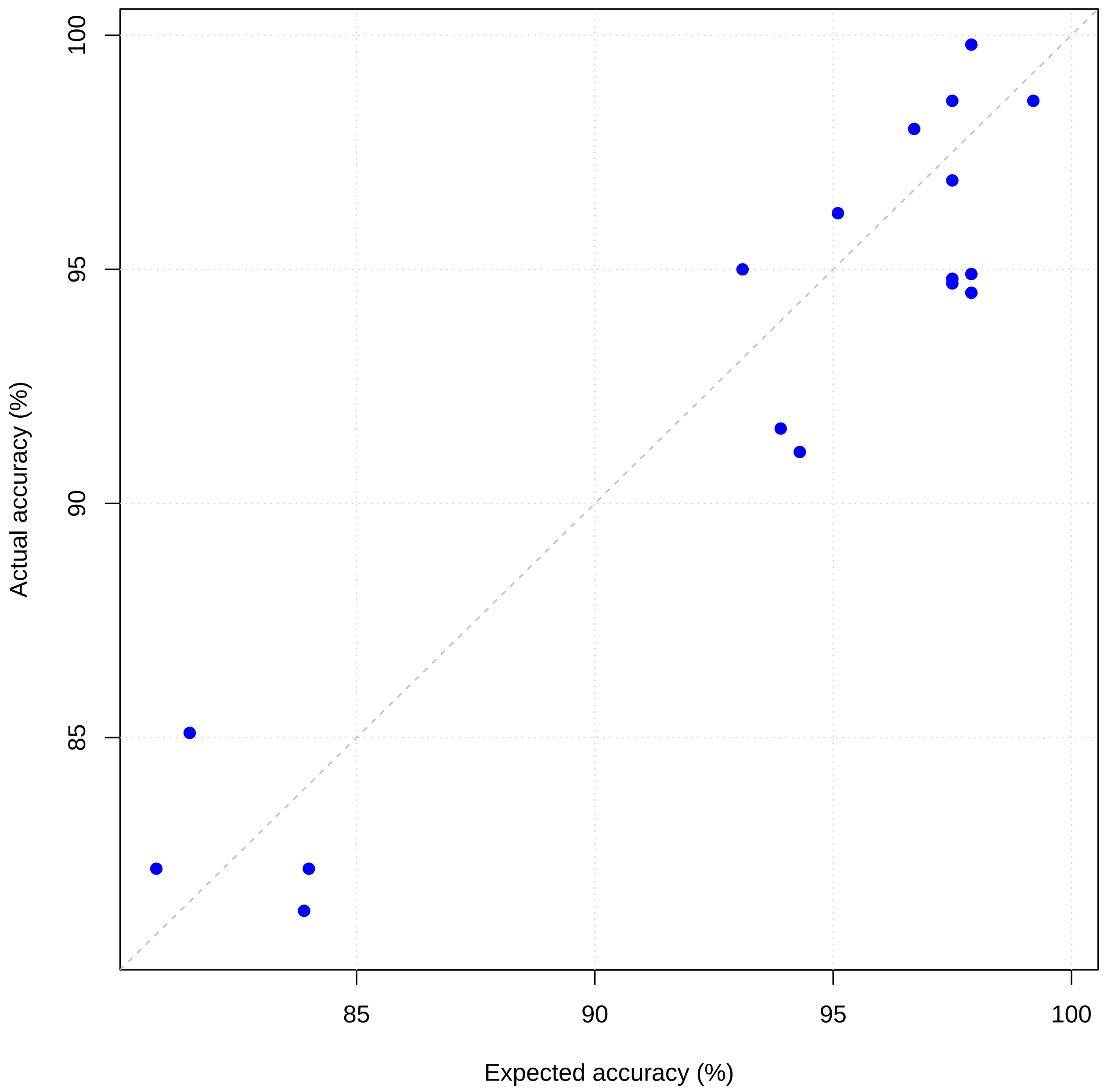

Figure S7. Per-haplotype vs per-individual imputation accuracy.
A comparison of the expected and actual imputation accuracy estimates for the NG validation analysis for KIR*IMP. The actual accuracy estimate is the percentage of individuals with correctly imputed copy number in the NG validation panel. The expected accuracy is calculated from the per-haplotype OOB accuracy estimates on the UK reference panel (obtained during model training), by assuming a model where a pair of haplotypes are chosen independently and uniformly at random and combined. Each point corresponds to a different copy-number KIR locus.

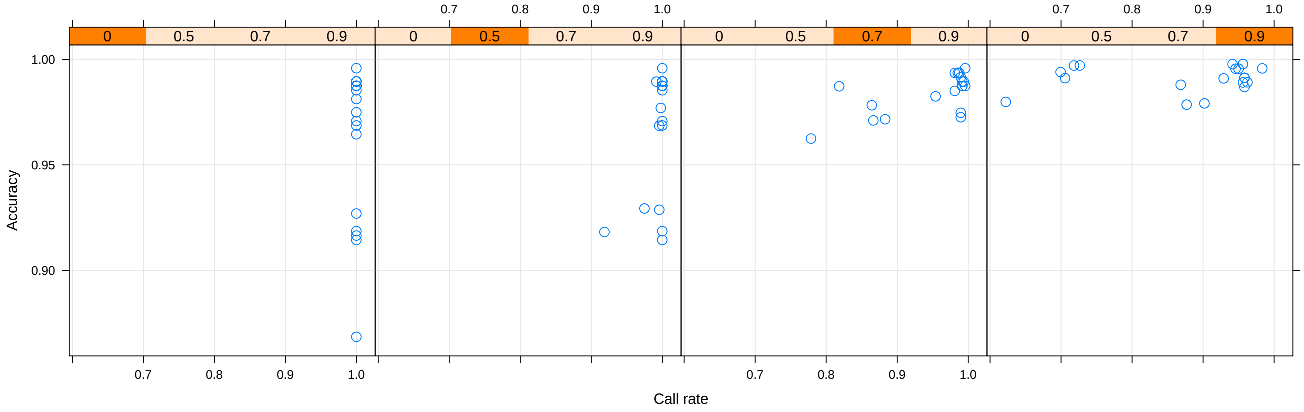

Figure S8. Imputation performance at various calling thresholds.
Scatter plots of OOB imputation accuracy against call rate. Each point corresponds to a different KIR locus and each panel to a different call threshold (as highlighted). All results are for KIR*IMP trained with the UK reference panel with the *UKsnps* set.

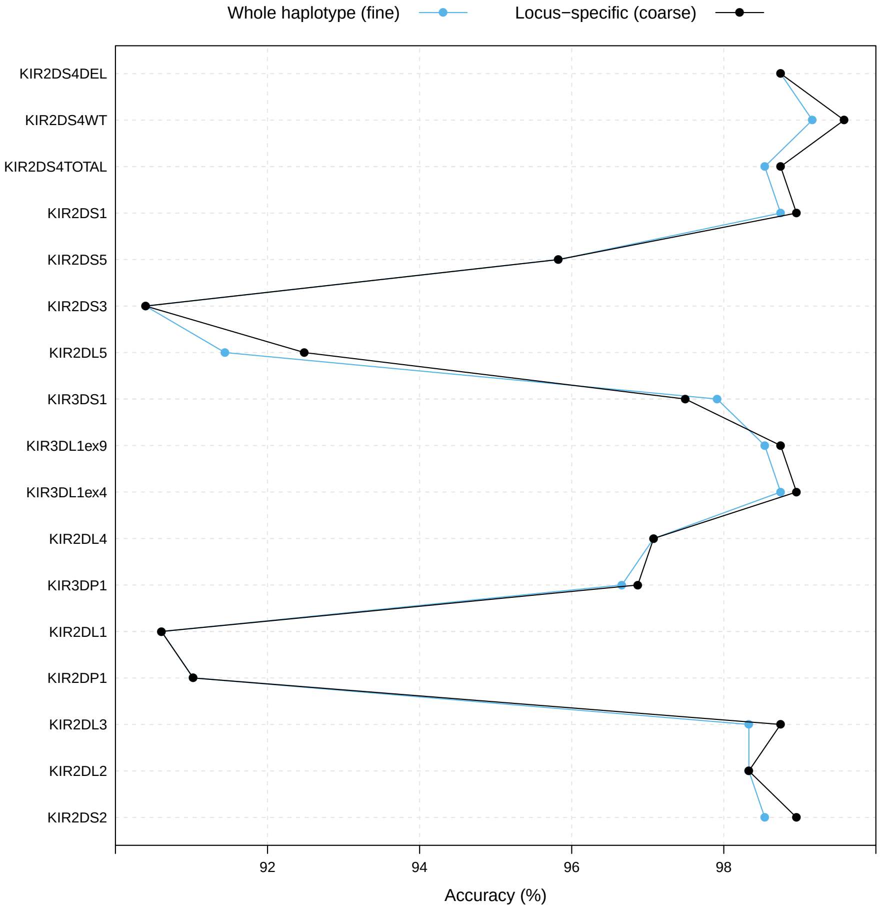

Figure S9. Imputation of fine-grained vs coarse-grained KIR types.
Imputation accuracy for models at different levels of granularity. A single model of the complete, fine-grained KIR haplotypes (coarsened to get calls for copy number for each constituent locus) is compared against separate (coarse-grained) locus-specific models. All results are from the UK cross-validation analysis of KIR*IMP trained with the *UKsnps* set.

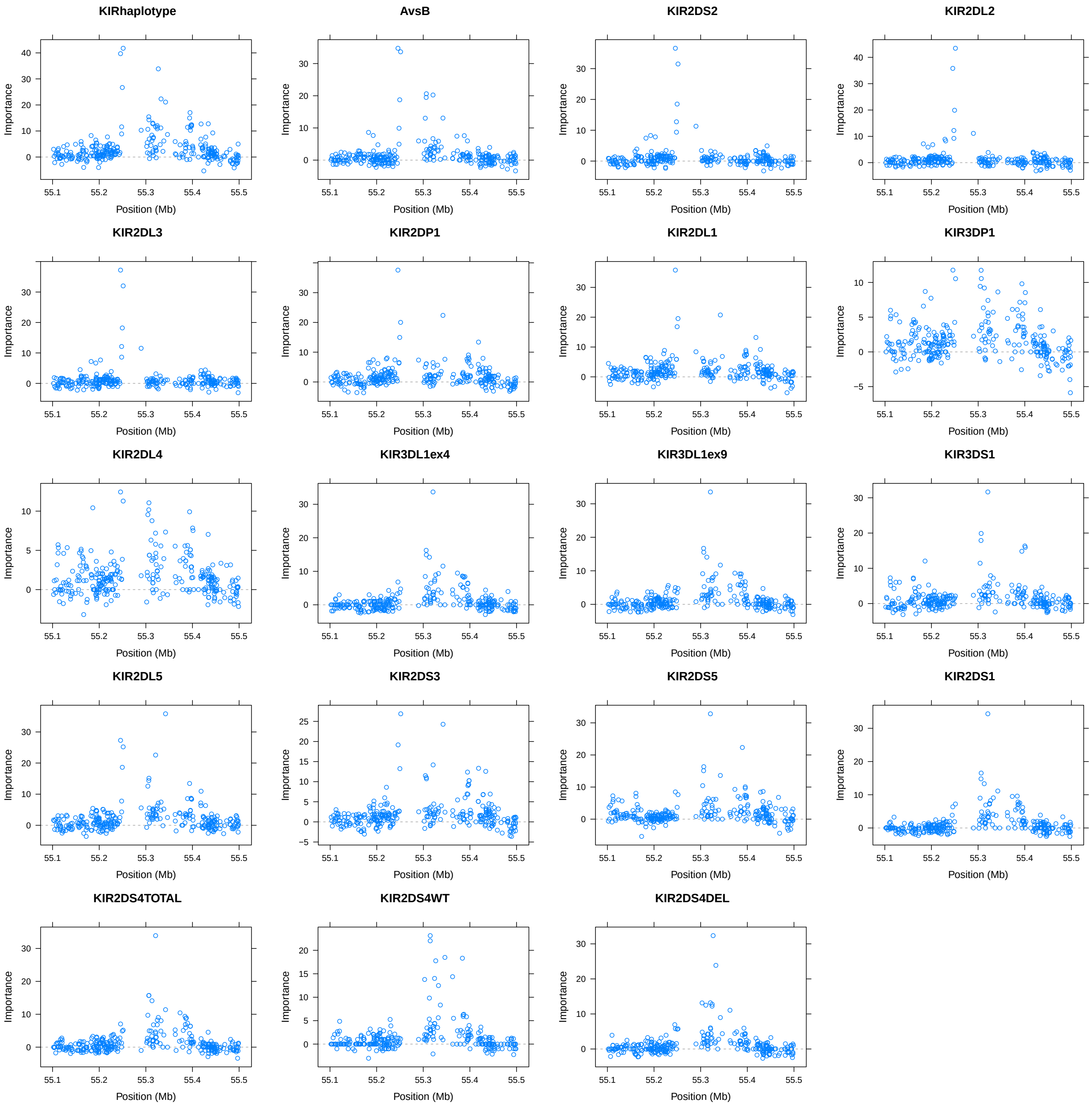

Figure S10. Variable importance plots.
Scatter plots of the variable importance score against genomic position for each SNP (see Material and Methods). Each panel shows the results from a different locus, for KIR*IMP trained with the UK reference panel with the *UKsnps* set.

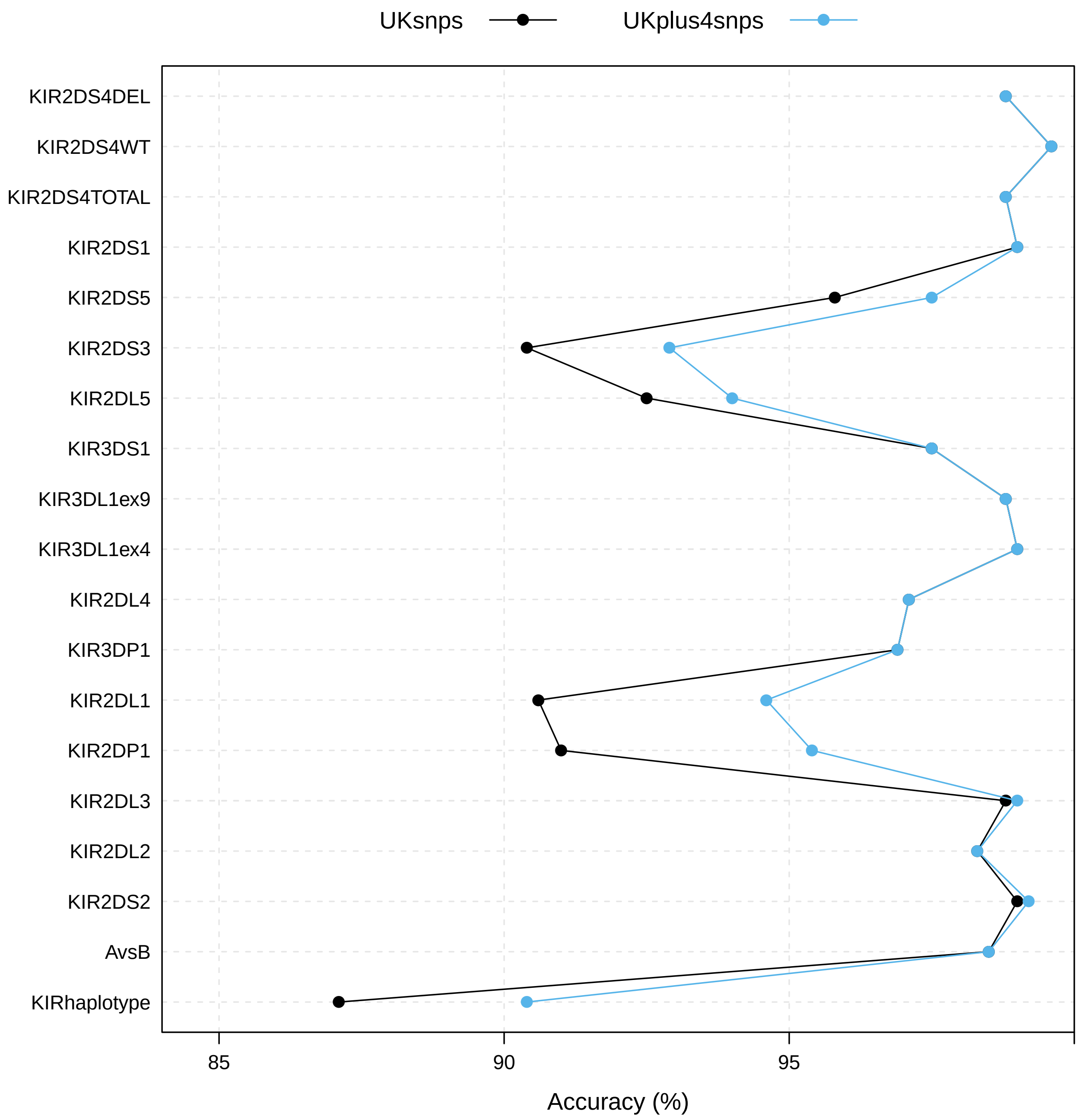

Figure S11. Imputation accuracy when including SNPs with poor clustering. Estimates of the KIR*IMP imputation accuracy from the UK cross-validation analysis, comparing two different SNP subsets for training: the main set of SNPs used for the cross-validation analyses (*UKsnps*) and the same set supplemented with the 4 SNPs identified as having poor clustering (*UKplus4snps*).

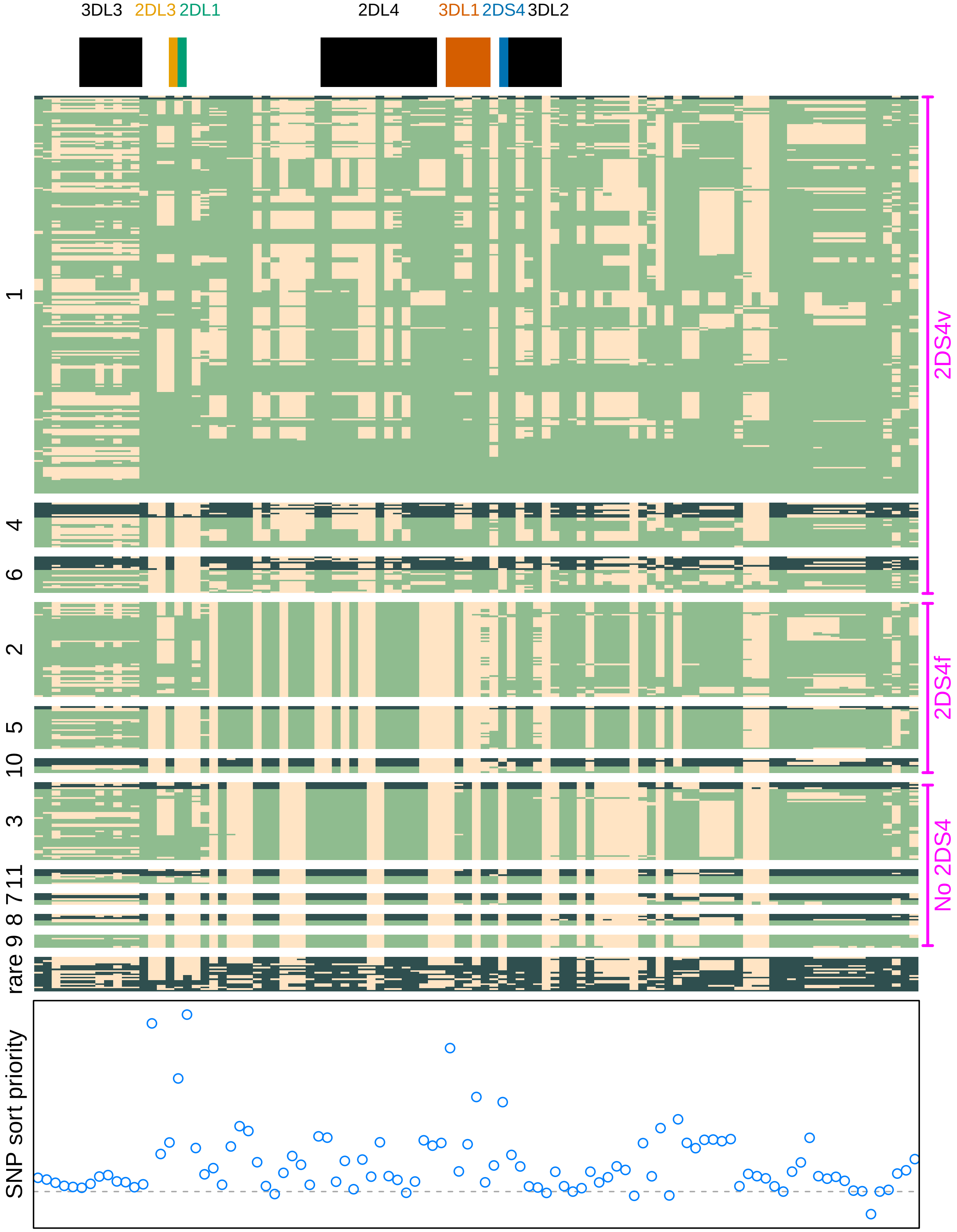

Figure S12. SNP haplotypes. (Continued on the following page)

Figure S12. SNP haplotypes. (cont'd)
A visualisation of the UK reference panel in the style of a heat map. All SNPs in the region 55.23–55.43 Mb are shown, excluding those that were monomorphic in the UK reference panel or had poor clustering. Rows correspond to haplotypes and columns to SNPs.Each cell is coloured either yellow or green (the latter can be either light or dark green, see below) depending on the allele at that SNP for that haplotype; these two colours are mapped arbitrarily to the two alleles at each SNP. Haplotypes are grouped by their KIR haplotype, as labelled on the left; the 11 most common KIR haplotypes are shown separately and the rest are grouped together ('rare'). Within each group, haplotypes are sorted in a lexicographical order based on the SNP alleles, taken in the order defined by their variable importance score from the KIR*IMP model fitted to *KIRhaplotype*; this score is plotted in the panel on the bottom. The haplotype groups are shown in a manually selected order to highlight common patterns between them, with the labels on the right indicating the presence or absence of particular versions of *KIR2DS4* (see Appendix B). SNPs are shown in annotated genomic position order (n.b. they are therefore not drawn to scale based on genomic position, like in Figure 1). Two shades of green are used: light green for haplotypes that are imputed correctly, dark green for those imputed incorrectly. The coloured bars at the top indicate SNPs that are annotated as being within a KIR gene, using the same colour scheme as Figure 1.

Supplemental Tables

Table S1. KIR loci.

Gene			
**Current knowledge**	**HGNC**	**Assay identifier**	**MIM**
KIR3DL3	*KIR3DL3*	*KIR3DL3*	610095
KIR2DS2	*KIR2DS2*	*KIR2DS2*	604953
*KIR2DL2/L3**	*KIR2DL2*	*KIR2DL2*	604937
	KIR2DL3	*KIR2DL3*	604938
KIR2DP1	*KIR2DP1*	*KIR2DP1*	—
KIR2DL1	*KIR2DL1*	*KIR2DL1*	604936
KIR3DP1	*KIR3DP1*	*KIR3DP1*	610604
KIR2DL4	*KIR2DL4*	*KIR2DL4*	604945
*KIR3DL1/S1**	*KIR3DL1*	*KIR3DL1ex4*	604946
		KIR3DL1ex9	
	KIR3DS1	*KIR3DS1*	
KIR2DL5A	*KIR2DL5A*	*KIR2DL5*	605305
KIR2DL5B	*KIR2DL5B*		615727
KIR2DS3	*KIR2DS3*	*KIR2DS3*	604954
KIR2DS5	*KIR2DS5*	*KIR2DS5*	604956
KIR2DS1	*KIR2DS1*	*KIR2DS1*	604952
KIR2DS4	*KIR2DS4*	*KIR2DS4TOTAL*	604955
		KIR2DS4WT	
		KIR2DS4DEL	
KIR3DL2	*KIR3DL2*	*KIR3DL2*	604947

The relationship between KIR genes (and pseudogenes) and our copy number assays. We show both the set of genes defined by the HUGO Genome Nomenclature Committee (HGNC) and also a revised set ('Current knowledge') where two pairs of genes have been merged (marked by an asterisk) reflecting more recent research showing that these are actually different alleles of the same gene.[1] The MIM number for each gene is also provided; see MIM 604936 for general information about the KIR gene family.

Table S2. Definitions of KIR haplotypes.

KIRhaplotype	AvsB	KIR3DL3	KIR2DS2	KIR2DL2	KIR2DL3	KIR2DP1	KIR2DL1	KIR3DP1	KIR2DL4	KIR3DL1ex4	KIR3DL1ex9	KIR3DS1	KIR2DL5	KIR2DS3	KIR2DS5	KIR2DS1	KIR2DS4TOTAL	KIR2DS4WT	KIR2DS4DEL	KIR3DL2
1	A	1	0	0	1	1	1	1	1	1	1	0	0	0	0	0	1	0	1	1
2	A	1	0	0	1	1	1	1	1	1	1	0	0	0	0	0	1	1	0	1
3	B	1	0	0	1	1	1	1	1	0	0	1	1	0	1	1	0	0	0	1
4	B	1	1	1	0	0	0	1	1	1	1	0	0	0	0	0	1	0	1	1
5	B	1	1	1	0	0	0	1	1	1	1	0	0	0	0	0	1	1	0	1
6	B	1	1	1	0	1	1	1	1	1	1	0	1	1	0	0	1	0	1	1
7	B	1	1	1	0	1	1	1	1	0	0	1	2	1	1	1	0	0	0	1
8	B	1	1	1	0	0	0	1	1	0	0	1	1	0	1	1	0	0	0	1
9	B	1	1	1	0	1	1	1	1	0	0	1	2	2	0	1	0	0	0	1
10	B	1	1	1	0	1	1	1	1	1	1	0	1	1	0	0	1	1	0	1
11	B	1	0	0	1	1	1	1	1	0	0	1	1	1	0	1	0	0	0	1
12	B	1	1	1	0	0	0	0	0	0	0	0	1	0	1	1	0	0	0	1
13	B	1	1	1	0	1	1	2	2	1	1	1	1	1	0	0	1	1	0	1
14	B	1	0	0	1	2	2	2	2	0	0	2	2	1	1	1	0	0	0	1
15	B	1	1	0	0	1	1	1	1	0	0	1	1	0	1	1	0	0	0	1
16	B	1	0	0	1	1	1	2	2	1	1	1	0	0	0	0	1	0	1	1
17	B	1	1	1	0	0	0	1	1	0	0	1	1	1	0	1	0	0	0	1
18	B	1	1	1	0	0	0	0	0	0	0	0	1	1	0	1	0	0	0	1
19	B	1	1	2	0	0	0	2	2	0	0	2	2	0	2	1	0	0	0	1
20	B	1	0	0	1	1	1	2	2	1	1	1	0	0	0	0	1	1	0	1
21	B	1	1	0	0	1	1	1	1	1	1	0	0	0	0	0	1	0	1	1
22	B	1	0	0	1	1	0	0	0	0	0	0	0	0	0	1	0	0	0	0
23	B	1	0	0	1	2	2	2	2	1	1	1	1	1	0	0	1	0	1	1
24	B	1	1	1	0	1	1	1	1	0	0	1	1	1	1	1	0	0	0	1
25	B	1	1	1	0	1	1	1	1	1	1	0	1	0	0	0	1	0	1	1
27	A	1	0	0	1	1	0	1	1	1	1	0	0	0	0	0	1	0	1	1
28	B	1	0	0	1	1	1	2	2	0	0	2	1	0	1	1	0	0	0	1
29	B	1	1	1	0	1	1	2	2	1	1	1	1	1	0	0	1	0	1	1
30	B	1	1	1	0	0	0	2	2	1	1	1	0	0	0	0	1	0	1	1
31	B	1	0	1	0	1	1	1	1	1	1	0	1	0	1	0	1	1	0	1
33	B	1	0	0	1	1	0	0	0	0	0	0	0	0	0	1	0	0	0	1
34	A	1	0	0	1	0	1	0	0	0	0	0	0	0	0	0	0	0	0	1
36	B	1	1	1	0	2	2	2	2	0	0	2	1	2	0	1	0	0	0	1
38	B	1	0	0	1	2	2	2	2	0	0	2	1	0	1	1	0	0	0	1
40	B	1	1	1	0	2	2	1	1	0	0	1	2	1	1	0	0	0	0	1
41	B	1	1	1	0	1	2	1	1	0	0	1	2	2	0	1	0	0	0	1

Continued on the following page

Table S2. Definitions of KIR haplotypes.

KIRhaplotype	AvsB	KIR3DL3	KIR2DS2	KIR2DL2	KIR2DL3	KIR2DP1	KIR2DL1	KIR3DP1	KIR2DL4	KIR3DL1ex4	KIR3DL1ex9	KIR3DS1	KIR2DL5	KIR2DS3	KIR2DS5	KIR2DS1	KIR2DS4TOTAL	KIR2DS4WT	KIR2DS4DEL	KIR3DL2
42	B	1	1	2	0	0	0	2	2	1	1	1	1	0	1	0	1	0	1	1
44	B	1	1	1	0	1	1	1	1	0	0	1	2	0	1	1	0	0	0	1
45	B	1	1	1	0	1	1	1	1	0	0	1	2	1	1	0	0	0	0	1
46	B	1	0	1	0	1	1	1	1	0	0	1	1	1	1	1	0	0	0	1
48	A	1	0	0	1	1	1	1	1	1	2	0	0	0	0	0	2	2	0	1
50	B	1	0	0	0	1	1	1	1	1	1	0	1	1	0	0	1	1	0	1
52	B	1	0	0	0	1	1	1	1	1	1	0	1	0	1	0	1	0	1	1
53	B	1	1	0	1	1	1	1	1	1	1	0	0	0	0	0	1	0	1	1
55	A	1	0	0	0	1	2	1	1	1	1	0	0	0	0	0	1	1	0	1
56	B	1	1	1	0	0	0	0	1	0	0	1	1	0	1	1	0	0	0	1
57	A	1	0	0	1	1	1	0	1	1	1	0	0	0	0	0	1	0	1	1
58	A	1	0	0	1	1	1	1	0	1	1	0	0	0	0	0	1	1	0	1
59	A	1	0	0	1	1	1	1	1	1	0	0	0	0	0	0	0	0	0	1
68	B	1	1	1	0	0	0	0	1	1	1	0	0	0	0	0	1	0	1	1
69	B	1	1	1	0	0	0	0	0	0	0	0	1	0	0	1	0	0	0	1

The relationship between the different KIR types in our data. Each column represents a KIR type: *KIRhaplotype* is the haplotype classification defined by Jiang *et al.*[2], *AvsB* corresponds to the broad A/B haplotype group classification, and the other columns are the copy number of individual KIR genes. Each *KIRhaplotype* is uniquely defined by the copy number values across the 17 KIR loci.

Table S3. KIR allele frequencies.

(A) UK fine-scale haplotypes

KIRhaplotype	**Count**
1	239
2	57
3	47
4	27
5	26
6	22
7	7
8	7
9	8
10	9
11	9
12	4
13	1
14	2
16	1
18	2
19	1
20	1
21	1
25	1
27	1
42	1
44	1
48	1
55	1
56	1
69	1

(B) UK A/B haplotypes

AvsB	**Count**
A	299
B	180

(C) UK copy number types

**Gene**	**0**	**1**	**2**
KIR3DL3	0	479	0
KIR2DS2	359	120	0
KIR2DL2	360	117	2
KIR2DL3	121	358	0
KIR2DP1	70	407	2
KIR2DL1	71	405	3
KIR3DP1	8	464	7
KIR2DL4	7	465	7
KIR3DL1ex4	90	389	0
KIR3DL1ex9	90	388	1
KIR3DS1	392	84	3
KIR2DL5	355	105	19
KIR2DS3	419	52	8
KIR2DS5	408	70	1
KIR2DS1	389	90	0
KIR2DS4TOTAL	90	388	1
KIR2DS4WT	383	95	1
KIR2DS4DEL	186	293	0
KIR3DL2	0	479	0

(D) NG copy number types

**Gene**	**0**	**1**	**2**	**3**	**4**	**Null**
KIR3DL3	0	1	1330	7	0	0
KIR2DS2	702	526	108	0	0	2
KIR2DL2	710	510	113	4	0	1
KIR2DL3	111	524	697	4	0	2
KIR2DP1	42	346	933	15	1	1
KIR2DL1	46	383	894	14	1	0
KIR3DP1	1	55	1213	57	3	9
KIR2DL4	1	58	1210	59	3	7
KIR3DL1ex4	62	439	831	6	0	0
KIR3DL1ex9	62	441	835	0	0	0
KIR3DS1	856	403	72	6	0	1
KIR2DL5	724	404	180	27	3	0
KIR2DS3	1003	267	63	5	0	0
KIR2DS5	941	349	45	3	0	0
KIR2DS1	839	437	61	1	0	0
KIR2DS4TOTAL	62	441	835	0	0	0
KIR2DS4WT	814	463	61	0	0	0
KIR2DS4DEL	244	662	432	0	0	0
KIR3DL2	1	38	1299	0	0	0

Sample counts for the KIR types in the reference panels. For the UK reference panel: (**A**) fine-scale KIR haplotypes (*KIRhaplotype*); (**B**) A/B haplotypes (*AvsB*); (**C**) copy number, per-haplotype. For the NG validation panel: (**D**) copy number, per-individual. For the copy number sub-tables (**C**–**D**), each row represents a KIR locus, each column a particular copy number, with the sample counts in each cell. The 'Null' column shows the number of individuals with missing data for each locus.

Table S4. SNP tagging.

Locus	Tag SNP	LD (r^2)	Accuracy (%)
AvsB	rs587560	0.53	86.8
KIR2DS2	rs587560	0.95	99.0
KIR2DL2	rs587560	0.92	98.3
KIR2DL3	rs587560	0.93	98.8
KIR2DP1	rs587560	0.47	88.5
KIR2DL1	rs587560	0.45	88.1
KIR3DP1	seq-rs1010355	0.00	96.9
KIR2DL4	seq-rs1010355	0.00	97.1
KIR3DL1ex4	seq-rs592645	0.93	99.0
KIR3DL1ex9	seq-rs592645	0.92	98.8
KIR3DS1	seq-rs592645	0.89	98.1
KIR2DL5	seq-t1d-19-60034052-C-T	0.67	90.6
KIR2DS3	seq-rs1010355	0.00	87.5
KIR2DS5	seq-rs592645	0.67	94.8
KIR2DS1	seq-rs592645	0.93	99.0
KIR2DS4TOTAL	seq-rs592645	0.92	98.8
KIR2DS4WT	rs4806585	0.73	96.0
KIR2DS4DEL	rs581623	0.95	98.8

The best tag SNP for each copy number locus and *AvsB*. The SNPs were selected to maximise prediction accuracy (see Material and Methods), which is shown in the final column. Also reported is the square of the Pearson correlation coefficient, a measure of the linkage disequilibrium (LD) with the target locus. Note that the accuracy estimates shown here can differ slightly to those shown in the methods comparison (see Material and Methods).

Table S5. KIR imputation with other SNP arrays.

Manufacturer	Array	SNPs in ***UKsnps***	KIRhaplotype	AvsB	KIR2DS2	KIR2DL2	KIR2DL3	KIR2DP1	KIR2DL1	KIR3DP1
Illumina	**Immunochip**	**301**	**86.8**	**98.5**	**99.0**	**98.1**	**98.8**	**91.9**	**91.4**	**96.9**
	Human1M-Duo v3.0	109	84.5	97.1	98.8	98.1	98.5	90.4	91.0	96.9
	Human610-Quad v1.0	70	79.5	94.2	95.8	95.0	95.6	88.3	87.9	96.9
	Human660W-Quad v1.0	68	79.8	94.4	95.8	95.2	95.4	88.7	87.7	96.9
	HumanCore-12 v1.0	26	72.2	78.3	74.5	74.5	74.3	85.0	84.8	96.7
	HumanCore-24 v1.0	26	64.5	71.6	74.5	75.4	74.7	84.8	84.8	96.9
	HumanCoreExome-24 v1.0	30	64.5	72.4	74.3	73.9	72.4	84.5	84.1	96.9
	HumanOmni1S-8 v1.0	54	73.7	86.4	77.5	77.9	77.9	86.8	86.4	96.9
	HumanOmni2.5-Quad	145	86.6	98.5	99.0	98.3	98.8	91.7	92.3	96.9
	HumanOmni2-5-8 v1.2	134	83.5	98.5	99.0	98.3	98.8	91.2	91.7	97.1
	HumanOmni2.5S-8 v1.0	58	73.3	92.3	95.6	94.8	95.4	90.2	90.0	96.2
	HumanOmni5-4 v1.1	164	83.1	98.5	99.0	98.3	98.8	91.9	91.7	96.9
	HumanOmniExpress-24 v1.1	77	80.2	94.0	99.0	98.3	98.8	90.8	90.2	96.9
	HumanOmniExpressExome-8 v1.2	82	85.8	98.3	99.0	98.3	98.8	91.0	90.4	96.9
	HumanOmniExpressExome-8 v1.0	78	86.2	98.5	99.0	98.1	98.8	91.4	91.7	96.9
Affymetrix	Genome-Wide Human 5.0	23	52.4	71.2	77.0	76.6	77.5	86.0	85.0	96.7
	Genome-Wide Human 6.0	52	66.6	85.0	80.6	81.0	81.2	86.4	85.4	96.7
	GeneChip Human Mapping 10K 2.0	1	49.9	62.4	75.0	75.2	74.7	85.0	84.5	96.9
	GeneChip Human Mapping 100K Set	7	49.9	62.4	75.0	75.2	74.7	85.0	84.5	96.9
	GeneChip Human Mapping 500K Set	26	62.0	81.6	77.2	78.9	78.7	86.8	86.2	96.2
	Axiom Biobank	34	60.8	76.6	74.5	75.0	74.3	85.0	85.0	96.7
	Axiom Exome 319	10	56.0	77.5	74.7	75.0	74.5	84.8	84.3	96.7
	Axiom Genome-Wide EUR 1	52	74.3	85.4	79.1	79.1	78.7	86.2	86.2	96.7
	UK Biobank Axiom	241	81.6	95.8	95.8	95.0	95.6	89.8	88.3	96.9

Continued on the following page

Table S5. KIR imputation with other SNP arrays.

Manufacturer	Array	KIR2DL4	KIR3DL1ex4	KIR3DL1ex9	KIR3DS1	KIR2DL5	KIR2DS3	KIR2DS5	KIR2DS1	KIR2DS4TOTAL	KIR2DS4WT	KIR2DS4DEL
Illumina	**Immunochip**	**97.1**	**99.0**	**98.8**	**97.5**	**92.7**	**91.4**	**96.5**	**99.0**	**98.8**	**99.6**	**98.8**
	Human1M-Duo v3.0	97.1	97.1	96.9	96.5	88.9	90.6	96.2	97.1	97.1	98.3	98.8
	Human610-Quad v1.0	97.1	97.1	96.7	96.5	88.3	89.6	96.0	96.9	96.7	97.5	98.8
	Human660W-Quad v1.0	97.1	96.9	96.7	96.2	87.9	88.9	95.6	96.9	96.7	97.5	98.8
	HumanCore-12 v1.0	96.9	97.1	96.9	96.0	86.6	88.1	96.2	97.1	97.1	98.1	98.3
	HumanCore-24 v1.0	97.1	88.7	88.3	88.5	80.8	88.3	91.2	88.9	88.1	95.0	85.0
	HumanCoreExome-24 v1.0	97.1	89.3	88.5	89.3	81.4	87.3	91.7	89.3	88.1	95.2	85.4
	HumanOmni1S-8 v1.0	97.1	98.8	98.5	97.7	90.6	88.9	95.0	98.8	98.5	98.5	96.7
	HumanOmni2.5-Quad	97.1	98.3	98.3	97.9	92.9	91.2	96.5	98.5	98.3	99.2	98.8
	HumanOmni2-5-8 v1.2	97.3	98.1	97.9	97.7	92.7	91.0	96.9	98.1	97.9	96.5	97.7
	HumanOmni2.5S-8 v1.0	96.7	94.6	94.4	92.9	88.3	90.6	92.1	94.4	94.2	96.5	93.3
	HumanOmni5-4 v1.1	97.1	98.1	97.9	97.3	92.7	90.8	97.1	97.9	97.9	96.7	98.3
	HumanOmniExpress-24 v1.1	97.1	95.0	94.6	92.1	88.1	90.8	92.5	95.4	94.8	95.8	95.0
	HumanOmniExpressExome-8 v1.2	97.1	97.7	97.7	97.9	91.0	91.4	96.2	97.7	97.7	98.5	98.8
	HumanOmniExpressExome-8 v1.0	97.1	97.9	97.5	98.1	91.2	90.2	96.5	97.9	97.5	98.1	98.8
Affymetrix	Genome-Wide Human 5.0	96.7	79.1	78.9	79.3	72.4	85.6	83.1	79.3	79.1	81.4	80.8
	Genome-Wide Human 6.0	97.1	97.7	97.7	96.5	86.6	88.7	93.5	97.5	97.5	88.5	86.4
	GeneChip Human Mapping 10K 2.0	97.1	81.2	81.0	81.8	74.1	87.5	85.2	81.2	81.0	80.0	61.2
	GeneChip Human Mapping 100K Set	97.1	81.2	81.0	81.8	74.1	87.5	85.2	81.2	81.0	80.0	56.0
	GeneChip Human Mapping 500K Set	96.9	97.3	97.1	96.5	86.2	85.6	93.7	97.5	97.3	86.6	85.2
	Axiom Biobank	96.9	92.7	92.7	92.5	83.9	88.5	92.1	92.7	92.7	84.3	78.1
	Axiom Exome 319	96.9	91.9	92.1	91.9	84.3	87.5	86.2	92.1	92.3	81.2	73.9
	Axiom Genome-Wide EUR 1	96.9	98.3	98.3	97.5	88.1	88.1	95.4	98.5	98.1	98.8	98.8
	UK Biobank Axiom	97.1	99.0	98.5	97.5	91.2	89.8	96.0	98.8	98.8	99.6	98.8

Table description on the following page

Table S5. KIR imputation with other SNP arrays.

Estimates of the best-case imputation accuracy (%) for various SNP genotyping arrays, under the assumption that all SNPs in common with the Immunochip would be typed perfectly in a study sample. The estimates are OOB accuracy from a model fitted with the subset of the *UKsnps* set that are present on each array (the exact number is reported in the 3rd column on the first page of the table). All models are fitted using the UK reference panel, which is typed on the Immunochip; the results for the Immunochip are shown in bold as a reminder of this fact. Given the Immunochip-specific nature of this analysis, these results cannot be interpreted as measuring the absolute potential of each array for studying the KIR region. For this reason, we caution against any comparisons of these results between arrays. Rather, we suggest using this as a guide to the potential of using KIR*IMP with the current reference panel for any existing data typed on a given array.

Supplemental References

[1] Carrington, M. and Norman, P. (2003). The KIR Gene Cluster. (National Center for Biotechnology Information (US)).

[2] Jiang, W., Johnson, C., Jayaraman, J., Simecek, N., Noble, J., Moffatt, M. F., Cookson, W. O., Trowsdale, J., and Traherne, J. A. (2012). Copy number variation leads to considerable diversity for B but not A haplotypes of the human KIR genes encoding NK cell receptors. Genome Research *22*, 1845–1854.
