# Supplementary material for: Imputation of KIR Types from SNP Variation Data
Source: Am J Hum Genet. 2015 Oct 1;97(4):593–607. doi: 10.1016/j.ajhg.2015.09.005 (PMC4596914; doi:10.1016/j.ajhg.2015.09.005)
Supplement: Document S2. Article plus Supplemental Data [file mmc2.pdf]

Imputation of KIR Types from SNP Variation Data

Damjan Vukcevic,[1,2] James A. Traherne,[3,4] Sigrid Næss,[5,6] Eva Ellinghaus,[7] Yoichiro Kamatani,[8,9] Alexander Dilthey,[10] Mark Lathrop,[11,8] Tom H. Karlsen,[5,12] Andre Franke,[7] Miriam Moffatt,[13] William Cookson,[13] John Trowsdale,[3,4] Gil McVean,[10] Stephen Sawcer,[14] and Stephen Leslie[1,2,*]

Large population studies of immune system genes are essential for characterizing their role in diseases, including autoimmune conditions. Of key interest are a group of genes encoding the killer cell immunoglobulin-like receptors (KIRs), which have known and hypothesized roles in autoimmune diseases, resistance to viruses, reproductive conditions, and cancer. These genes are highly polymorphic, which makes typing expensive and time consuming. Consequently, despite their importance, KIRs have been little studied in large cohorts. Statistical imputation methods developed for other complex loci (e.g., human leukocyte antigen [HLA]) on the basis of SNP data provide an inexpensive high-throughput alternative to direct laboratory typing of these loci and have enabled important findings and insights for many diseases. We present KIR*IMP, a method for imputation of KIR copy number. We show that KIR*IMP is highly accurate and thus allows the study of KIRs in large cohorts and enables detailed investigation of the role of KIRs in human disease.

Introduction

Over the past decade, studies of the genetics of human disease have benefitted greatly from the interrogation of large cohorts of samples genotyped at hundreds of thousands of markers. Formal genetics has extensively been replaced by the analysis of large amounts of SNP genotype or, more recently, sequence data, as the cost of obtaining such data has dramatically reduced, in part because of automation. In spite of these advances, some regions of the genome are refractory to detailed investigation because performing automated typing is difficult. This is because they are highly variable between individuals or because they exhibit copy-number variation (CNV). One such region is the major histocompatibility complex (MHC). Allelic typing of human leukocyte antigen (HLA) class I and II genes within the MHC is critical for transplantation and is informative for many disease associations. Until recently, the high cost of accurate HLA typing has precluded large-scale, disease-association studies for HLA alleles. The application of statistical methods to typing alleles via linkage disequilibrium with combinations of adjacent SNPs, known as imputation, has allowed large numbers of samples to be typed with high accuracy[1–5] so that massive cohorts of affected and control individuals can be studied.[6–8]

Another genomic region of interest in this regard encompasses the KIR genes, which are part of the leukocyte receptor complex (LRC) in human chromosomal region 19q13.4. KIR genes encode receptors that are expressed on natural killer (NK) cells and some T cells. KIRs are highly variable in terms of gene arrangement and copy number (see Appendix A for more information about KIR genes and nomenclature). Haplotypes can comprise 4–20 KIRs.[9] Some haplotypes are relatively common. The A haplotypes, for instance, are found in all populations studied. They are relatively stable in copy number, although the genes they contain can exhibit considerable sequence variation. In contrast, B haplotypes exhibit extensive CNV and vary in frequency. In individuals of European ancestry, in terms of copy number, 11 haplotypes are present at a frequency of over 1%.[9] Haplotypes are composed of combinations of motifs on either side of a recombination hotspot. Motifs are referred to as "centromeric" or "telomeric" with respect to this hotspot.

The high level of variation in KIRs, coupled with their functional relationship with HLA class I, suggests that their variation is driven by resistance to disease.[10,11] Indeed, combinations of HLA and KIRs have been associated with HIV infection, autoimmune conditions, and cancers.[12] Further, they have significant relevance in clinical outcome in hematopoietic stem cell transplantation.[13] Weight at birth is subject to strong evolutionary selection, and combinations of *HLA-C* (MIM: 142840) and KIRs have been linked to birth weight and pregnancy conditions

[1]Statistical Genetics, Murdoch Childrens Research Institute, Parkville, VIC 3052, Australia; [2]School of Mathematics and Statistics, University of Melbourne, Parkville, VIC 3010, Australia; [3]Cambridge Institute for Medical Research, University of Cambridge, Cambridge CB2 0XY, UK; [4]Division of Immunology, Department of Pathology, University of Cambridge, Cambridge CB2 1QP, UK; [5]Research Institute of Internal Medicine, Department of Cancer Medicine, Surgery, and Transplantation, Oslo University Hospital Rikshospitalet, Postboks 4950, Nydalen, 0424 Oslo, Norway; [6]Norwegian PSC Research Center, Division of Cancer, Surgery, and Transplantation, Oslo University Hospital, Postboks 4950, Nydalen, 0424 Oslo, Norway; [7]Institute of Clinical Molecular Biology, Christian-Albrechts University of Kiel, Schittenhelmstraße 12, 24105 Kiel, Germany; [8]Fondation Jean Dausset, Centre d'Etude du Polymorphisme Humain, 27 Rue Juliette Dodu, 75010 Paris, France; [9]RIKEN Center for Integrative Medical Sciences, 1-7-22 Suehiro-cho, Tsurumi-ku, Yokohama, Kanagawa 230-0045, Japan; [10]Wellcome Trust Centre for Human Genetics, University of Oxford, Roosevelt Drive, Oxford OX3 7BN, UK; [11]McGill University and Génome Québec Innovation Centre, Montreal, 740 Dr. Penfield Avenue, Room 7104, Montreal, QC H3A 0G1, Canada; [12]K.G. Jebsen Inflammation Research Centre, Institute of Clinical Medicine, University of Oslo, Postboks 1171, Blindern, 0318 Oslo, Norway; [13]National Heart and Lung Institute, Imperial College London, Royal Brompton Campus, Dovehouse Street, London SW3 6LY, UK; [14]Department of Clinical Neurosciences, University of Cambridge, Cambridge CB2 0QQ, UK
*Correspondence: stephen.leslie@mcri.edu.au
http://dx.doi.org/10.1016/j.ajhg.2015.09.005. ©2015 The Authors
This is an open access article under the CC BY license (http://creativecommons.org/licenses/by/4.0/).

such as pre-eclampsia (MIM: 189800).[12] More generally, co-evolution with HLA, with varying types of selection, has strongly influenced patterns of diversity in the KIR region.[14]

In view of their importance for human disease, a method is necessary for rapidly and inexpensively typing KIRs for large cohorts of individuals. So far, KIR typing has involved laborious, time-consuming methods, except in one case where variation in *KIR3DL1* and *KIR3DS1* (MIM: 604946) copy number was tagged with a single SNP.[15] SNP-based imputation has proved invaluable, and now essential, for large-scale HLA typing. Given this, and the availability of extensive SNP data, we here explore its potential for KIR. The complexities of the KIR region, namely the high level of CNV and the high homology shared by independent alleles and loci, mean that methods that have been developed for HLA are not necessarily applicable to KIRs. Thus, we have developed KIR*IMP, a technique designed specifically to impute KIR CNV and haplotypes defined by KIR copy number. We also assess the performance of some existing HLA-imputation methods applied to KIRs and show that KIR*IMP outperforms these methods.

Material and Methods

We first give an overview of our approach before describing each of the steps in more detail. Readers may skip directly to the Results section after reading the overview.

Overview

In typical genetics applications, imputation methods use a set of individuals for whom SNP genotype data are available and the variation to be imputed (the "target variable," e.g., HLA or KIR alleles) is also known. This is known as the "training data" or "reference panel." A statistical model is fitted to the training data. This associates the SNP variation to the target variable. Using this model, one can then impute the target variable for individuals for whom only the relevant SNP genotypes are available. Typically, this results in a probability distribution over the possible types, and individuals are usually assigned the type with the highest probability from this distribution. Our method, KIR*IMP, can impute a number of target variables representing different definitions of KIR loci: KIR gene copy number, KIR A or B haplotype, and gene-content haplotypes (see KIR and SNP Typing below for details). These latter two are defined by the combinations of individual KIR genes that are present on a haplotype.

We developed and validated KIR*IMP by using data from two cohorts of European individuals typed for both SNP genotypes and KIR copy number. A cohort of UK families formed our primary dataset; resolving phase and familial relationships resulted in a reference panel of 479 unrelated haplotypes ("UK reference panel"). We used this for training a statistical model on the basis of the random-forests algorithm. A separate panel of 1,338 unrelated and unphased Norwegian and German individuals was prepared for validation ("NG validation panel").

Both cohorts were typed for copy number at 17 KIR loci. For the UK reference panel, this also served as a basis for a fine-scale classification of KIR haplotypes (*KIRhaplotype*) and a standard broad classification of A and B KIR haplotypes (*AvsB*), giving us a total of 19 KIR types for this panel. SNP genotyping for both cohorts used the Illumina Immunochip array (see Figure 1) but resulted in different sets of SNPs after quality-control procedures. The results shown below use SNPs in a 400 kb region that covers the KIR genes.

DNA Samples

Our primary dataset, the UK cohort, comes from the UK DNA Banking Network (DBN), as described in Jiang et al.[9] It consists of families of individuals ascertained for having either asthma or atopic dermatitis. All subjects are of self-reported European ancestry. Of the 1,768 individuals reported in Jiang et al., 149 were removed because of missing data and haplotype ambiguity, leaving 1,619 individuals in 419 families. All of these individuals were typed for KIR copy number at all loci. SNP genotypes were obtained for 998 individuals from 449 families, which partially overlapped the above set. For logistical reasons, we were not able to obtain both KIR and SNP types for exactly the same individuals. The overlap between the two sets was 698 individuals from 343 families; these individuals were considered for inclusion in the UK reference panel, but all individuals were used for phasing and filtering (see below).

The Norwegian-German cohort consists of 1,338 unrelated Norwegian and German affected individuals and healthy control individuals from a study of primary sclerosing cholangitis.[17] All of them were typed for KIR copy number at all loci and for SNP genotypes.

The collection of data complied with relevant ethical standards and procedures and was approved by the ethics committees overseeing research at each of the respective institutions.

KIR and SNP Typing

Copy-number typing of 17 KIR loci was done by qPCR as described in Jiang et al.[9] These include KIR genes, pseudogenes, and major alleles of some genes, which we collectively refer to as "loci" for convenience; see Appendix A for more details about the nomenclature for the loci and Table S1 for their relationship with the underlying genes, including MIM references. The KIR genes are located in the band 19q13.42. For clarity, in the text below we define the "extended KIR region" as part of chromosome 19, including 19q13.42 and everything telomeric from it. According to coordinates from GRCh37 (Genome Reference Consortium), this corresponds to all positions greater than 53.6 Mb.

For the UK cohort, the availability of KIR types for relatives allowed resolution of phase and identification of whole haplotypes. We used the definitions of the haplotypes from Jiang et al.,[9] including their nomenclature of simple numbering from 1 to 71. In addition, we also used the broad A/B haplotype-group classification. We refer to these as the *KIRhaplotype* and *AvsB* types. Table S2 shows how these relate to the copy number at each of the typed loci. This gave us a total of 19 KIR types for this cohort: 17 loci plus two extended haplotype classifications.

For the Norwegian-German cohort, we only had available the copy-number types on a per-individual basis. Because the individuals are unrelated, we did not resolve these into per-haplotype copy-number types as we did for the UK cohort.

All KIR typing was conducted at the Cambridge Institute for Medical Research, University of Cambridge, UK.

Typing of SNP genotypes was done with the Illumina Immunochip array.[18] The UK cohort was typed at the Centre National de

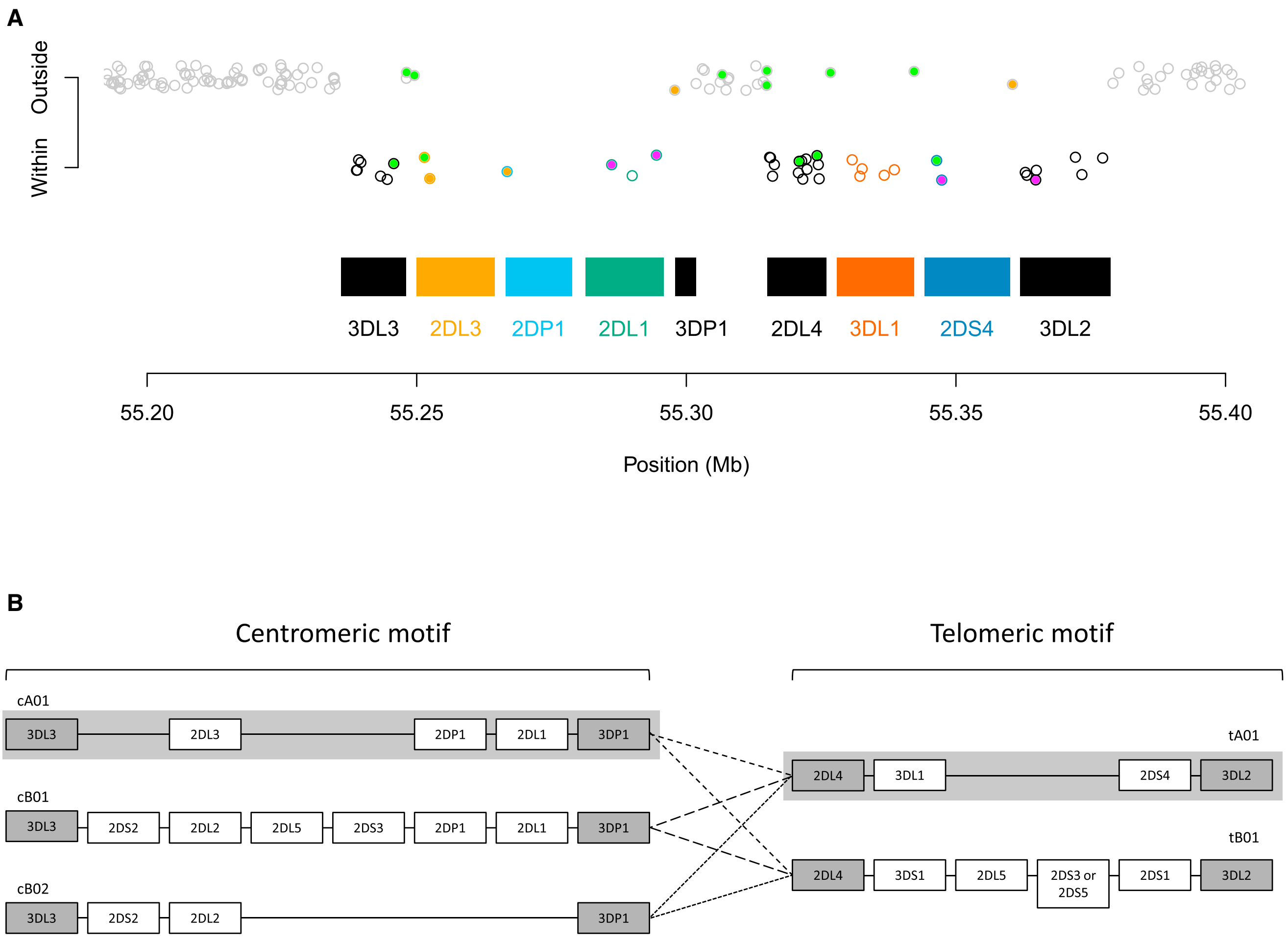

Figure 1. The KIR Region

(A) Immunochip SNPs and KIR genes. The relative genomic position of Immunochip SNPs and KIR genes on chromosome 19 according to the human reference genome (GRCh37) and the annotation provided with the Illumina Immunochip array. KIR genes are shown as rectangles. Only some KIR genes are present, consistent with the fact that the reference genome is an A haplotype. SNPs are shown as circles. They vary in y coordinates and border color on the basis of whether they are annotated as being within a particular KIR gene (including introns). SNPs that were selected as being most informative are shaded in light green if they had good clustering and magenta if they had poor clustering. SNPs that were monomorphic in the UK reference panel are shaded in orange.

(B) Composition of common KIR haplotypes. The common KIR haplotypes are composed of different centromeric (cA01, cB01, and cB02) and telomeric (tA01 and tB01) motifs, each of which differs in the content and arrangement of genes. Framework genes, which are found at the ends and near the middle of the region on nearly all haplotypes, are shaded gray. Some genes (e.g., *KIR2DL5*) can be located in both motifs. Different centromeric motifs can be paired with different telomeric motifs through the central reciprocal recombination hotspot between *KIR3DP1* and *KIR2DL4*, as indicated by different dashed lines. KIR haplotypes can be classified into two categories: group A haplotypes (shaded gray) and group B haplotypes (unshaded). Group A is composed of only cA01 and tA01 motifs, which have fixed gene-content with one activating gene (*KIR2DS4*), which on many A haplotypes contains a deletion rendering it non-functional (see Appendix B). Group B comprises at least one motif of type cB01, cB02, or tB01, which has variable gene-content between framework genes and more than one activating KIR gene. Less common KIR haplotypes might differ (with slightly different arrangements and copy number of the KIR genes[9]) from the ones shown here. This panel is adapted from Roberts et al.[16] and relates to haplotypes of European ancestry.

Génotypage, France. The Norwegian-German cohort was typed at the Institute of Clinical Molecular Biology, Christian-Albrechts University of Kiel, Germany.

SNP Genotype Calling and Quality Control

SNP genotypes for both cohorts were called separately via the optiCall method.[19] For the UK cohort, this resulted in 4,121 successfully called SNPs, of which 1,118 were in the extended KIR region, on chromosome 19. Of these, 1,078 were polymorphic in our sample and were used for analyses. For the Norwegian-German cohort, this resulted in 2,904 successfully called SNPs, of which 888 were in the extended KIR region, on chromosome 19. Of these, 881 were polymorphic in our sample and also present in the UK cohort. SNP and sample quality control before the analysis, including exclusions based on non-European ancestry, was performed as described in Liu et al.[17] A handful of SNPs were subsequently excluded after manual inspection of the genotype calls and SNP-intensity measurements (see SNP Sets and SNP Selection below). We found that SNP genotype call rates can vary substantially depending on the calling method used. We recommend that calling and quality-control procedures be carefully scrutinized

for maximizing the number of well-called SNPs, especially from the set of most informative SNPs from our analysis.

Phasing

We phased the SNPs for the UK cohort by using SHAPEIT[20,21] and duoHMM.[22] We did not use a reference panel for phasing (there was no readily available panel based on the Immunochip); however, we did use the "HapMap phase II b37" recombination map provided for use with SHAPEIT (see Web Resources). We used the parameter settings recommended by the authors of duoHMM. The resulting phasing is expected to be of very high accuracy because of the family structure of the data. We also phased the Norwegian-German SNP data with SHAPEIT. We used the phased UK cohort as a reference panel for phasing (because this is expected to be of very high accuracy), the same recombination map, and the default SHAPEIT parameter settings.

Producing the UK Reference Panel

The UK dataset consisted of a combination of individuals, some of whom had missing KIR types and some of whom had missing SNP data. Furthermore, because of the family structure, this dataset contained haplotypes that were identical by descent. To create an imputation model, we needed a reference panel with both SNP and KIR data that consisted of unrelated haplotypes. We produced this as follows.

First, we determined the parent of origin for each haplotype where it was unambiguous. We did this separately by using the KIR types and the SNP genotypes (on the respective subsets of the dataset where these data were available). For KIR types, we compared the types directly and required an exact match. For SNP genotypes, we used all SNPs across 19q13.42 and required fewer than 20 mismatches to declare that the haplotypes matched.

Second, we merged the parent-of-origin information inferred from the KIR and SNP comparisons and excluded any haplotypes for which this could not be done unambiguously.

Third, we excluded related haplotypes from each family. For example, when data for both parents were present, we only kept the parental haplotypes. When data were available for only one parent, then we kept the parent and also any other haplotype that was identified among the offspring and was not present in the parent.

The final UK reference panel consisted of 479 haplotypes. For each one, we had 1,078 SNPs in the extended KIR region and the full set of KIR types. Summaries of the allele-frequency distributions are shown in Table S3 and Figure S1.

Producing the NG Validation Panel

Of the individuals in the Norwegian-German cohort, we took only those for whom we had both KIR and SNP types. This sample consisted of unrelated individuals, so no further filtering of individuals was required.

For this dataset, we phased the SNPs; however, the KIR types were not phased (unlike the scenario for the UK cohort, for which we had family relationships that allowed us to phase the KIR types as well). Therefore, we used this as a validation sample. We checked that the KIR copy-number distribution was consistent with that of the UK reference panel under the assumption of Hardy-Weinberg equilibrium (the comparison is not shown, although this can be reproduced from the data in Table S3).

To make it compatible with the UK reference panel, we excluded SNPs that were not present in both sets. We then aligned the SNP allele coding to that of the UK reference panel through comparison of nucleotide type (purine or pyrimidine) and allele frequencies.

The final NG validation panel consisted of 1,338 individuals (2,676 haplotypes). For each one, we had 881 phased SNPs in the extended KIR region and unphased copy number for all typed KIR loci. Summaries of the allele-frequency distributions are shown in Table S3 and Figure S1.

Predicting KIR Types

We used a random-forest model[23] as the basis for our imputation method. This operates by fitting a large number of "classification trees" to the training data. A single classification tree is a hierarchical structure consisting of "internal nodes" corresponding to specific SNPs, "leaf nodes" corresponding to different values of the target variable (KIR types), and "branches" connecting the nodes that correspond to alleles at the respective SNPs. A path through a tree defines a haplotype by traversing a sequence of branches that correspond to specific alleles at a set of SNP loci (typically a small subset of those in the training data). The set of all possible paths thus corresponds to a set of haplotypes, each of which has an associated prediction for the target variable (via the leaf nodes). The trees are constructed iteratively with standard algorithms for this purpose,[23] such that new SNP loci are selectively added to the tree, leading the resulting haplotypes to be informative for the target variable.

A random forest consists of a large number of such trees, referred to as an "ensemble." Because the tree-fitting algorithms are deterministic, two techniques are used to create variation among the fitted trees. First, a random subsample (with replacement) is taken from the reference panel and is used as training data for each individual tree (in other words, each tree is based on a slightly different training dataset). This is known as "bootstrap aggregation" or "bagging."[24] Second, during the tree-fitting algorithm, only a random subset of the SNPs are considered candidates for inclusion as an internal node. This is known as the "random subspace method"[25] or "attribute bagging."[26] These techniques lead to reduced correlation between the fitted trees and improved accuracy of the overall ensemble. We used the implementation of random forests in the randomForests R package.[27]

Although random forests are not based on a population genetic model, some insight can be gained into how they model the data and how this relates to the underlying genetics. A classification tree is somewhat analogous to a multi-marker SNP tag, but there is explicit sharing of SNPs across tags, and the tags form a proper partition of the target variable (see Figure S2). A random forest then adds an extra layer of flexibility by taking a large number of such trees and averaging them, effectively creating a mixture of such haplotype distributions. Thus, we can view random forests as a sophisticated multi-marker approach, in which the bagging and subsetting operations add substantial robustness.

We used the UK reference panel as the training set for all analyses and the KIR types as the target variable and fitted a separate model for each KIR locus. We used either all SNPs in the reference panel or a subset, depending on the analysis of interest.

Using a fitted model for imputation involves applying each tree to a new dataset of SNP alleles and taking the set of predictions across the ensemble, which can be summarized as a probability distribution. To obtain a single predicted type, the simplest

approach is to take the one with the highest probability. We did this for most analyses we present. In addition, we also explored the use of probability thresholds, as is often done in practice for association analyses. This involves setting a lower limit for the probability required for making an imputation "call." Imputations that do not meet the threshold are not called; they are treated as missing data. The concept is that by omitting the less certain imputations, we obtain a set of calls with higher accuracy. The aim is to set a threshold that gives acceptable call rates (the proportion of haplotypes or individuals with an imputation call) and high accuracy.

Parameter Tuning

In a random-forest model, the key parameter that typically needs tuning is the number of SNPs, m, to be subselected at each attribute bagging step. The default value is the square root of the total number of SNPs,[27] which gives $m = 17$ with the use of ~300 SNPs (a typical number for many of our analyses). We explored a range of different values for m and evaluated performance by using the *UKsnps* set (see SNP Sets and SNP Selection below) and out-of-bag (OOB) accuracy (see Assessing Accuracy below) on the UK reference panel (see Figure S3). We found that a higher value of m increased accuracy for some KIR loci. This is presumably due to the fact that only a small number of SNPs are informative for such loci (see Results), and if m is too low, then many trees will not include any of these SNPs. We decided to set m at 100 in order to have sufficient SNPs to increase accuracy at all loci without losing accuracy as a result of decreased diversity between trees in the ensemble. Unless otherwise stated, we used $m = 100$ throughout.

We also explored varying the number of trees used and the maximum number of nodes for each tree, but we found that these did not noticeably affect the accuracy (data not shown). We used 1,000 trees for each model fitted and no limit on the number of nodes.

Assessing Accuracy

We assessed imputation accuracy in two ways:

- UK cross-validation analysis: 5-fold cross-validation using the UK reference panel for all 19 KIR types. This was done on a per-haplotype basis, whereby the imputation accuracy was calculated as the proportion of haplotypes with correctly imputed KIR types.
- NG validation analysis: Independent validation using the NG validation panel for KIR copy-number types only. This was done on a per-individual basis, whereby the imputation accuracy was calculated as the proportion of individuals with correctly imputed KIR types.

For some analyses, instead of the above, we used the OOB accuracy to assess imputation performance. We calculated this during the model-fitting process by using the haplotypes excluded from the fit for each tree (because of bagging) effectively as a validation set (for that tree only) and aggregating the predictions on these haplotypes across the whole ensemble. This mimics cross-validation but is computationally more convenient. We found that it was an adequate proxy for full cross-validation (Figure S4) and thus chose to use it for some analyses.

To assess imputation accuracy for particular alleles, we calculated the "sensitivity" and the "positive predictive value" (PPV). These are, respectively, the proportion of correctly imputed types among all haplotypes with that allele and the proportion of correctly imputed types among all haplotypes imputed with that allele.

To assess the relative contribution of each SNP to the prediction accuracy of the model, we used the permutation strategy described by Breiman.[23] For each tree, the OOB accuracy is calculated for two versions of the data: (1) the actual data and (2) the data after the alleles for a particular SNP are permuted across all samples. The resulting difference in prediction accuracy is averaged over all trees and normalized by the SD of these differences to give a "variable-importance" score for the SNP. This is repeated for all SNPs.

Credible Intervals

To assess the uncertainty of the imputation-accuracy estimates and the calibration plot, we calculated 95% credible intervals on the basis of a binomial model and a uniform prior distribution. The binomial model assumes that the imputed values across individuals are independent. For cross-validation, this assumption does not hold because the training sets partially overlap (between cross-validation folds), and in fact no known estimator of the variance works well in general.[28] Thus, we treat this as an approximation only. For the independent validation, the independence assumption is reasonable.

SNP Sets and SNP Selection

We explored different choices of SNP sets by using the UK reference panel. Initial exploration with large numbers of SNPs (e.g., all 1,078 SNPs in the extended KIR region) showed that those with the highest variable-importance scores were concentrated closest to the KIR genes. We therefore focused on 305 SNPs that had genomic coordinates (GRCh37) between 55.1 and 55.5 Mb and were polymorphic in the UK reference panel. Using this smaller set did not reduce accuracy (data not shown).

Of these 305 SNPs, four were eventually excluded because of poor clustering of genotype intensities (see below). We refer to the resulting 301 SNPs as the *UKsnps* set and the full 305 as the *UKplus4snps* set. The cross-validation analyses were done with the *UKsnps* set. The independent validation analysis required SNPs present in both panels. Of the *UKsnps* set, 231 SNPs were also present in the NG validation panel; we refer to this as the *UKNGsnps* set.

To explore how the inclusion or exclusion of key SNPs affects accuracy, we fitted a series of models where we iteratively removed the SNP with the highest variable-importance score; at each step, we fitted a model to the remaining SNPs and also one to the set of removed SNPs (in the latter case, we set the tuning parameter m to the total number of SNPs). This allowed us to determine how the accuracy increased or decreased as we gradually added or removed, respectively, the most influential SNPs (Figure S5). Although the general relationship was as expected, we observed some non-monotonicity. This is due partly to the stochastic nature of the random-forest model (which is especially visible when the y axis range is narrow) and partly to its sensitivity to which SNPs are included (particularly for the models fitted with very few SNPs).

To create a single set of the most informative SNPs, for each KIR locus we took the minimal number of SNPs necessary for reaching high accuracy on the basis of a visual assessment of the results of the inclusion and exclusion experiments and took their union across all loci (the number of SNPs taken from each locus is shown in Figure S5).

We inspected plots of SNP-intensity measurements and genotype calls for these SNPs to check whether their genotype calls

were reliable (Figure S6). We looked for evidence that the intensity clusters inferred by the genotype-calling algorithm either did not reflect true underlying SNP genotypes (e.g., that the inferred clusters did not form visually distinct groups) or were not being reliably called in a way that would be reproducible across samples (e.g., that the underlying clusters did not show enough separation). We refer to SNPs that we judged as failing this visual test as having "poor clustering" and refer to those that passed as having "good clustering." We excluded the SNPs with poor clustering from all validation and comparison analyses. It is likely that other SNPs are also candidates for exclusion for similar reasons. It is impractical to inspect plots for all SNPs, and we did not do this. Instead, we concentrated on the most informative SNPs because we reasoned that the others would minimally influence the final outcome.

We refer to the set of most informative SNPs, excluding those with poor clustering, as the *UKselectedSnps* set. This set contains 12 SNPs (see Results). Models fitted with this set used $m = 10$ for the tuning parameter. We refer to the SNPs in the *UKsnps* set, which were not in the *UKselectedSnps* set, as the *UKnotSelectedSnps* set.

Other Imputation Methods

We compared KIR*IMP against three existing methods developed for imputing HLA alleles: HLA*IMP:01,[1,2] HLA*IMP:02,[3] and HIBAG.[5] In the comparison, we also included the simple approach of using a single tag SNP. We did not include SNP2HLA,[4] another prominent HLA-imputation method, because its implementation is specific to HLA and is not easily amenable to working with other genes (although in principle, this is possible).

We formatted our data in such a way that the methods would treat each KIR locus as if it were a HLA gene and treat each type at each locus as a HLA gene allele.

For the methods that operate on a per-haplotype basis (HLA*IMP:01 and tag SNPs), we evaluated them by using both validation strategies described earlier. The standard implementations for the remaining methods (HLA*IMP:02 and HIBAG) operate on a per-individual basis by using unphased SNP genotypes as input. For these methods, we created a synthetic training set from the UK reference panel by randomly pairing the haplotypes (without replacement) to form pseudo-individuals (one haplotype had to be discarded because there was an odd number). The output from these methods was also unphased; for simplicity, we only show the performance of these methods for the NG validation analysis, which is on a per-individual basis. We used unthresholded calls for all comparisons.

All methods were provided with the physical positions of the SNPs according to GRCh37. HLA*IMP:01 required a recombination map, and we provided it with the same one (from the 1000 Genomes Project) used for phasing the data (see above). HLA*IMP:02 required a (single) physical position for each of the KIR loci. We created surrogate positions for each locus in a number of ways. For KIR genes and pseudogenes, if Immunochip SNPs were annotated as being within them, we took the mean of their positions. If there were no such SNPs, we took the mean of the gene's annotated endpoints. For genes that did not appear on the A haplotype, and thus did not have an annotated position, we found the closest SNP in each of the two most adjacent genes and took the mean of their annotated positions. For non-copy-number KIR loci (such as *KIRhaplotype*), we took the weighted (by the variable-importance score) mean of the positions of the top five most informative SNPs from our KIR*IMP model for that locus. We ran each method by using its default parameter settings. For HLA*IMP:02, the localization feature was turned on for all loci.

We selected tag SNPs for each copy-number locus and *AvsB* by taking the SNP that gave the highest prediction accuracy (in the training set) when it was used as a simple classifier rather than taking the one with the highest Pearson correlation, as is often done. We used this criterion because it is the metric we used to compare the different methods. For reporting actual tag SNPs and their properties, we used statistics based on the full training data. For reporting comparisons against other methods, we used statistics from the validation strategies described above. These can give rise to slightly different accuracy estimates; we used the full training data for the former in order to obtain a single tag SNP, which is not guaranteed to be the best tag under each fold of cross-validation.

Other SNP Genotyping Arrays

To assess imputation performance on using SNP data from genotyping arrays other than the Immunochip, we fitted models by using a subset of the SNPs corresponding to those that could be typed on each specific array. Specifically, we obtained lists of SNPs for many Illumina and Affymetrix arrays (from their respective websites; see Web Resources). For each array, we took the intersection between the SNPs on the array and those in the *UKsnps* set (by matching on the GRCh37 genomic coordinates) and used the UK reference panel to fit a model with just those SNPs. This mimics the (best-case) scenario, where all the SNPs in the intersection are perfectly typed in a study sample. In practice, typing for some SNPs will most likely fail, leading to possibly lower imputation accuracy.

Results

Imputation Accuracy

We assessed accuracy by 5-fold cross-validation on the UK reference panel and independent validation on the NG validation panel. Table 1 shows accuracy estimates from these analyses. For typing copy number in the UK reference panel and using the *UKsnps* set, we observed greater than 98% accuracy for the majority of loci, at least 95% accuracy for half the remaining loci, and better than 90% for the rest. For distinguishing the broad A and B haplotype groups, KIR*IMP achieved 98.5% accuracy. For the more challenging task of imputing the fine-grained haplotype groups, accuracy was 87.1%. As discussed below, this is mainly due to the presence of many rare haplotypes, which are naturally hard to impute. We also show below that we could improve accuracy by limiting ourselves to the types imputed with higher certainty. These results are based on all available SNPs in the region and our training set. Table 1 also shows that using the smaller *UKNGsnps* set, emulating applying KIR*IMP in practice, had little impact on performance.

The NG validation results in Table 1 confirm that KIR*IMP is accurate at typing copy number for the majority of KIR loci. These results are per individual, so they are not directly comparable to the UK results, which are per haplotype. To determine whether the reported accuracies are similar for each analysis, we calculated the expected

Table 1. Imputation Accuracy

	UK			NG
**Locus**	***UKsnps***	***UKNGsnps***	***UKselectedSnps***	***UKNGsnps***
KIRhaplotype	87.1	85.4	85.6	–
AvsB	98.5	98.5	98.5	–
KIR2DS2	99.0	99.0	99.0	99.8
KIR2DL2	98.3	98.3	98.3	98.0
KIR2DL3	98.8	98.8	98.8	98.6
KIR2DP1	91.0	90.0	92.5	85.1
KIR2DL1	90.6	89.3	91.9	82.2
KIR3DP1	96.9	96.7	96.5	91.6
KIR2DL4	97.1	97.1	96.9	91.1
KIR3DL1ex4	99.0	99.0	99.0	94.5
KIR3DL1ex9	98.8	98.8	98.8	94.7
KIR3DS1	97.5	97.5	97.7	96.2
KIR2DL5	92.5	90.2	92.1	82.2
KIR2DS3	90.4	90.4	89.6	81.3
KIR2DS5	95.8	96.2	94.6	95.0
KIR2DS1	99.0	99.0	99.0	94.9
KIR2DS4TOTAL	98.8	98.8	98.8	94.8
KIR2DS4WT	99.6	99.6	99.6	98.6
KIR2DS4DEL	98.8	98.8	98.8	96.9

Estimates of KIR*IMP imputation accuracy from the validation analyses. For the UK cross-validation analyses, the percentage of correctly imputed haplotypes is shown, whereas for the NG validation analyses, the percentage of correctly imputed individuals is shown (thus, the two are not directly comparable). The three columns for the UK correspond to different SNP subsets used for training the model (*UKsnps*, *UKNGsnps*, and *UKselectedSnps*), as described in the main text. Note that *KIRhaplotype* and *AvsB* are only defined on a haplotype level and thus are not available in the NG validation panel.

per-individual accuracy by using the UK imputation results and pairing haplotypes at random. If this assumption is adequate, and the amounts of KIR genetic variation in the UK and NG panels are sufficiently representative of each other, these should roughly match. This comparison is shown in Figure S7; they indeed match well.

Previous imputation approaches (for HLA) have shown that accurate imputation of a given variant often depends on how many instances of the variant are represented in the training data.[1] This is also true for KIR imputation. Figure 2 shows the relationship between imputation accuracy and the number of times a gene or haplotype appears in the UK reference panel. Although in some instances KIR*IMP obtained high accuracy with a relatively small number of training examples, high accuracy was only reliably obtained with over 100 such examples.

KIR*IMP assigns probabilities to predictions. An important question is whether these are meaningful, i.e., does a probability of 0.9 mean that about 90% of such imputations are correct? Figure 3 shows an assessment of this by comparing the level of certainty against the observed accuracy for imputations with that level of certainty. The probabilities for our model are mostly well calibrated, in the sense described above. If anything, they are slightly conservative, such that the imputations of moderate certainty (with probability of around 0.8) are slightly more accurate than expected.

We assessed the effect of imposing a probability threshold on the imputations. Figure S8 shows a comparison of accuracy against call rate for various thresholds. For example, a threshold of 0.5 substantially increases the accuracy of the *KIRhaplotype* imputations from 87% to 92% while retaining a call rate of above 90%. In this case, the haplotypes that are refractory to a call are primarily the rarer ones, which are imputed with less certainty. Stricter thresholds result in higher accuracy: a threshold of 0.7 results in accuracy above 95% at all loci and call rates varying roughly from 80% to 100%.

Given that we fitted models both for whole haplotypes (*KIRhaplotype*) and on a per-gene basis, a natural question is whether these give equivalent performances. We assessed this by converting the whole-haplotype imputations, which by nature were fine-grained, into locus-specific ones by "coarsening" them to copy-number values for each locus. In Figure S9, a comparison of the two approaches shows that they are effectively equivalent.

Comparison of Imputation Methods

There are no current methods specifically designed for imputing KIR variation and thus no clear candidates for comparison. It is possible to adapt existing imputation methods for other gene families to type KIR variation. For the purposes of comparison, we did this with several leading HLA-imputation algorithms (HLA*IMP:01, HLA*IMP:02, and HIBAG). We also included the simple method of using a single tag SNP. Figure 4 shows a comparison of all of these methods. To the extent possible, we assessed each method by using the same validation approach as for KIR*IMP. Standard implementations of HLA*IMP:02 and HIBAG use only unphased data, and thus we only show their performance for the NG validation analysis.

Similar conclusions can be drawn from both analyses in Figure 4. Most methods performed well for most loci. It was harder to impute a few loci, notably *KIR2DP1*, *KIR2DL1*, *KIR2DL5*, and *KIR2DS3*, highlighting differences between the methods. For these loci, KIR*IMP was the most accurate. The slightly poorer performance of the HLA-based approaches here might be due to their making assumptions that are likely to be inappropriate for KIR (see Discussion).

For many KIR loci, single tag SNPs do quite well at capturing variation in copy number (see Table S4) in that they perform similarly to using all SNPs and a more sophisticated model. This highlights the fact that, for these loci, a small number of SNPs is sufficient for building accurate imputation models. We explore this further in the next section.

Overall, KIR*IMP was consistently the (uniquely or equally) most accurate method for imputing KIR types.

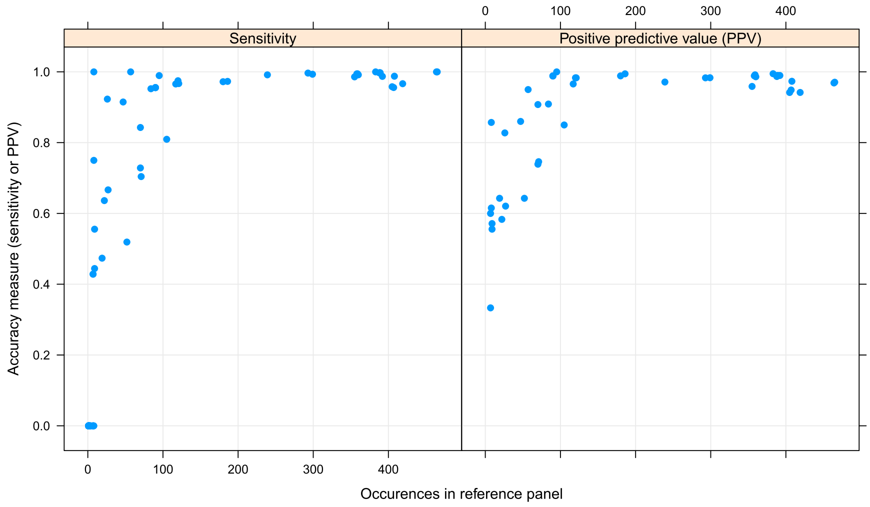

Figure 2. Per-Allele Imputation Accuracy
Estimates of the sensitivity and positive predictive value (PPV) for each KIR-type allele (i.e., the different possible values for each of our KIR loci) from the UK cross-validation analysis of KIR*IMP. These are plotted against the number of times each allele appeared in the UK reference panel. Each point corresponds to a single allele.

In addition, KIR*IMP has two important advantages: (1) it has fewer requirements (e.g., no need for a recombination map), making it more convenient to train and use, and (2) it is very fast (fitting the model typically takes much less than 1 min per locus with the use of our reference panel and a modern computer, and imputing with a fitted model is almost instantaneous). Further, it provides imputations on a per-haplotype basis, which is of greater interest than unphased types in applications. It does require that the input SNP data be phased, but this is accurately and efficiently achieved via existing methods (e.g., SHAPEIT[20,21]).

Choice of SNPs

We had available SNPs from a genotyping array (Immunochip) specifically designed to have high coverage of the KIR region. This enabled us to develop an imputation method with high accuracy. An important question is whether it is possible to do this with a small set of SNPs. This would lend extra flexibility to the method, allowing it to be used in situations where typing samples on such an array is not feasible but where typing them at a specific small number of SNPs is.

We first consider the related question of which SNPs are contributing the most to imputation performance. Figure S10 shows the variable-importance scores for all SNPs at each locus. For most loci, a few SNPs show high importance. We also see a spatial pattern that accords with our knowledge of the relative position of KIR genes: the SNPs showing up as most important for each gene tend to be located on either the centromeric or the telomeric side of the region, consistent with the typical location of the gene. Four loci have SNPs with high importance on both ends of the region: *KIR2DP1*, *KIR2DL1*, *KIR2DL5*, and *KIR2DS3*. These are precisely those where KIR*IMP had higher imputation accuracy than the HLA-based methods, indicating that those methods might not be adequately capturing information from all parts of the region. *KIR3DP1* and *KIR2DL4* did not have any particular SNPs with very high importance. These loci showed very little overall variation (nearly all haplotypes have copy number 1), and none of the models could impute their rare variants accurately (in fact, nearly all imputed values, for all models except HLA*IMP:01, were for copy number 1). The absence of SNPs with high importance merely reflects this fact.

The fact that only a small number of SNPs showed high importance for most loci suggests the possibility of training an accurate model with a few SNPs. To explore this, we ran a series of analyses to select and evaluate the most influential SNPs (see Material and Methods). Figure S5 shows the results from these experiments, and the final set of selected SNPs is shown in Table 2 and Figure 1.

A number of insights are evident from these results. First, the two haplotype-based types, *KIRhaplotype* and *AvsB*, require approximately ten SNPs to achieve accuracy almost similar to that of the full model. Removing those SNPs substantially decreased the accuracy for these types, indicating that most information is concentrated in a small group of SNPs. Second, we see differing behavior for the centromeric and telomeric KIR loci. Centromeric loci (e.g., *KIR2DS2*) required only one to two SNPs to achieve high accuracy, and performance degraded rapidly as these were removed. Many telomeric loci also had high accuracy with only one to two SNPs, but their performance did not degrade substantially when they were removed. Thus, there was more redundancy in the SNP information for the telomeric loci than for the centromeric loci.

We fitted models by using just the selected SNPs (in fact, just 12 of these, referred to as the *UKselectedSnps* set; see Material and Methods) to assess their utility for imputation. Figure 5 compares the performance of these models against those fitted with all SNPs and all but the selected SNPs. Two observations stand out from this comparison. First, the *UKselectedSnps* set performed similarly to using all SNPs. Second, the models based on the non-selected SNPs performed well for telomeric loci but poorly for centromeric loci, confirming our observation that there is very little redundancy in the SNP information for centromeric loci, i.e., specific SNPs are crucial for accurate imputation of centromeric loci. At this stage, we cannot rule out the possibility that this might simply be due to the design

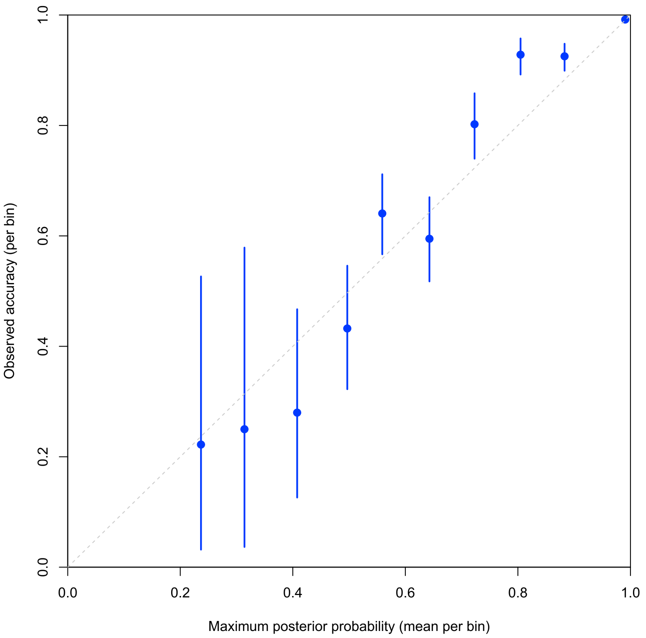

Figure 3. Calibration of Imputation Probabilities
A calibration plot of the KIR*IMP imputation probabilities. The probabilities used are those associated with the OOB imputations across all KIR loci, imputed on the UK reference panel by KIR*IMP trained with the *UKsnps* set. The imputed KIR types are grouped by their maximum posterior probability (MAP) into ten bins of equal width (on the probability scale) covering the range of the probabilities. For each bin, the observed imputation accuracy and corresponding 95% credible interval (see Material and Methods) are plotted against the mean MAP. Note that the number of values in each bin varies, as reflected by the differing widths of the intervals. Perfect calibration is indicated by the dashed line.

of the SNP array and that alternative choices of SNPs might provide more redundancy.

A related question is how accurately we can impute KIR by using SNP data from a different array. For each array, we assessed this by using those SNPs that are both present in *UKsnps* and typed on the array (assuming that all are perfectly typed). The results are shown in Table S5; we note that fairly high accuracy can potentially be achieved with many of the arrays.

Discussion

We have developed a statistical imputation method for typing KIR gene copy number from SNP genotypes and have shown that it is highly accurate. By leveraging existing SNP-genotyping technology, this method allows high-throughput, low-cost KIR typing, enabling a number of applications.

Uses for Our Method

As for HLA imputation, an important use of KIR*IMP will be for genetic association studies for diseases and complex traits. Of particular interest is the substantial number of autoimmune and other diseases for which HLA has been implicated. HLA class I molecules and KIRs are known to interact biologically,[12] suggesting that many such diseases are likely to also involve KIRs.

Many disease studies have typed their samples on the Illumina Immunochip or one of the other arrays we assessed. Our method can be immediately applied to these data for analyzing potential associations with KIRs. For other studies, if it is not feasible to type the samples on an appropriate array, an alternative is to type the small set of informative SNPs we have identified by using targeted methods, e.g., TaqMan.[29] We have shown that these SNPs are sufficient for accurate KIR imputation. Furthermore, for the centromeric genes, these SNPs are also necessary, meaning successful imputation of these loci relies crucially on the availability of these markers (although future studies might discover alternative SNPs [not on the Immunochip array] that are also informative for these loci). This should guide designs of future arrays if they are to be used for KIR imputation.

We have shown that many KIR loci are well tagged by SNPs. Nevertheless, we recommend that KIR*IMP, in addition to other standard analyses such as single-SNP tests, be used for assessing disease association within the KIR region. Many of the KIR loci are more accurately imputed by KIR*IMP, and thus this approach will lead to greater power. For example, for the broad A/B haplotype-group classification, we achieve imputation accuracy of 98%, compared to 87% for the best tag SNP (the actual impact of this on power will vary depending on many factors and can be estimated by simulations or analytical approximations[30,31]). Given that interactions between HLAs and KIRs are expected, it is particularly important that typing is as accurate as possible: the power to detect interactions drops off rapidly as measurements become less accurate.[31] Furthermore, because KIR*IMP imputes variants of direct biological interest, any findings are more likely to be causal.[32]
In the future, as accuracy is improved, rapid determination of KIRs by imputation could be used for additional clinical purposes, for example, for rapid screening for determination of compatibility for transplantation.

Relationship to Existing HLA Methods

We explored existing methods for imputing gene variation, in particular those designed for HLA genes, for potential use in the KIR context. Even though they are not optimized for KIR variation, we found that these methods generally performed well, although none performed consistently the best across all loci.

Some of the methods rely on assumptions that, although accurate in other parts of the genome, are not appropriate for KIRs. These include the assumptions that (1) the relative genomic positions of SNPs and genes are fixed and known and (2) an accurate recombination map that adequately describes the LD patterns in the region is available. It is known that there is extensive structural variation between individuals, such that genes such as *KIR2DL5* appear either in the centromeric or the telomeric (or both) part of the region, making the notion of fixed relative

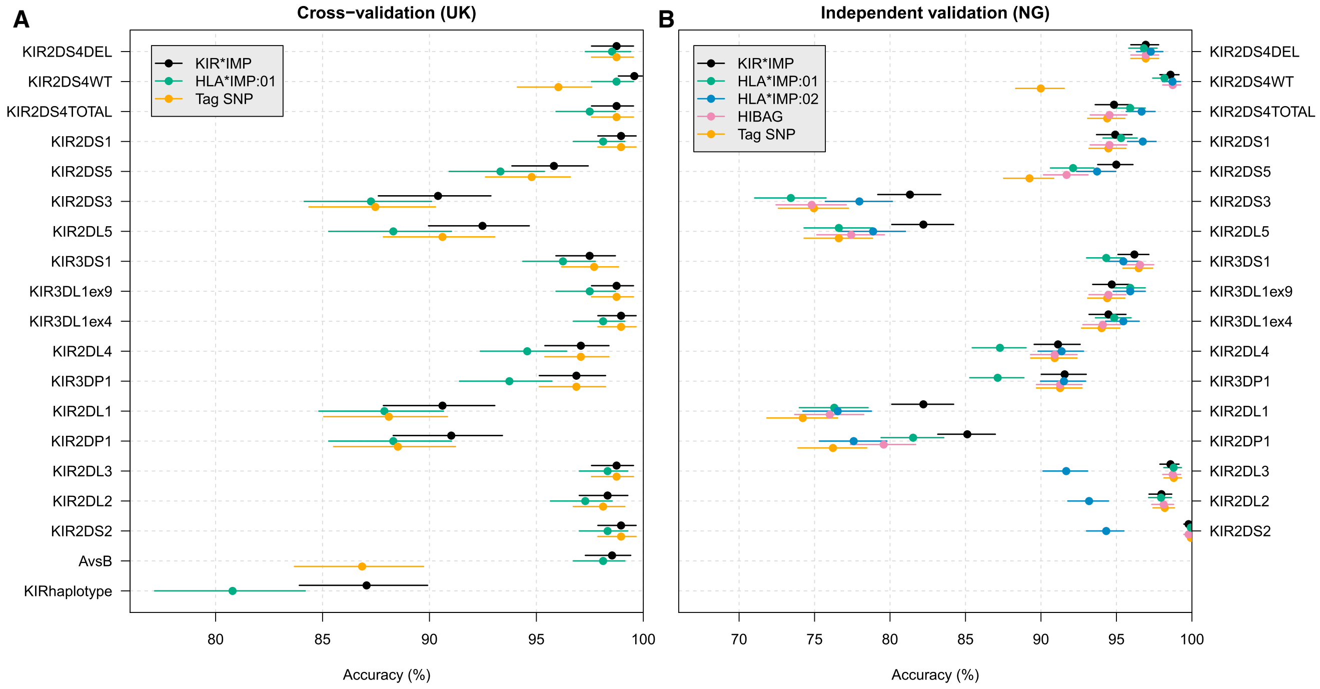

Figure 4. Comparison of Imputation Methods
Estimates of the imputation accuracy for the different methods and the associated 95% credible intervals (see Material and Methods).
(A) The percentage of correctly imputed haplotypes from the UK cross-validation analysis.
(B) The percentage of correctly imputed copy-number types for individuals from the NG validation analysis.

positions unrealistic. Furthermore, a recombination map is unlikely to properly capture the effect of extensive nonallelic homologous recombination that has shaped this region. This stimulated the development of KIR*IMP, which is based on the flexible random-forest model and does not rely on such assumptions. We expect that as we learn more about the structure of genetic variation in the KIR region, more-accurate models can be devised to outperform generic approaches such as random forests.

The model used by HIBAG is similar to a random forest in that it takes advantage of an ensemble of classifiers and uses techniques such as bagging. The main difference is that, for each classifier, HIBAG uses the haplotype distribution defined by all genotypes across a selected set of SNPs, whereas the classification trees used in a random forest allow different subsets of SNPs to define each haplotype (see Material and Methods and Figure S2). Another difference is in the implementation of phasing: HIBAG takes unphased genotypes as input and phases them by using an expectation-maximization algorithm, whereas KIR*IMP works directly on phased input (which can easily be obtained with the best current phasing algorithms).

Limitations of Our Method

Some limitations of KIR*IMP are worth noting. Our training data are exclusively of European ancestry. Therefore, KIR*IMP might not be accurate for non-European individuals, particularly those who do not share recent common ancestry with Europeans for their KIR genes. Although it is known that KIR genes can vary widely between people of different ancestry,[33] it is possible that the more common KIR haplotypes in our reference panel might appear with appreciable frequency in other populations as well. Another limitation is that we can only impute variants that exist in our reference panel and only do so accurately if there are sufficient examples. It will be difficult or impossible to impute rare types successfully.

The natural solution to both of these limitations is to increase the size and diversity of the reference panel. This has been successful for HLA imputation,[3] and we expect the same for KIR*IMP as more training data become available.

Possible Extensions

The SNPs on the Immunochip array all have annotated positions on the human reference genome, which is an A haplotype (based on KIR gene content; see Figure 1). SNPs that appear exclusively on B haplotypes are therefore excluded from the design of the array. Given the substantial structural variation, especially among B haplotypes, such SNPs would presumably be highly informative of B haplotype variation and would be valuable for inclusion in the model if they could be typed.

KIR*IMP was designed explicitly to work with SNP alleles. This was for simplicity and generality (see Uses for Our Method above). We have observed that extra information is available in the SNP-intensity measurements, including CNV in the SNPs themselves, which can be exploited for increasing accuracy (see Appendix C and Figure S11).

Table 2. **Most Informative SNPs**

SNP ID	rsID	Position	Allele 1	Allele 2	Gene	Gene Location	In the NG Validation Panel?	Poor Clustering?
rs587560	rs587560	55,245,738	A	G	*KIR3DL3*	intronic	yes	no
rs17207383	rs17207383	55,248,107	A	C	–	intergenic	no	no
seq-rs10409751	rs10409751	55,249,570	C	G	–	intergenic	no	no
seq-rs643236	rs643236	55,251,418	A	G	*KIR2DL3*	intronic	no	no
seq-t1d-19-59977961-T-C	–	55,286,149	A	G	*KIR2DL1*	intronic	no	yes
imm_19_59986266	–	55,294,454	A	G	*KIR2DL1*	coding	no	yes
seq-rs670795	rs670795	55,306,645	A	C	–	intergenic	yes	no
seq-rs35656676	rs35656676	55,314,897	C	G	–	intergenic	yes	no
seq-rs17173106	rs17173106	55,314,949	A	G	–	intergenic	yes	no
seq-rs592645	rs592645	55,320,927	A	T	*KIR2DL4*	intronic	yes	no
seq-rs3865510	rs3865510	55,324,239	A	C	*KIR2DL4*	intronic	yes	no
rs581623	rs581623	55,326,739	A	G	–	intergenic	yes	no
seq-t1d-19-60034052-C-T	–	55,342,240	A	G	–	intergenic	no	no
rs4806585	rs4806585	55,346,424	A	C	*KIR2DS4*	intronic	no	no
seq-rs62122181	rs62122181	55,347,366	A	G	*KIR2DS4*	intronic	no	yes
seq-t1d-19-60056605-A-T	–	55,364,793	A	T	*KIR3DL2*	intronic	no	yes

The set of SNPs selected as being the most informative for KIR imputation. All of these SNPs were in the UK reference panel, but only a subset (as indicated) were also present in the NG validation panel. SNPs with poor clustering (as indicated; see Figure S6) were excluded from the training set for all analyses unless otherwise stated. The SNP and gene information is from the annotation provided with the Illumina Immunochip array.

In addition to showing CNV, KIR genes also exhibit substantial allelic variation, especially among the A haplotypes.[14,34] Indeed, the variation within our SNP data is consistent with this fact (see Appendix B and Figure S12). We had available only CNV data and thus developed KIR*IMP specifically for this. Nevertheless, we expect that our method can be easily extended to imputing allelic variation as more data become available.

Appendix A: KIRs, Genes, and Nomenclature

We follow the KIR gene nomenclature approved by the HUGO Genome Nomenclature Committee (HGNC)[35] and available online (see Web Resources). A full list, including MIM references, is shown in Table S1.

In brief, the names represent the protein structures encoded by the genes and are organized as follows:

- Start with the acronym *KIR*.
- Add a single digit corresponding to the number of immunoglobulin (Ig) domains in the molecule, followed by the letter *D* (denoting "domain").
- Add one of the letters *L*, *S*, or *P* to signify a long cytoplasmic tail, short cytoplasmic tail, or pseudogene, respectively.
- Add a final digit to distinguish between genes encoding the same protein structure (e.g., *KIR2DL1*, *KIR2DL2*, and *KIR2DL3* all encode proteins with two Ig domains and a long cytoplasmic tail).

In addition to the above, the standard nomenclature distinguishes between two different versions of *KIR2DL5*, namely *KIR2DL5A* and *KIR2DL5B*. These can alternatively be referred to as *KIR2DL5T* and *KIR2DL5C* for the telomeric and centromeric versions, respectively.[9]
We deviate slightly from the above as follows (and as is shown in Table S1):

- We do not distinguish between the two types of *KIR2DL5*. Instead, we measured the total copy number of both versions and thus refer to them together as *KIR2DL5*.
- We measured copy number of *KIR3DL1* by using two different assays, which targeted two different exons of the gene: exon 4 and exon 9 (see Jiang et al.[9] for details). We report each of these separately and refer to them as *KIR3DL1ex4* and *KIR3DL1ex9* for exons 4 and 9, respectively.
- We distinguish between different versions of *KIR2DS4*: one with a 22-bp frameshift deletion and the (full-length) wild-type form. We measured the copy number of each by using separate assays and refer to them as *KIR2DS4DEL* and *KIR2DS4WT* for the deletion and the WT, respectively. In addition, we used a third assay to detect both forms of *KIR2DS4*; we refer to this as *KIR2DS4TOTAL*. See Jiang et al.[9] for details of the assays.

The distinction between genes and alleles is somewhat blurred in the KIR region because of intensive non-allelic

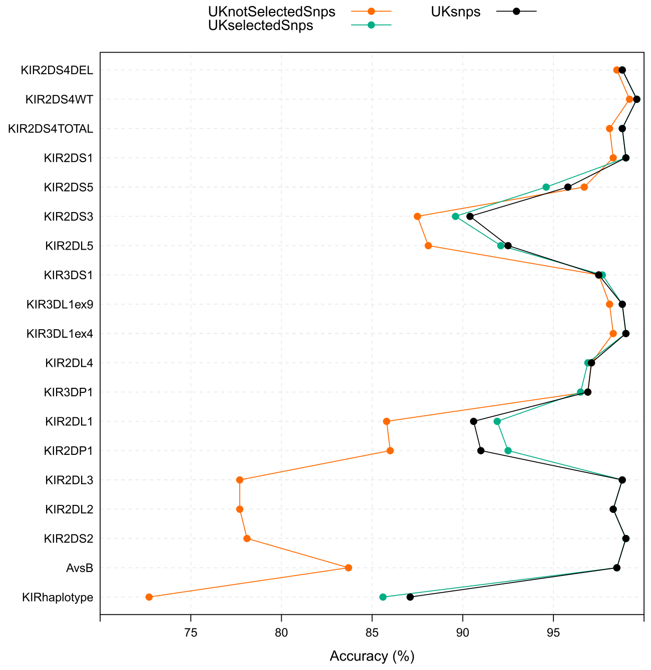

Figure 5. Imputation Accuracy with Different SNP Sets
Estimates of the KIR*IMP imputation accuracy from the UK cross-validation analysis are compared across different SNP subsets for training: the main set of SNPs used for the cross-validation analyses (*UKsnps*), the SNPs selected as being highly informative (*UKselectedSnps*), and the remaining set of SNPs (*UKnotSelectedSnps*).

homologous recombination. For example, *KIR3DL1* and *KIR3DS1* are now known to be alleles of the same gene; *KIR2DL2* and *KIR2DL3* are as well.[36] Therefore, for convenience we use the term "loci" to collectively refer to all of these. Table S1 clarifies the relationships between any specific entities of interest.

The KIR region is highly variable in terms of gene arrangement and copy number (see Figure 1) in that haplotypes can comprise 4 to 20 KIR genes.[9] The two classes of haplotypes are designated A and B. The A haplotypes are relatively stable in copy number, although the genes contained in A haplotypes can have considerable allelic sequence variation. B haplotypes show extensive CNV and also many different gene arrangements. Common haplotypes are typically described in terms of pairs of motifs on either side of a recombination hotspot. Each motif is made up of several KIR genes, typically a pair of "framework" genes at either end of the motif and combinations of other KIR genes in between. These motifs are distinguished according to whether they are on the centromeric or the telomeric side of the hotspot and are labeled accordingly with "c" or "t," respectively (e.g., cA01, cA02, tA01, tB01, etc.). Thus, common KIR haplotypes are defined, for example, as cA01-tA01, cA01-tB01, cB02-tA01, etc., where each motif is actually a haplotype made up of specific KIR genes (e.g., cB02 = 3DL3, 2DS2, 2DL2, and 3DP1; see Figure 1). The less common haplotypes have gene duplications, deletions, or fusions and are more complex. Non-allelic homologous recombination is the likely mechanism for much of the haplotype variation observed.

KIRs are members of the immunoglobulin superfamily of molecules and are present on NK cells and some T cells. Several KIRs are known to interact with HLA class I molecules. KIRs with two Ig domains are generally, but not exclusively, receptors for subsets of HLA-C molecules, whereas some with three Ig domains, such as KIR3DL1, interact with a subset of HLA-B and HLA-A allotypes. Some KIRs encode inhibitory immunoreceptor tyrosine-based inhibition motifs (ITIMs) in their cytoplasmic tails, whereas other KIRs (which are, interestingly, closely related to the former) are activating rather than inhibitory, given that they are coupled with adaptors with immunoreceptor tyrosine-based activation motifs (ITAMs). The A haplotypes contain mostly inhibitory KIRs, whereas the B haplotypes usually contain one or more activating KIRs.[9]

Appendix B: Structure of the KIR Region and Allelic Variation

The dense SNP data we have available, together with the modeling of KIR types, provide some further insight into the region containing the KIR genes. Figure S12 shows a visualization of the haplotypes observed in the SNP data (55.23–55.43 Mb). The KIR haplotypes are grouped to emphasize their similarities, from which we can also see the extent of diversity.

One clear observation is that the haplotypes differ in their diversity. For example, KIR haplotype 9 shows a distinct "barcode" pattern, such that all haplotypes within the group have a similar pattern of SNP alleles, suggesting a relative lack of allelic variation. In fact, haplotypes 7–9 all show the same barcode pattern, suggesting that they share a (single) common haplotype background. In contrast, haplotype 1 shows much more diversity in that it lacks any single barcode pattern. This suggests the presence of multiple haplotype backgrounds, possibly reflecting extensive allelic variation.

The KIR haplotypes that show evidence of multiple haplotype backgrounds (via the absence of a single barcode pattern) correspond exactly to the A/B classification of the haplotypes' centromeric and telomeric motifs (as described by Jiang et al.[9] and shown in Figure 1).

For the centromeric region, a small block of SNPs surrounding *KIR2DL3* and *KIR2DL1* distinguish two types of patterns, as described in Table B1. These are consistent with previous evidence showing that A haplotypes have greater allelic diversity than B haplotypes.[37]

Table B1. Patterns in the Centromeric KIR Region

Haplotypes	Motif(s)	Apparent Diversity
1, 2, 3, 11	cA01	high
4–10	cB01, cB02	low

For the telomeric region, a large block of SNPs in the center of the figure (surrounding *KIR2DL4–KIR3DL2*) distinguish three types of patterns, as described in Table B2.

Table B2. Patterns in the Telomeric KIR Region

Haplotypes	Motif	Apparent Diversity
1, 4, 6	tA01 with 2DS4v	high
2, 5, 10	tA01 with 2DS4f	low
3, 7–9, 11	tB01	low

As before, the first and third of these groups are consistent with previous evidence showing that A haplotypes have greater allelic diversity than B haplotypes. Interestingly, the second group is an A motif, but it has low diversity (although some more diversity seems to be present toward the telomeric end of the region, where the barcode pattern starts to become less apparent).

The two versions of tA01 differ in the version of *KIR2DS4* they carry. The first group above has the frameshift deletion variant (2DS4v), which disables that gene, whereas the second has the full-length form (2DS4f), which is functional. The latter has been shown to have a very specific role, which arose before separation of the human and chimpanzee lineages and which has subsequently been conserved and selected.[38] This could lead to low diversity for this variant, which is consistent with our observations here. In contrast, the non-functional version is expected to drift and lead to higher diversity, which is also consistent with our observations.

Appendix C: CNV of SNPs and SNP-Intensity Measurements

One potential source of useful information that we are not currently using is CNV within the SNPs themselves. KIR*IMP relies on finding combinations of SNP alleles that differentiate the KIR copy-number types. We have seen evidence in the raw SNP-intensity measurements that some SNPs are copy-number variable and most likely directly capture KIR CNV (data not shown). On the whole, such signals are much less distinct than standard SNP allele clusters, and it will be challenging to incorporate both types of variation. A study that has done this is by Pontikos et al.,[15] who used a single SNP to infer copy number for one KIR gene (and other genes highly correlated with it). For simplicity and generality, we have chosen to limit ourselves to using only SNP alleles; however, this is certainly a promising avenue for improving the method.

To illustrate the potential gains of using the raw intensity measurements more directly, we compared the performance of KIR*IMP when the four SNPs identified as having poor clustering were included in the training set. The results are shown in Figure S11. Adding these SNPs boosted the accuracy for the five loci that are the hardest to impute: *KIR2DP1*, *KIR2DL1*, *KIR2DL5*, *KIR2DS3*, and *KIR2DS5*. These SNPs are indeed informative for these loci, given that the calls from the clusters fitted by the genotype-calling algorithm captured some of this information. It appears that much of this is due to noisy SNP alleles, but some of it might also be due to CNV. In any case, the clusters do not necessarily correspond to a pattern of variation we can confidently say will be consistently called across datasets (such as true underlying SNP genotypes); hence, we have chosen to exclude them. Nevertheless, these results suggest that there is scope for improving the accuracy of imputation if this information can be distilled in a more reliable way.

Supplemental Data

Supplemental Data include 12 figures and 5 tables and can be found with this article online at http://dx.doi.org/10.1016/j.ajhg.2015.09.005.

Acknowledgments

We wish to thank Chris Johnson, Wei Jiang, and Jyothi Jayaraman for their contributions to developing the lab-based KIR typing process and data generation, Sandra Maksimovic for assisting with the web-based implementation, Bente Woldseth for technical assistance, and Johannes Hov for helpful discussions. This work was supported by Australian National Health and Medical Research Council (NHMRC) Career Development Fellowship ID 1053756 (S.L.), Victorian Life Sciences Computation Initiative (VLSCI) grant VR0240 on its Peak Computing Facility at the University of Melbourne, an initiative of the Victorian Government of Australia (S.L.), UK Multiple Sclerosis Society grant 894/08 (S.S.), the German Ministry of Education and Research (BMBF) program e:Med sysINFLAME (A.F.), the Peter Hans Hofschneider Professorship of the "Stiftung Experimentelle Biomedizin" in Zürich (A.F.), and the Wellcome Trust and Medical Research Council (MRC) with partial funding from the National Institute of Health Cambridge Biomedical Research Centre (J.T. and J.A.T.). Research at the Murdoch Childrens Research Institute was supported by the Victorian Government's Operational Infrastructure Support Program, and further infrastructure support was provided by the Deutsche Forschungsgemeinschaft (DFG) Cluster of Excellence "Inflammation at Interfaces." S.L., G.M., and A.D. are founders and partners in Peptide Groove LLP.

Received: April 20, 2015
Accepted: September 8, 2015
Published: October 1, 2015

Web Resources

The URLs for data presented herein are as follows:

Affymetrix, http://www.affymetrix.com/

Genome Reference Consortium GRCh37, https://www.ncbi.nlm.nih.gov/projects/genome/assembly/grc/human/

Illumina, http://www.illumina.com/

KIR gene nomenclature, https://www.ebi.ac.uk/ipd/kir/genes.html
KIR*IMP, http://imp.mcri.edu.au/
OMIM, http://www.omim.org/
SHAPEIT: HapMap phase II b37 recombination map, https://mathgen.stats.ox.ac.uk/genetics_software/shapeit/shapeit.html#gmap

References

1. Leslie, S., Donnelly, P., and McVean, G. (2008). A statistical method for predicting classical HLA alleles from SNP data. Am. J. Hum. Genet. *82*, 48–56.
2. Dilthey, A.T., Moutsianas, L., Leslie, S., and McVean, G. (2011). HLA*IMP–an integrated framework for imputing classical HLA alleles from SNP genotypes. Bioinformatics *27*, 968–972.
3. Dilthey, A., Leslie, S., Moutsianas, L., Shen, J., Cox, C., Nelson, M.R., and McVean, G. (2013). Multi-population classical HLA type imputation. PLoS Comput. Biol. *9*, e1002877.
4. Jia, X., Han, B., Onengut-Gumuscu, S., Chen, W.-M., Concannon, P.J., Rich, S.S., Raychaudhuri, S., and de Bakker, P.I.W. (2013). Imputing amino acid polymorphisms in human leukocyte antigens. PLoS ONE *8*, e64683.
5. Zheng, X., Shen, J., Cox, C., Wakefield, J.C., Ehm, M.G., Nelson, M.R., and Weir, B.S. (2014). HIBAG–HLA genotype imputation with attribute bagging. Pharmacogenomics J. *14*, 192–200.
6. Strange, A., Capon, F., Spencer, C.C., Knight, J., Weale, M.E., Allen, M.H., Barton, A., Band, G., Bellenguez, C., Bergboer, J.G., et al.; Genetic Analysis of Psoriasis Consortium & the Wellcome Trust Case Control Consortium 2 (2010). A genome-wide association study identifies new psoriasis susceptibility loci and an interaction between HLA-C and ERAP1. Nat. Genet. *42*, 985–990.
7. Evans, D.M., Spencer, C.C., Pointon, J.J., Su, Z., Harvey, D., Kochan, G., Oppermann, U., Dilthey, A., Pirinen, M., Stone, M.A., et al.; Spondyloarthritis Research Consortium of Canada (SPARCC); Australo-Anglo-American Spondyloarthritis Consortium (TASC); Wellcome Trust Case Control Consortium 2 (WTCCC2) (2011). Interaction between ERAP1 and HLA-B27 in ankylosing spondylitis implicates peptide handling in the mechanism for HLA-B27 in disease susceptibility. Nat. Genet. *43*, 761–767.
8. Sawcer, S., Hellenthal, G., Pirinen, M., Spencer, C.C., Patsopoulos, N.A., Moutsianas, L., Dilthey, A., Su, Z., Freeman, C., Hunt, S.E., et al.; International Multiple Sclerosis Genetics Consortium; Wellcome Trust Case Control Consortium 2 (2011). Genetic risk and a primary role for cell-mediated immune mechanisms in multiple sclerosis. Nature *476*, 214–219.
9. Jiang, W., Johnson, C., Jayaraman, J., Simecek, N., Noble, J., Moffatt, M.F., Cookson, W.O., Trowsdale, J., and Traherne, J.A. (2012). Copy number variation leads to considerable diversity for B but not A haplotypes of the human KIR genes encoding NK cell receptors. Genome Res. *22*, 1845–1854.
10. Kulkarni, S., Martin, M.P., and Carrington, M. (2008). The Yin and Yang of HLA and KIR in human disease. Semin. Immunol. *20*, 343–352.
11. Khakoo, S.I., and Carrington, M. (2006). KIR and disease: a model system or system of models? Immunol. Rev. *214*, 186–201.
12. Parham, P., and Moffett, A. (2013). Variable NK cell receptors and their MHC class I ligands in immunity, reproduction and human evolution. Nat. Rev. Immunol. *13*, 133–144.
13. Velardi, A. (2008). Role of KIRs and KIR ligands in hematopoietic transplantation. Curr. Opin. Immunol. *20*, 581–587.
14. Norman, P.J., Hollenbach, J.A., Nemat-Gorgani, N., Guethlein, L.A., Hilton, H.G., Pando, M.J., Koram, K.A., Riley, E.M., Abi-Rached, L., and Parham, P. (2013). Co-evolution of human leukocyte antigen (HLA) class I ligands with killer-cell immunoglobulin-like receptors (KIR) in a genetically diverse population of sub-Saharan Africans. PLoS Genet. *9*, e1003938.
15. Pontikos, N., Smyth, D.J., Schuilenburg, H., Howson, J.M., Walker, N.M., Burren, O.S., Guo, H., Onengut-Gumuscu, S., Chen, W.-M., Concannon, P., et al. (2014). A hybrid qPCR/SNP array approach allows cost efficient assessment of KIR gene copy numbers in large samples. BMC Genomics *15*, 274.
16. Roberts, C.H., Jiang, W., Jayaraman, J., Trowsdale, J., Holland, M.J., and Traherne, J.A. (2014). Killer-cell Immunoglobulin-like Receptor gene linkage and copy number variation analysis by droplet digital PCR. Genome Med. *6*, 20.
17. Liu, J.Z., Hov, J.R., Folseraas, T., Ellinghaus, E., Rushbrook, S.M., Doncheva, N.T., Andreassen, O.A., Weersma, R.K., Weismüller, T.J., Eksteen, B., et al.; UK-PSCSC Consortium; International IBD Genetics Consortium; International PSC Study Group (2013). Dense genotyping of immune-related disease regions identifies nine new risk loci for primary sclerosing cholangitis. Nat. Genet. *45*, 670–675.
18. Cortes, A., and Brown, M.A. (2011). Promise and pitfalls of the Immunochip. Arthritis Res. Ther. *13*, 101.
19. Shah, T.S., Liu, J.Z., Floyd, J.A., Morris, J.A., Wirth, N., Barrett, J.C., and Anderson, C.A. (2012). optiCall: a robust genotype-calling algorithm for rare, low-frequency and common variants. Bioinformatics *28*, 1598–1603.
20. Delaneau, O., Marchini, J., and Zagury, J.-F. (2012). A linear complexity phasing method for thousands of genomes. Nat. Methods *9*, 179–181.
21. Delaneau, O., Zagury, J.-F., and Marchini, J. (2013). Improved whole-chromosome phasing for disease and population genetic studies. Nat. Methods *10*, 5–6.
22. O'Connell, J., Gurdasani, D., Delaneau, O., Pirastu, N., Ulivi, S., Cocca, M., Traglia, M., Huang, J., Huffman, J.E., Rudan, I., et al. (2014). A general approach for haplotype phasing across the full spectrum of relatedness. PLoS Genet. *10*, e1004234.
23. Breiman, L. (2001). Random Forests. Mach. Learn. *45*, 5–32.
24. Breiman, L. (1996). Bagging predictors. Mach. Learn. *24*, 123–140.
25. Ho, T.K. (1998). The random subspace method for constructing decision forests. IEEE Trans. Pattern Anal. Mach. Intell. *20*, 832–844.
26. Bryll, R., Gutierrez-Osuna, R., and Quek, F. (2003). Attribute bagging: improving accuracy of classifier ensembles by using random feature subsets. Pattern Recognit. *36*, 1291–1302.
27. Liaw, A., and Wiener, M. (2002). Classification and Regression by randomForest. R News *2*, 18–22.
28. Bengio, Y., and Grandvalet, Y. (2004). No Unbiased Estimator of the Variance of K-Fold Cross-Validation. J. Mach. Learn. Res. *5*, 1089–1105.
29. Holland, P.M., Abramson, R.D., Watson, R., and Gelfand, D.H. (1991). Detection of specific polymerase chain reaction product by utilizing the 5′——3′ exonuclease activity of Thermus aquaticus DNA polymerase. Proc. Natl. Acad. Sci. USA *88*, 7276–7280.
30. Spencer, C., Hechter, E., Vukcevic, D., and Donnelly, P. (2011). Quantifying the underestimation of relative risks from genome-wide association studies. PLoS Genet. *7*, e1001337.

31. Vukcevic, D., Hechter, E., Spencer, C., and Donnelly, P. (2011). Disease model distortion in association studies. Genet. Epidemiol. *35*, 278–290.

32. Hosking, F.J., Leslie, S., Dilthey, A., Moutsianas, L., Wang, Y., Dobbins, S.E., Papaemmanuil, E., Sheridan, E., Kinsey, S.E., Lightfoot, T., et al. (2011). MHC variation and risk of childhood B-cell precursor acute lymphoblastic leukemia. Blood *117*, 1633–1640.

33. Hollenbach, J.A., Nocedal, I., Ladner, M.B., Single, R.M., and Trachtenberg, E.A. (2012). Killer cell immunoglobulin-like receptor (KIR) gene content variation in the HGDP-CEPH populations. Immunogenetics *64*, 719–737.

34. Yawata, M., Yawata, N., Draghi, M., Little, A.-M., Partheniou, F., and Parham, P. (2006). Roles for HLA and KIR polymorphisms in natural killer cell repertoire selection and modulation of effector function. J. Exp. Med. *203*, 633–645.

35. Marsh, S.G.E., Parham, P., Dupont, B., Geraghty, D.E., Trowsdale, J., Middleton, D., Vilches, C., Carrington, M., Witt, C., Guethlein, L.A., et al. (2003). Killer-cell immunoglobulin-like receptor (KIR) nomenclature report, 2002. Eur. J. Immunogenet. *30*, 229–234.

36. Carrington, M., and Norman, P. (2003). The KIR Gene Cluster (National Center for Biotechnology Information).

37. Pyo, C.-W., Guethlein, L.A., Vu, Q., Wang, R., Abi-Rached, L., Norman, P.J., Marsh, S.G.E., Miller, J.S., Parham, P., and Geraghty, D.E. (2010). Different patterns of evolution in the centromeric and telomeric regions of group A and B haplotypes of the human killer cell Ig-like receptor locus. PLoS ONE *5*, e15115.

38. Graef, T., Moesta, A.K., Norman, P.J., Abi-Rached, L., Vago, L., Older Aguilar, A.M., Gleimer, M., Hammond, J.A., Guethlein, L.A., Bushnell, D.A., et al. (2009). KIR2DS4 is a product of gene conversion with KIR3DL2 that introduced specificity for HLA-A*11 while diminishing avidity for HLA-C. J. Exp. Med. *206*, 2557–2572.

The American Journal of Human Genetics
Supplemental Data

Imputation of KIR Types from SNP Variation Data

Damjan Vukcevic, James A. Traherne, Sigrid Næss, Eva Ellinghaus, Yoichiro Kamatani, Alexander Dilthey, Mark Lathrop, Tom H. Karlsen, Andre Franke, Miriam Moffatt, William Cookson, John Trowsdale, Gil McVean, Stephen Sawcer, and Stephen Leslie

Supplemental Figures

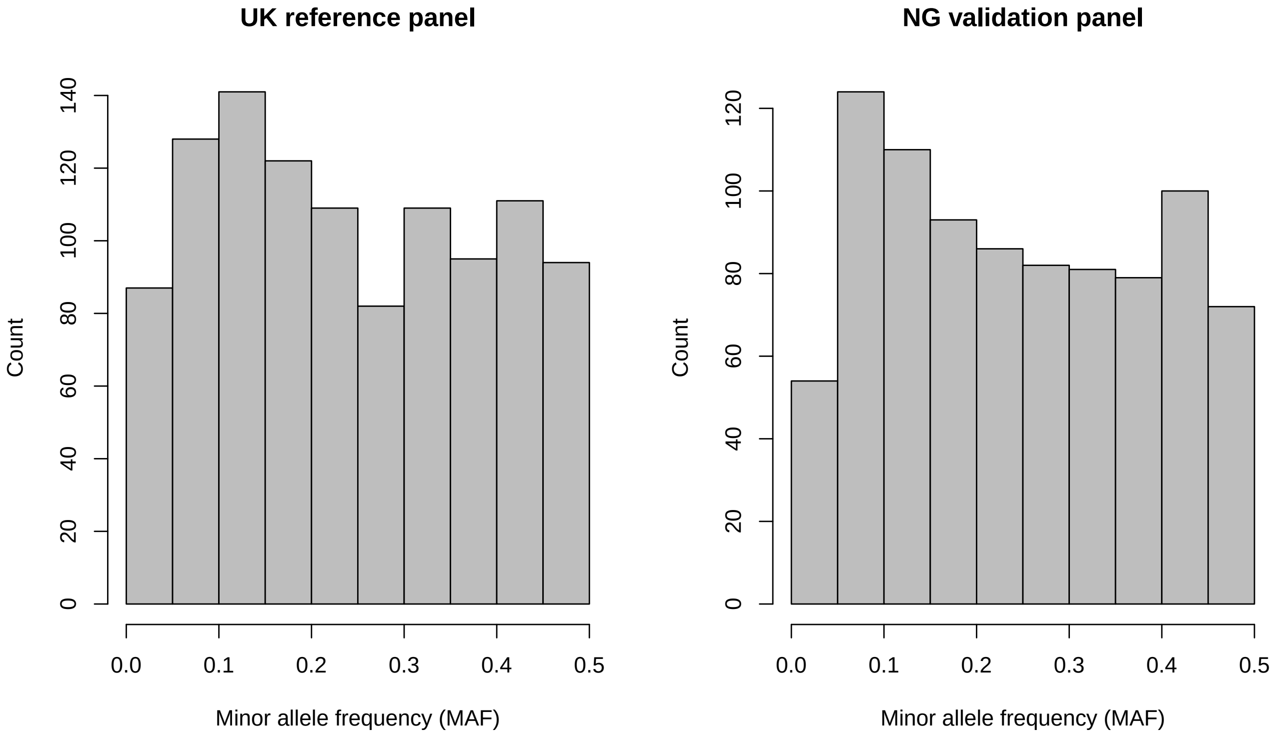

Figure S1. MAF histograms.
The distribution of SNP allele frequencies in each of the reference panels.

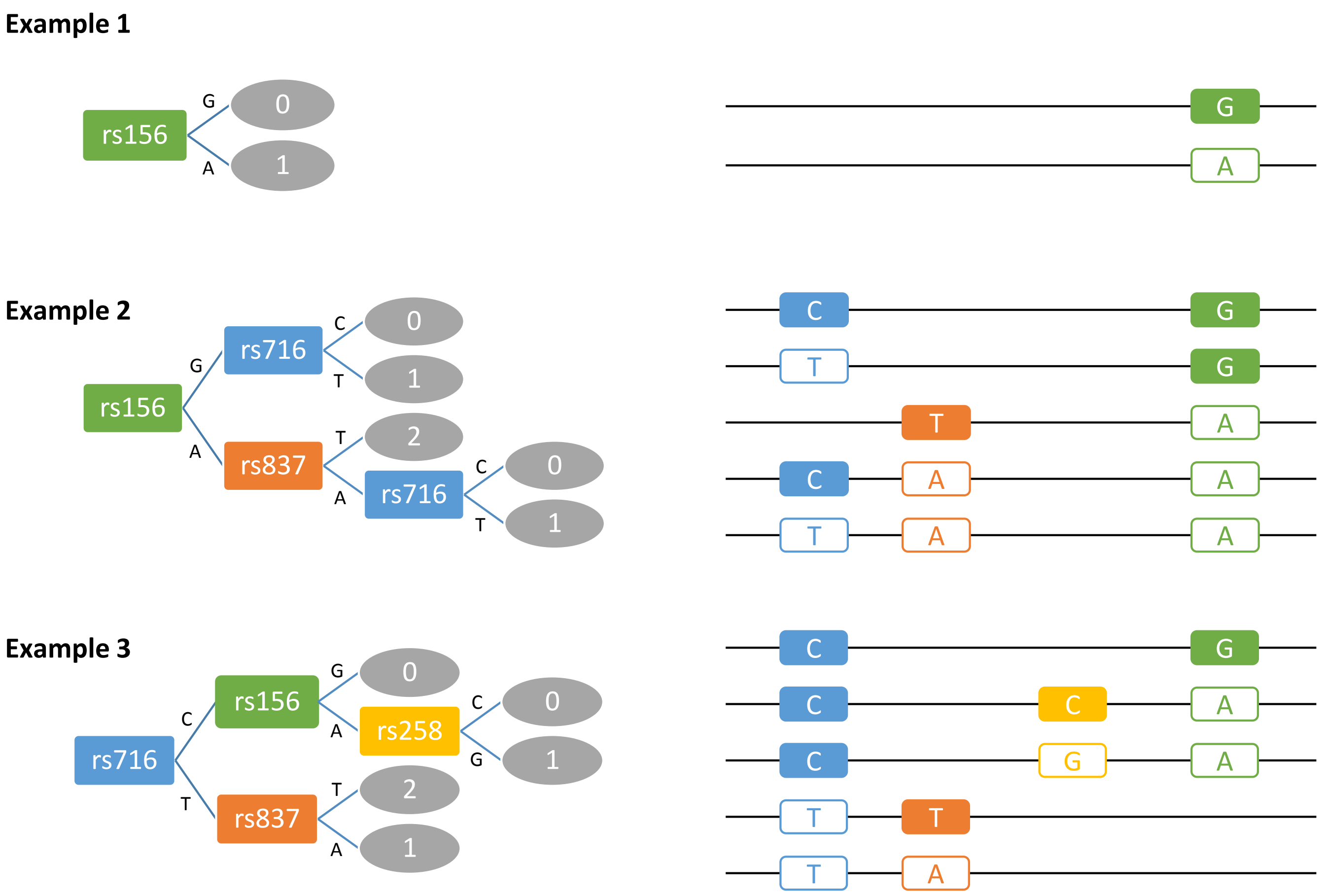

Figure S2. Genetic interpretation of classification trees.
A diagram showing how classification trees describe haplotype partitions. Three example trees are shown on the left and their corresponding haplotype partitions on the right. The coloured rectangular nodes in the trees represent SNPs and the branches leading from each one correspond to alleles at the SNP (as marked); the grey end nodes represent copy number types for the target locus. A path through a tree (from left to right) defines a haplotype, as a set of alleles at a set of SNPs (the ones that appear on that particular path). The haplotype diagrams show all of these haplotypes (one for each end node of each tree, i.e. for each possible path). Each SNP is depicted as a rectangle labelled with its allele, or is absent if that SNP is not on the path defining that haplotype. Note that a given SNP can appear more than once in the same tree (e.g. rs716 in Example 2). By construction, the set of haplotypes from a given tree forms a complete partition of the space of haplotypes.

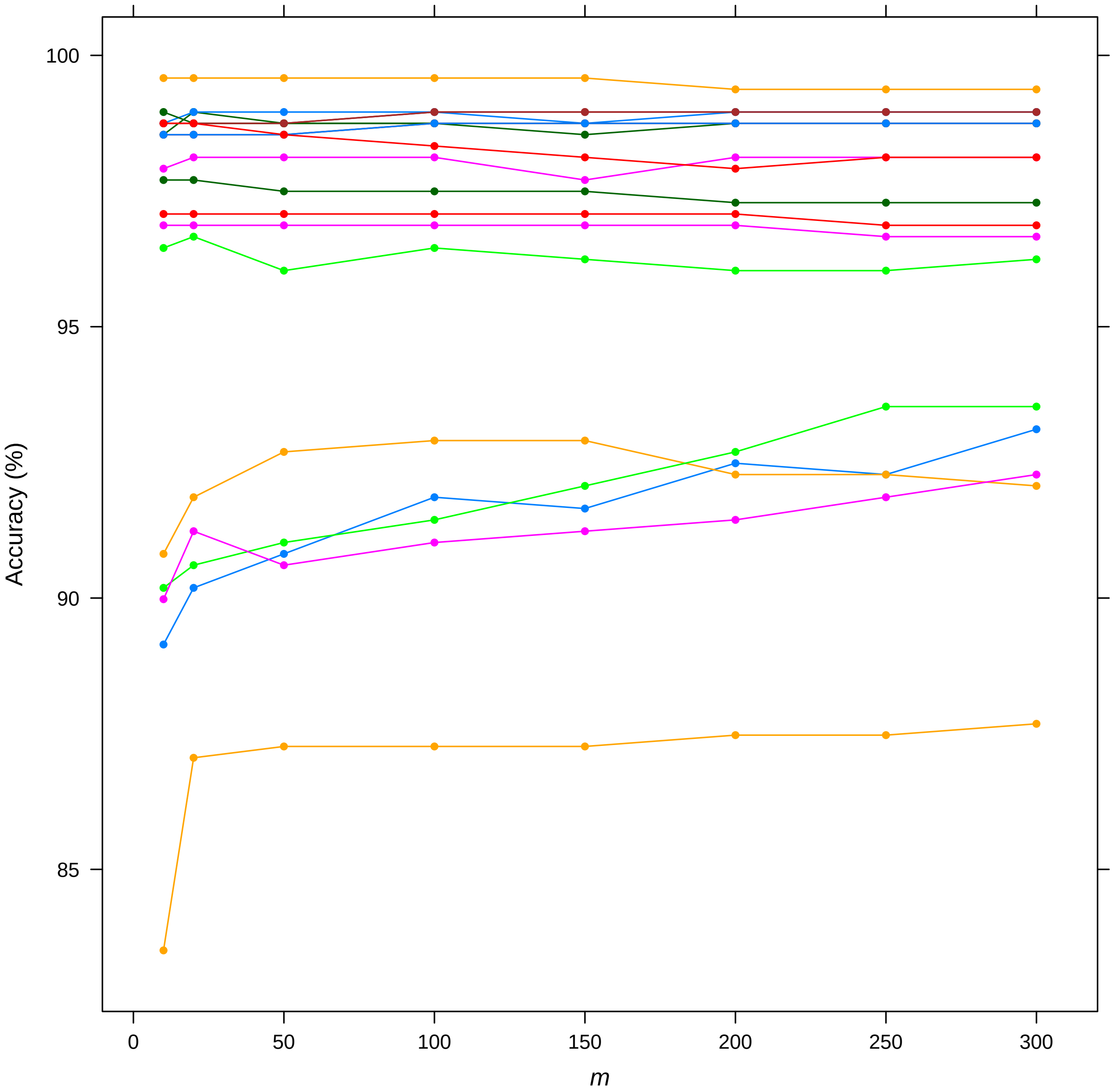

Figure S3. Parameter tuning.
Imputation accuracy estimates from the parameter tuning experiments. Each line shows the OOB accuracy for KIR*IMP for a particular KIR locus, trained with the UK reference panel with the *UKsnps* set, for various values of the key tuning parameter (m). The lines are coloured to distinguish them for visual convenience.

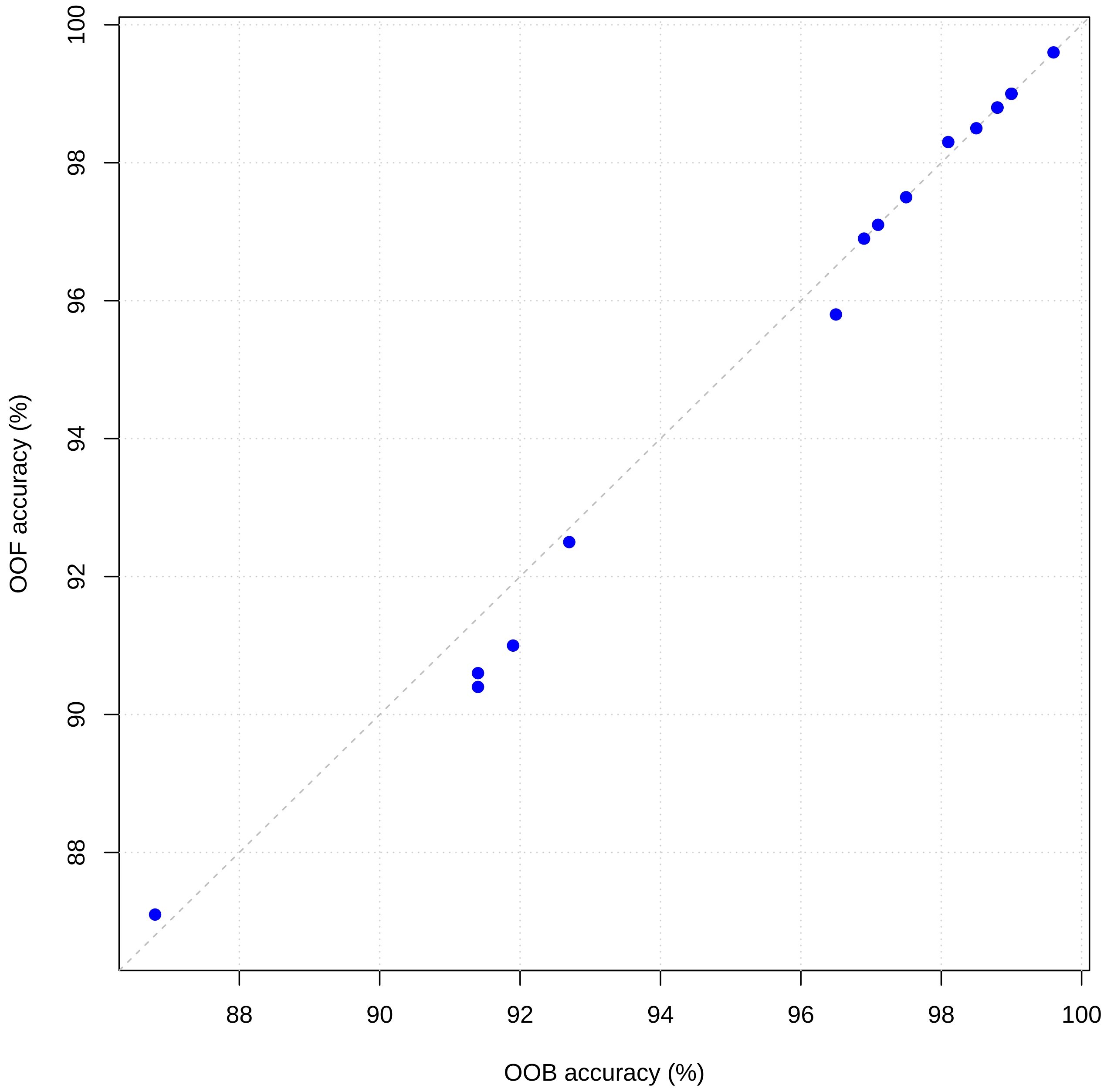

Figure S4. OOB vs cross-validation imputation accuracy.
A comparison of the out-of-bag (OOB) and the out-of-fold (OOF) estimates of imputation accuracy. The former were calculated during training with the full data, the latter as part of the UK cross-validation analysis. Each point corresponds to a different KIR locus. All results are for KIR*IMP trained with the UK reference panel with the *UKsnps* set.

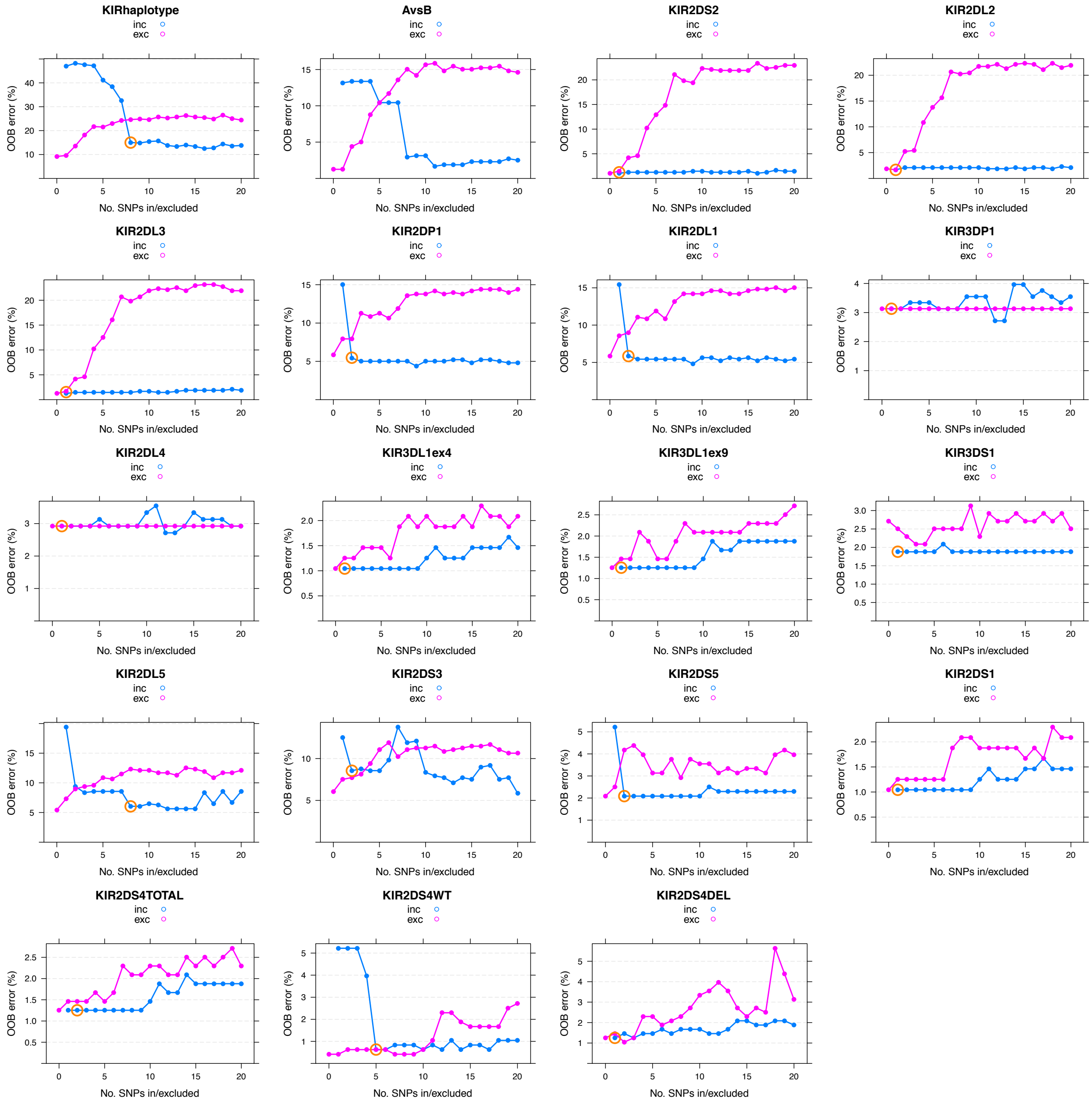

Figure S5. SNP selection experiments.
OOB imputation accuracy estimates from the SNP selection experiments. Each panel shows the results from a different locus, for models fitted with either just the selected SNPs (inc) or all of the remaining SNPs (exc). Points circled in orange indicate which SNPs were manually selected (from that locus) into the set of most informative SNPs (see Material and Methods and Table 2). All results are for KIR*IMP trained with the UK reference panel, starting with the *UKplus4snps* set of SNPs. Note that the y-axis scale varies for each panel.

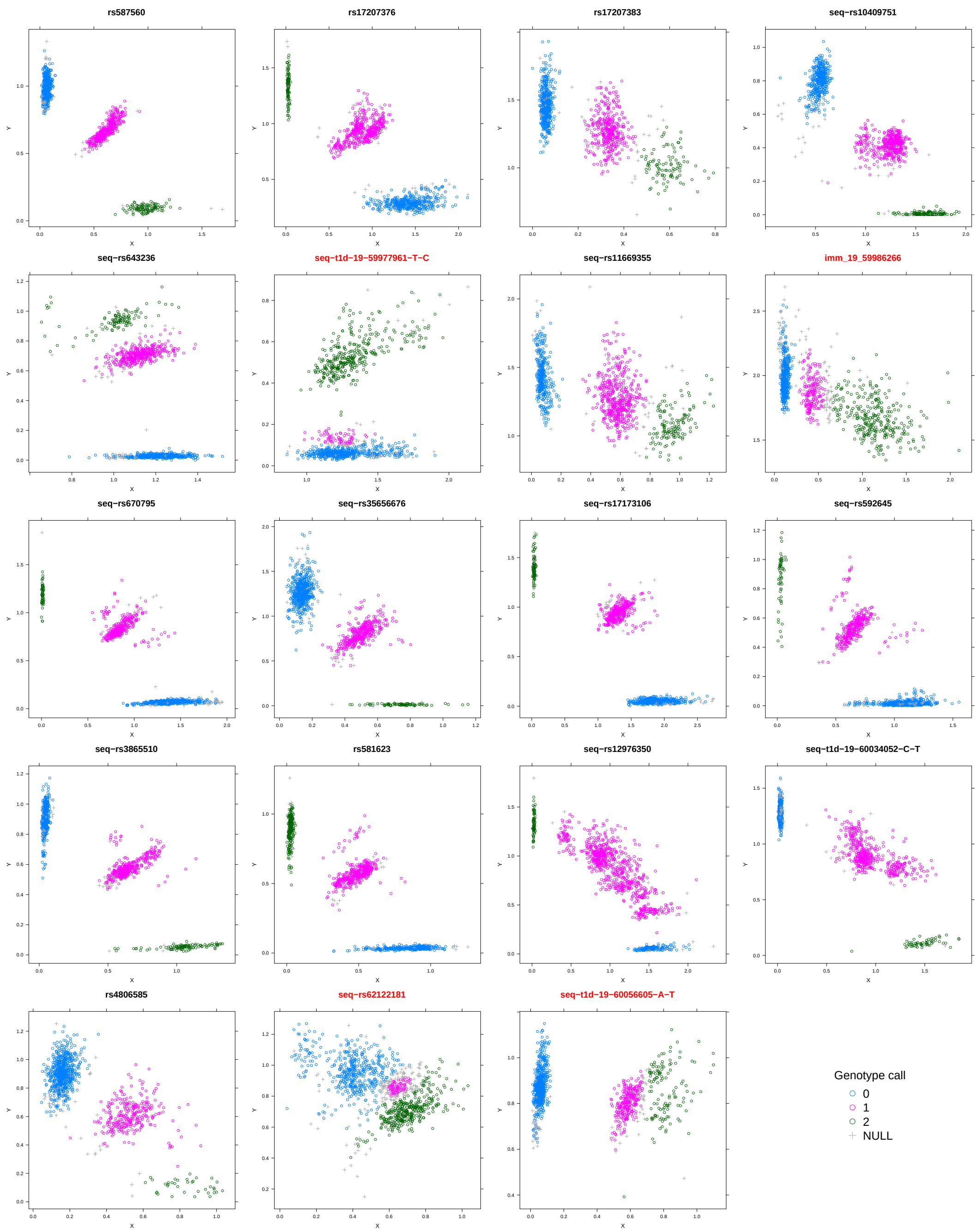

Figure S6. SNP genotyping diagnostic plots.
Scatter plots of the normalised SNP intensity measurements. Each point represents the measurements for a single individual for a given SNP. The shape and colour of each point correspond to the genotype call, as indicated by the legend in the bottom-right. Genotype calls are shown as an integer, with 1 being the heterozygote, and with NULL representing a missing call (i.e. did not meet the relevant quality criteria to be given a call). Each panel shows one of the 19 SNPs identified from the SNP selection experiments (identified by the title of each panel). Four of these were excluded due to poor clustering, these are indicated in Table 2 and shown here by a red title for the panel.

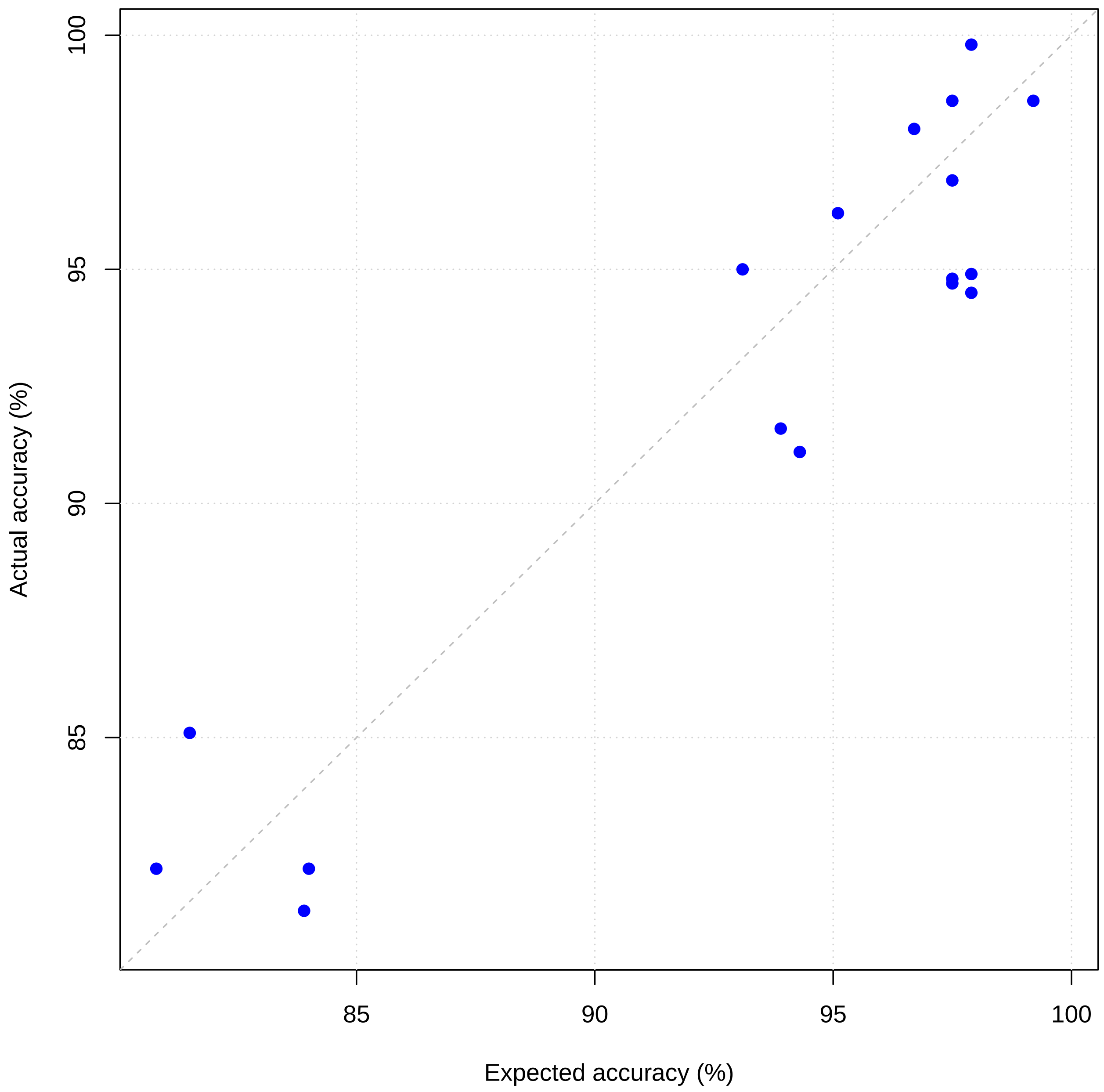

Figure S7. Per-haplotype vs per-individual imputation accuracy.
A comparison of the expected and actual imputation accuracy estimates for the NG validation analysis for KIR*IMP. The actual accuracy estimate is the percentage of individuals with correctly imputed copy number in the NG validation panel. The expected accuracy is calculated from the per-haplotype OOB accuracy estimates on the UK reference panel (obtained during model training), by assuming a model where a pair of haplotypes are chosen independently and uniformly at random and combined. Each point corresponds to a different copy-number KIR locus.

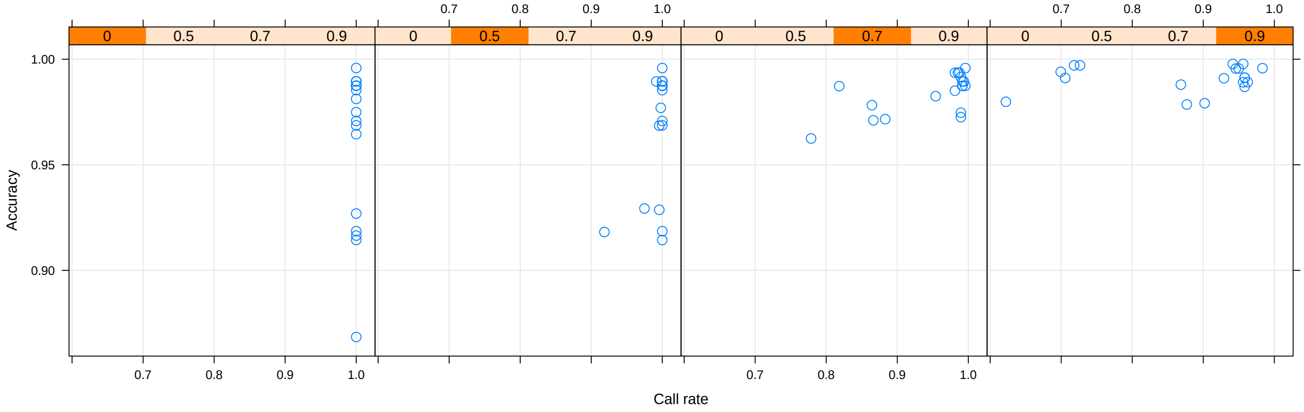

Figure S8. Imputation performance at various calling thresholds.
Scatter plots of OOB imputation accuracy against call rate. Each point corresponds to a different KIR locus and each panel to a different call threshold (as highlighted). All results are for KIR*IMP trained with the UK reference panel with the *UKsnps* set.

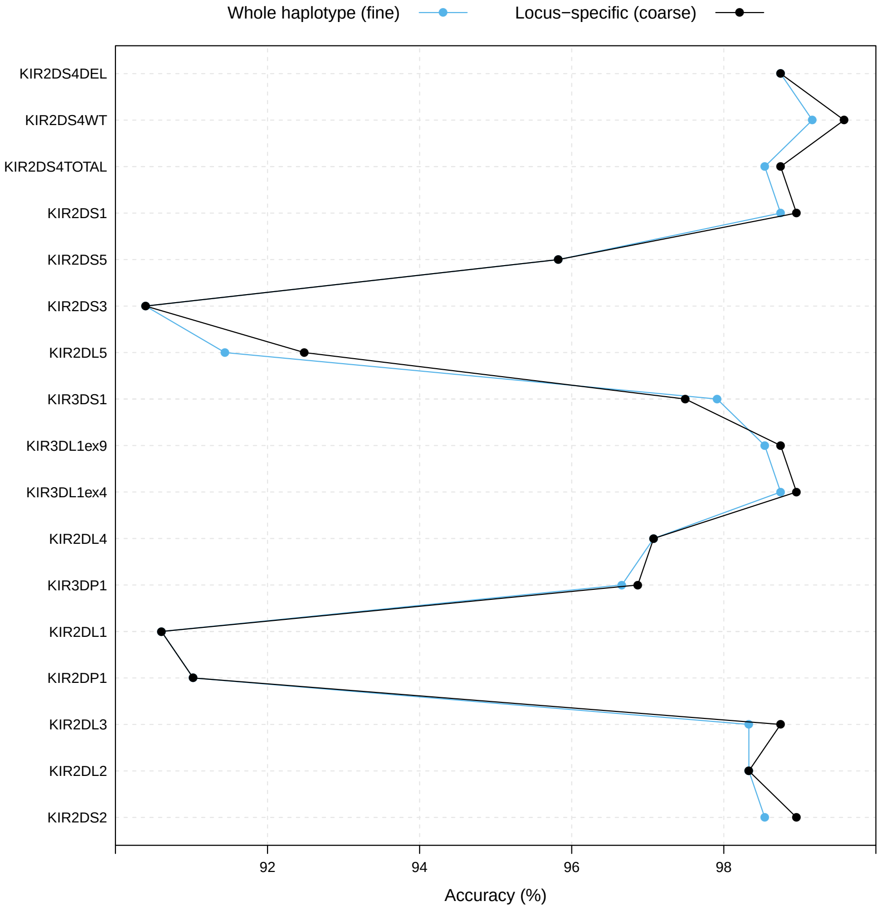

Figure S9. Imputation of fine-grained vs coarse-grained KIR types.
Imputation accuracy for models at different levels of granularity. A single model of the complete, fine-grained KIR haplotypes (coarsened to get calls for copy number for each constituent locus) is compared against separate (coarse-grained) locus-specific models. All results are from the UK cross-validation analysis of KIR*IMP trained with the *UKsnps* set.

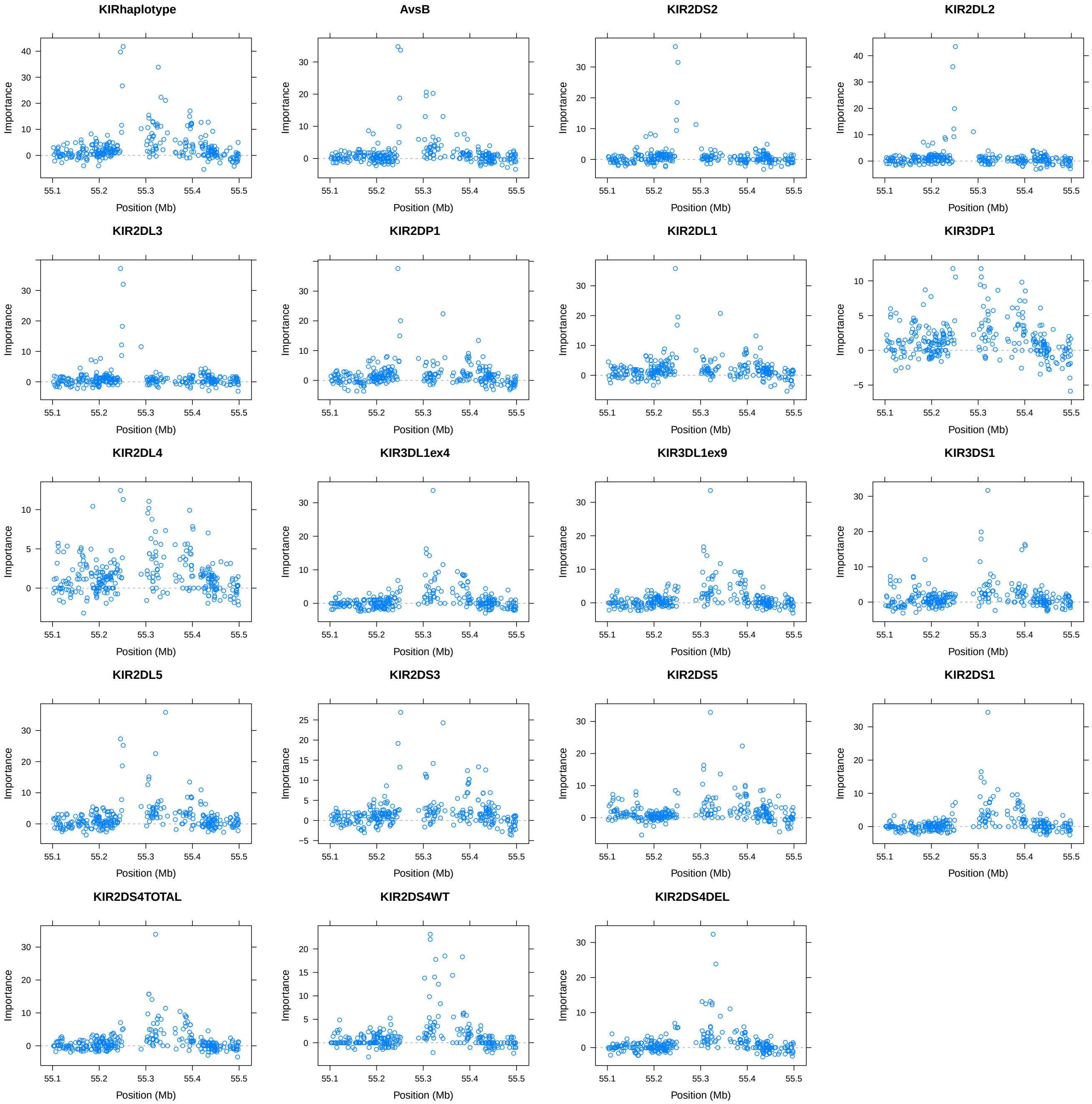

Figure S10. Variable importance plots.
Scatter plots of the variable importance score against genomic position for each SNP (see Material and Methods). Each panel shows the results from a different locus, for KIR*IMP trained with the UK reference panel with the *UKsnps* set.

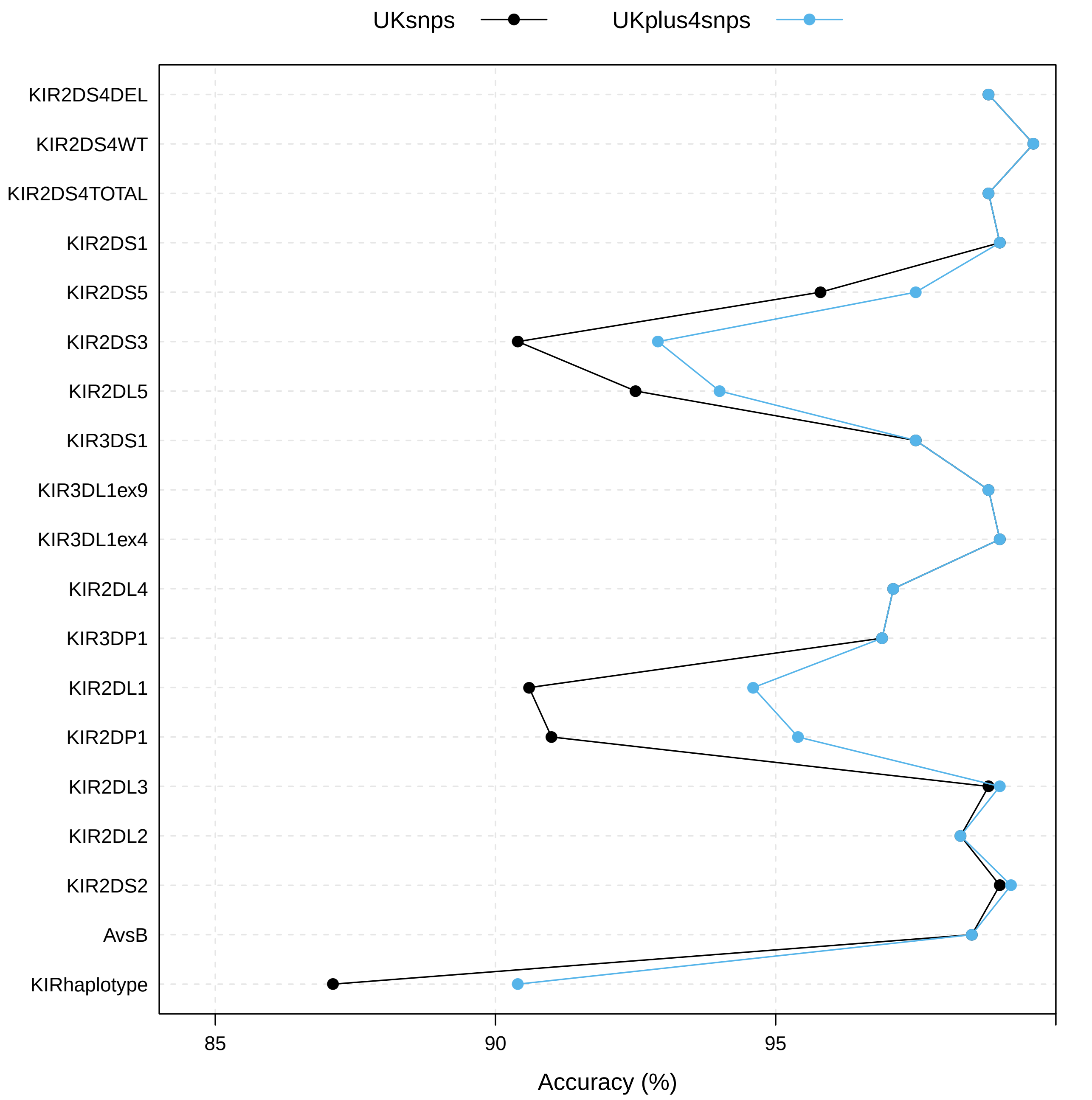

Figure S11. Imputation accuracy when including SNPs with poor clustering. Estimates of the KIR*IMP imputation accuracy from the UK cross-validation analysis, comparing two different SNP subsets for training: the main set of SNPs used for the cross-validation analyses (*UKsnps*) and the same set supplemented with the 4 SNPs identified as having poor clustering (*UKplus4snps*).

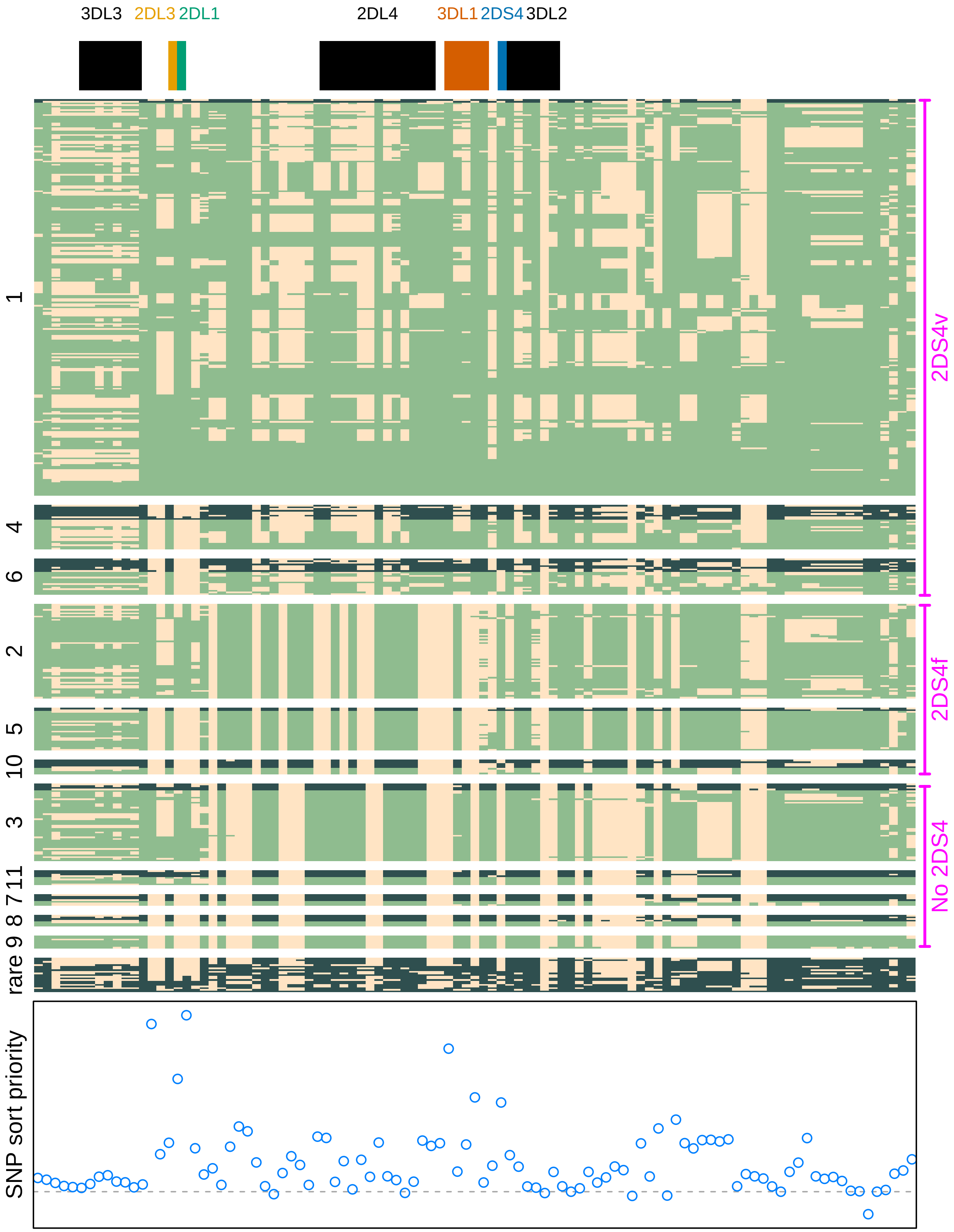

Figure S12. SNP haplotypes. (Continued on the following page)

Figure S12. SNP haplotypes. (cont'd)
A visualisation of the UK reference panel in the style of a heat map. All SNPs in the region 55.23–55.43 Mb are shown, excluding those that were monomorphic in the UK reference panel or had poor clustering. Rows correspond to haplotypes and columns to SNPs.Each cell is coloured either yellow or green (the latter can be either light or dark green, see below) depending on the allele at that SNP for that haplotype; these two colours are mapped arbitrarily to the two alleles at each SNP. Haplotypes are grouped by their KIR haplotype, as labelled on the left; the 11 most common KIR haplotypes are shown separately and the rest are grouped together ('rare'). Within each group, haplotypes are sorted in a lexicographical order based on the SNP alleles, taken in the order defined by their variable importance score from the KIR*IMP model fitted to *KIRhaplotype*; this score is plotted in the panel on the bottom. The haplotype groups are shown in a manually selected order to highlight common patterns between them, with the labels on the right indicating the presence or absence of particular versions of *KIR2DS4* (see Appendix B). SNPs are shown in annotated genomic position order (n.b. they are therefore not drawn to scale based on genomic position, like in Figure 1). Two shades of green are used: light green for haplotypes that are imputed correctly, dark green for those imputed incorrectly. The coloured bars at the top indicate SNPs that are annotated as being within a KIR gene, using the same colour scheme as Figure 1.

Supplemental Tables

Table S1. KIR loci.

Gene			
**Current knowledge**	**HGNC**	**Assay identifier**	**MIM**
KIR3DL3	*KIR3DL3*	*KIR3DL3*	610095
KIR2DS2	*KIR2DS2*	*KIR2DS2*	604953
*KIR2DL2/L3**	*KIR2DL2*	*KIR2DL2*	604937
	KIR2DL3	*KIR2DL3*	604938
KIR2DP1	*KIR2DP1*	*KIR2DP1*	—
KIR2DL1	*KIR2DL1*	*KIR2DL1*	604936
KIR3DP1	*KIR3DP1*	*KIR3DP1*	610604
KIR2DL4	*KIR2DL4*	*KIR2DL4*	604945
*KIR3DL1/S1**	*KIR3DL1*	*KIR3DL1ex4*	604946
		KIR3DL1ex9	
	KIR3DS1	*KIR3DS1*	
KIR2DL5A	*KIR2DL5A*	*KIR2DL5*	605305
KIR2DL5B	*KIR2DL5B*		615727
KIR2DS3	*KIR2DS3*	*KIR2DS3*	604954
KIR2DS5	*KIR2DS5*	*KIR2DS5*	604956
KIR2DS1	*KIR2DS1*	*KIR2DS1*	604952
KIR2DS4	*KIR2DS4*	*KIR2DS4TOTAL*	604955
		KIR2DS4WT	
		KIR2DS4DEL	
KIR3DL2	*KIR3DL2*	*KIR3DL2*	604947

The relationship between KIR genes (and pseudogenes) and our copy number assays. We show both the set of genes defined by the HUGO Genome Nomenclature Committee (HGNC) and also a revised set ('Current knowledge') where two pairs of genes have been merged (marked by an asterisk) reflecting more recent research showing that these are actually different alleles of the same gene.[1] The MIM number for each gene is also provided; see MIM 604936 for general information about the KIR gene family.

Table S2. Definitions of KIR haplotypes.

KIRhaplotype	AvsB	KIR3DL3	KIR2DS2	KIR2DL2	KIR2DL3	KIR2DP1	KIR2DL1	KIR3DP1	KIR2DL4	KIR3DL1ex4	KIR3DL1ex9	KIR3DS1	KIR2DL5	KIR2DS3	KIR2DS5	KIR2DS1	KIR2DS4TOTAL	KIR2DS4WT	KIR2DS4DEL	KIR3DL2
1	A	1	0	0	1	1	1	1	1	1	1	0	0	0	0	0	1	0	1	1
2	A	1	0	0	1	1	1	1	1	1	1	0	0	0	0	0	1	1	0	1
3	B	1	0	0	1	1	1	1	1	0	0	1	1	0	1	1	0	0	0	1
4	B	1	1	1	0	0	0	1	1	1	1	0	0	0	0	0	1	0	1	1
5	B	1	1	1	0	0	0	1	1	1	1	0	0	0	0	0	1	1	0	1
6	B	1	1	1	0	1	1	1	1	1	1	0	1	1	0	0	1	0	1	1
7	B	1	1	1	0	1	1	1	1	0	0	1	2	1	1	1	0	0	0	1
8	B	1	1	1	0	0	0	1	1	0	0	1	1	0	1	1	0	0	0	1
9	B	1	1	1	0	1	1	1	1	0	0	1	2	2	0	1	0	0	0	1
10	B	1	1	1	0	1	1	1	1	1	1	0	1	1	0	0	1	1	0	1
11	B	1	0	0	1	1	1	1	1	0	0	1	1	1	0	1	0	0	0	1
12	B	1	1	1	0	0	0	0	0	0	0	0	1	0	1	1	0	0	0	1
13	B	1	1	1	0	1	1	2	2	1	1	1	1	1	0	0	1	1	0	1
14	B	1	0	0	1	2	2	2	2	0	0	2	2	1	1	1	0	0	0	1
15	B	1	1	0	0	1	1	1	1	0	0	1	1	0	1	1	0	0	0	1
16	B	1	0	0	1	1	1	2	2	1	1	1	0	0	0	0	1	0	1	1
17	B	1	1	1	0	0	0	1	1	0	0	1	1	1	0	1	0	0	0	1
18	B	1	1	1	0	0	0	0	0	0	0	0	1	1	0	1	0	0	0	1
19	B	1	1	2	0	0	0	2	2	0	0	2	2	0	2	1	0	0	0	1
20	B	1	0	0	1	1	1	2	2	1	1	1	0	0	0	0	1	1	0	1
21	B	1	1	0	0	1	1	1	1	1	1	0	0	0	0	0	1	0	1	1
22	B	1	0	0	1	1	0	0	0	0	0	0	0	0	0	1	0	0	0	0
23	B	1	0	0	1	2	2	2	2	1	1	1	1	1	0	0	1	0	1	1
24	B	1	1	1	0	1	1	1	1	0	0	1	1	1	1	1	0	0	0	1
25	B	1	1	1	0	1	1	1	1	1	1	0	1	0	0	0	1	0	1	1
27	A	1	0	0	1	1	0	1	1	1	1	0	0	0	0	0	1	0	1	1
28	B	1	0	0	1	1	1	2	2	0	0	2	1	0	1	1	0	0	0	1
29	B	1	1	1	0	1	1	2	2	1	1	1	1	1	0	0	1	0	1	1
30	B	1	1	1	0	0	0	2	2	1	1	1	0	0	0	0	1	0	1	1
31	B	1	0	1	0	1	1	1	1	1	1	0	1	0	1	0	1	1	0	1
33	B	1	0	0	1	1	0	0	0	0	0	0	0	0	0	1	0	0	0	1
34	A	1	0	0	1	0	1	0	0	0	0	0	0	0	0	0	0	0	0	1
36	B	1	1	1	0	2	2	2	2	0	0	2	1	2	0	1	0	0	0	1
38	B	1	0	0	1	2	2	2	2	0	0	2	1	0	1	1	0	0	0	1
40	B	1	1	1	0	2	2	1	1	0	0	1	2	1	1	0	0	0	0	1
41	B	1	1	1	0	1	2	1	1	0	0	1	2	2	0	1	0	0	0	1

Continued on the following page

Table S2. Definitions of KIR haplotypes.

KIRhaplotype	AvsB	KIR3DL3	KIR2DS2	KIR2DL2	KIR2DL3	KIR2DP1	KIR2DL1	KIR3DP1	KIR2DL4	KIR3DL1ex4	KIR3DL1ex9	KIR3DS1	KIR2DL5	KIR2DS3	KIR2DS5	KIR2DS1	KIR2DS4TOTAL	KIR2DS4WT	KIR2DS4DEL	KIR3DL2
42	B	1	1	2	0	0	0	2	2	1	1	1	1	0	1	0	1	0	1	1
44	B	1	1	1	0	1	1	1	1	0	0	1	2	0	1	1	0	0	0	1
45	B	1	1	1	0	1	1	1	1	0	0	1	2	1	1	0	0	0	0	1
46	B	1	0	1	0	1	1	1	1	0	0	1	1	1	1	1	0	0	0	1
48	A	1	0	0	1	1	1	1	1	1	2	0	0	0	0	0	2	2	0	1
50	B	1	0	0	0	1	1	1	1	1	1	0	1	1	0	0	1	1	0	1
52	B	1	0	0	0	1	1	1	1	1	1	0	1	0	1	0	1	0	1	1
53	B	1	1	0	1	1	1	1	1	1	1	0	0	0	0	0	1	0	1	1
55	A	1	0	0	0	1	2	1	1	1	1	0	0	0	0	0	1	1	0	1
56	B	1	1	1	0	0	0	0	1	0	0	1	1	0	1	1	0	0	0	1
57	A	1	0	0	1	1	1	0	1	1	1	0	0	0	0	0	1	0	1	1
58	A	1	0	0	1	1	1	1	0	1	1	0	0	0	0	0	1	1	0	1
59	A	1	0	0	1	1	1	1	1	1	0	0	0	0	0	0	0	0	0	1
68	B	1	1	1	0	0	0	0	1	1	1	0	0	0	0	0	1	0	1	1
69	B	1	1	1	0	0	0	0	0	0	0	0	1	0	0	1	0	0	0	1

The relationship between the different KIR types in our data. Each column represents a KIR type: *KIRhaplotype* is the haplotype classification defined by Jiang *et al.*[2], *AvsB* corresponds to the broad A/B haplotype group classification, and the other columns are the copy number of individual KIR genes. Each *KIRhaplotype* is uniquely defined by the copy number values across the 17 KIR loci.

Table S3. KIR allele frequencies.

(A) UK fine-scale haplotypes

KIRhaplotype	**Count**
1	239
2	57
3	47
4	27
5	26
6	22
7	7
8	7
9	8
10	9
11	9
12	4
13	1
14	2
16	1
18	2
19	1
20	1
21	1
25	1
27	1
42	1
44	1
48	1
55	1
56	1
69	1

(B) UK A/B haplotypes

AvsB	**Count**
A	299
B	180

(C) UK copy number types

**Gene**	**0**	**1**	**2**
KIR3DL3	0	479	0
KIR2DS2	359	120	0
KIR2DL2	360	117	2
KIR2DL3	121	358	0
KIR2DP1	70	407	2
KIR2DL1	71	405	3
KIR3DP1	8	464	7
KIR2DL4	7	465	7
KIR3DL1ex4	90	389	0
KIR3DL1ex9	90	388	1
KIR3DS1	392	84	3
KIR2DL5	355	105	19
KIR2DS3	419	52	8
KIR2DS5	408	70	1
KIR2DS1	389	90	0
KIR2DS4TOTAL	90	388	1
KIR2DS4WT	383	95	1
KIR2DS4DEL	186	293	0
KIR3DL2	0	479	0

(D) NG copy number types

**Gene**	**0**	**1**	**2**	**3**	**4**	**Null**
KIR3DL3	0	1	1330	7	0	0
KIR2DS2	702	526	108	0	0	2
KIR2DL2	710	510	113	4	0	1
KIR2DL3	111	524	697	4	0	2
KIR2DP1	42	346	933	15	1	1
KIR2DL1	46	383	894	14	1	0
KIR3DP1	1	55	1213	57	3	9
KIR2DL4	1	58	1210	59	3	7
KIR3DL1ex4	62	439	831	6	0	0
KIR3DL1ex9	62	441	835	0	0	0
KIR3DS1	856	403	72	6	0	1
KIR2DL5	724	404	180	27	3	0
KIR2DS3	1003	267	63	5	0	0
KIR2DS5	941	349	45	3	0	0
KIR2DS1	839	437	61	1	0	0
KIR2DS4TOTAL	62	441	835	0	0	0
KIR2DS4WT	814	463	61	0	0	0
KIR2DS4DEL	244	662	432	0	0	0
KIR3DL2	1	38	1299	0	0	0

Sample counts for the KIR types in the reference panels. For the UK reference panel: (**A**) fine-scale KIR haplotypes (*KIRhaplotype*); (**B**) A/B haplotypes (*AvsB*); (**C**) copy number, per-haplotype. For the NG validation panel: (**D**) copy number, per-individual. For the copy number sub-tables (**C–D**), each row represents a KIR locus, each column a particular copy number, with the sample counts in each cell. The 'Null' column shows the number of individuals with missing data for each locus.

Table S4. SNP tagging.

Locus	Tag SNP	LD (r^2)	Accuracy (%)
AvsB	rs587560	0.53	86.8
KIR2DS2	rs587560	0.95	99.0
KIR2DL2	rs587560	0.92	98.3
KIR2DL3	rs587560	0.93	98.8
KIR2DP1	rs587560	0.47	88.5
KIR2DL1	rs587560	0.45	88.1
KIR3DP1	seq-rs1010355	0.00	96.9
KIR2DL4	seq-rs1010355	0.00	97.1
KIR3DL1ex4	seq-rs592645	0.93	99.0
KIR3DL1ex9	seq-rs592645	0.92	98.8
KIR3DS1	seq-rs592645	0.89	98.1
KIR2DL5	seq-t1d-19-60034052-C-T	0.67	90.6
KIR2DS3	seq-rs1010355	0.00	87.5
KIR2DS5	seq-rs592645	0.67	94.8
KIR2DS1	seq-rs592645	0.93	99.0
KIR2DS4TOTAL	seq-rs592645	0.92	98.8
KIR2DS4WT	rs4806585	0.73	96.0
KIR2DS4DEL	rs581623	0.95	98.8

The best tag SNP for each copy number locus and *AvsB*. The SNPs were selected to maximise prediction accuracy (see Material and Methods), which is shown in the final column. Also reported is the square of the Pearson correlation coefficient, a measure of the linkage disequilibrium (LD) with the target locus. Note that the accuracy estimates shown here can differ slightly to those shown in the methods comparison (see Material and Methods).

Table S5. KIR imputation with other SNP arrays.

Manufacturer	Array	SNPs in ***UKsnps***	**KIRhaplotype**	**AvsB**	**KIR2DS2**	**KIR2DL2**	**KIR2DL3**	**KIR2DP1**	**KIR2DL1**	**KIR3DP1**
Illumina	**Immunochip**	**301**	**86.8**	**98.5**	**99.0**	**98.1**	**98.8**	**91.9**	**91.4**	**96.9**
	Human1M-Duo v3.0	109	84.5	97.1	98.8	98.1	98.5	90.4	91.0	96.9
	Human610-Quad v1.0	70	79.5	94.2	95.8	95.0	95.6	88.3	87.9	96.9
	Human660W-Quad v1.0	68	79.8	94.4	95.8	95.2	95.4	88.7	87.7	96.9
	HumanCore-12 v1.0	26	72.2	78.3	74.5	74.5	74.3	85.0	84.8	96.7
	HumanCore-24 v1.0	26	64.5	71.6	74.5	75.4	74.7	84.8	84.8	96.9
	HumanCoreExome-24 v1.0	30	64.5	72.4	74.3	73.9	72.4	84.5	84.1	96.9
	HumanOmni1S-8 v1.0	54	73.7	86.4	77.5	77.9	77.9	86.8	86.4	96.9
	HumanOmni2.5-Quad	145	86.6	98.5	99.0	98.3	98.8	91.7	92.3	96.9
	HumanOmni2-5-8 v1.2	134	83.5	98.5	99.0	98.3	98.8	91.2	91.7	97.1
	HumanOmni2.5S-8 v1.0	58	73.3	92.3	95.6	94.8	95.4	90.2	90.0	96.2
	HumanOmni5-4 v1.1	164	83.1	98.5	99.0	98.3	98.8	91.9	91.7	96.9
	HumanOmniExpress-24 v1.1	77	80.2	94.0	99.0	98.3	98.8	90.8	90.2	96.9
	HumanOmniExpressExome-8 v1.2	82	85.8	98.3	99.0	98.3	98.8	91.0	90.4	96.9
	HumanOmniExpressExome-8 v1.0	78	86.2	98.5	99.0	98.1	98.8	91.4	91.7	96.9
Affymetrix	Genome-Wide Human 5.0	23	52.4	71.2	77.0	76.6	77.5	86.0	85.0	96.7
	Genome-Wide Human 6.0	52	66.6	85.0	80.6	81.0	81.2	86.4	85.4	96.7
	GeneChip Human Mapping 10K 2.0	1	49.9	62.4	75.0	75.2	74.7	85.0	84.5	96.9
	GeneChip Human Mapping 100K Set	7	49.9	62.4	75.0	75.2	74.7	85.0	84.5	96.9
	GeneChip Human Mapping 500K Set	26	62.0	81.6	77.2	78.9	78.7	86.8	86.2	96.2
	Axiom Biobank	34	60.8	76.6	74.5	75.0	74.3	85.0	85.0	96.7
	Axiom Exome 319	10	56.0	77.5	74.7	75.0	74.5	84.8	84.3	96.7
	Axiom Genome-Wide EUR 1	52	74.3	85.4	79.1	79.1	78.7	86.2	86.2	96.7
	UK Biobank Axiom	241	81.6	95.8	95.8	95.0	95.6	89.8	88.3	96.9

Continued on the following page

Table S5. KIR imputation with other SNP arrays.

Manufacturer	Array	KIR2DL4	KIR3DL1ex4	KIR3DL1ex9	KIR3DS1	KIR2DL5	KIR2DS3	KIR2DS5	KIR2DS1	KIR2DS4TOTAL	KIR2DS4WT	KIR2DS4DEL
Illumina	**Immunochip**	**97.1**	**99.0**	**98.8**	**97.5**	**92.7**	**91.4**	**96.5**	**99.0**	**98.8**	**99.6**	**98.8**
	Human1M-Duo v3.0	97.1	97.1	96.9	96.5	88.9	90.6	96.2	97.1	97.1	98.3	98.8
	Human610-Quad v1.0	97.1	97.1	96.7	96.5	88.3	89.6	96.0	96.9	96.7	97.5	98.8
	Human660W-Quad v1.0	97.1	96.9	96.7	96.2	87.9	88.9	95.6	96.9	96.7	97.5	98.8
	HumanCore-12 v1.0	96.9	97.1	96.9	96.0	86.6	88.1	96.2	97.1	97.1	98.1	98.3
	HumanCore-24 v1.0	97.1	88.7	88.3	88.5	80.8	88.3	91.2	88.9	88.1	95.0	85.0
	HumanCoreExome-24 v1.0	97.1	89.3	88.5	89.3	81.4	87.3	91.7	89.3	88.1	95.2	85.4
	HumanOmni1S-8 v1.0	97.1	98.8	98.5	97.7	90.6	88.9	95.0	98.8	98.5	98.5	96.7
	HumanOmni2.5-Quad	97.1	98.3	98.3	97.9	92.9	91.2	96.5	98.5	98.3	99.2	98.8
	HumanOmni2-5-8 v1.2	97.3	98.1	97.9	97.7	92.7	91.0	96.9	98.1	97.9	96.5	97.7
	HumanOmni2.5S-8 v1.0	96.7	94.6	94.4	92.9	88.3	90.6	92.1	94.4	94.2	96.5	93.3
	HumanOmni5-4 v1.1	97.1	98.1	97.9	97.3	92.7	90.8	97.1	97.9	97.9	96.7	98.3
	HumanOmniExpress-24 v1.1	97.1	95.0	94.6	92.1	88.1	90.8	92.5	95.4	94.8	95.8	95.0
	HumanOmniExpressExome-8 v1.2	97.1	97.7	97.7	97.9	91.0	91.4	96.2	97.7	97.7	98.5	98.8
	HumanOmniExpressExome-8 v1.0	97.1	97.9	97.5	98.1	91.2	90.2	96.5	97.9	97.5	98.1	98.8
Affymetrix	Genome-Wide Human 5.0	96.7	79.1	78.9	79.3	72.4	85.6	83.1	79.3	79.1	81.4	80.8
	Genome-Wide Human 6.0	97.1	97.7	97.7	96.5	86.6	88.7	93.5	97.5	97.5	88.5	86.4
	GeneChip Human Mapping 10K 2.0	97.1	81.2	81.0	81.8	74.1	87.5	85.2	81.2	81.0	80.0	61.2
	GeneChip Human Mapping 100K Set	97.1	81.2	81.0	81.8	74.1	87.5	85.2	81.2	81.0	80.0	56.0
	GeneChip Human Mapping 500K Set	96.9	97.3	97.1	96.5	86.2	85.6	93.7	97.5	97.3	86.6	85.2
	Axiom Biobank	96.9	92.7	92.7	92.5	83.9	88.5	92.1	92.7	92.7	84.3	78.1
	Axiom Exome 319	96.9	91.9	92.1	91.9	84.3	87.5	86.2	92.1	92.3	81.2	73.9
	Axiom Genome-Wide EUR 1	96.9	98.3	98.3	97.5	88.1	88.1	95.4	98.5	98.1	98.8	98.8
	UK Biobank Axiom	97.1	99.0	98.5	97.5	91.2	89.8	96.0	98.8	98.8	99.6	98.8

Table description on the following page

Table S5. KIR imputation with other SNP arrays.

Estimates of the best-case imputation accuracy (%) for various SNP genotyping arrays, under the assumption that all SNPs in common with the Immunochip would be typed perfectly in a study sample. The estimates are OOB accuracy from a model fitted with the subset of the *UKsnps* set that are present on each array (the exact number is reported in the 3rd column on the first page of the table). All models are fitted using the UK reference panel, which is typed on the Immunochip; the results for the Immunochip are shown in bold as a reminder of this fact. Given the Immunochip-specific nature of this analysis, these results cannot be interpreted as measuring the absolute potential of each array for studying the KIR region. For this reason, we caution against any comparisons of these results between arrays. Rather, we suggest using this as a guide to the potential of using KIR*IMP with the current reference panel for any existing data typed on a given array.

Supplemental References

[1] Carrington, M. and Norman, P. (2003). The KIR Gene Cluster. (National Center for Biotechnology Information (US)).

[2] Jiang, W., Johnson, C., Jayaraman, J., Simecek, N., Noble, J., Moffatt, M. F., Cookson, W. O., Trowsdale, J., and Traherne, J. A. (2012). Copy number variation leads to considerable diversity for B but not A haplotypes of the human KIR genes encoding NK cell receptors. Genome Research *22*, 1845–1854.
